# Single Molecule Magnetism with Strong Magnetic Anisotropy and Enhanced Dy∙∙∙Dy Coupling in Three Isomers of Dy‐Oxide Clusterfullerene Dy_2_O@C_82_

**DOI:** 10.1002/advs.201901352

**Published:** 2019-08-15

**Authors:** Wei Yang, Georgios Velkos, Fupin Liu, Svetlana M. Sudarkova, Yaofeng Wang, Jiaxin Zhuang, Hanning Zhang, Xiang Li, Xingxing Zhang, Bernd Büchner, Stanislav M. Avdoshenko, Alexey A. Popov, Ning Chen

**Affiliations:** ^1^ College of Chemistry Chemical Engineering and Materials Science Soochow University Suzhou Jiangsu 215123 P. R. China; ^2^ Leibniz Institute for Solid State and Materials Research (IFW Dresden) Helmholtzstrasse 20 01069 Dresden Germany

**Keywords:** antiferromagnetic, dysprosium, exchange interactions, metallofullerene, oxide, single molecule magnets

## Abstract

A new class of single‐molecule magnets (SMMs) based on Dy‐oxide clusterfullerenes is synthesized. Three isomers of Dy_2_O@C_82_ with *C*_s_(6), *C*_3v_(8), and *C*_2v_(9) cage symmetries are characterized by single‐crystal X‐ray diffraction, which shows that the endohedral Dy−(µ_2_‐O)−Dy cluster has bent shape with very short Dy−O bonds. Dy_2_O@C_82_ isomers show SMM behavior with broad magnetic hysteresis, but the temperature and magnetization relaxation depend strongly on the fullerene cage. The short Dy−O distances and the large negative charge of the oxide ion in Dy_2_O@C_82_ result in the very strong magnetic anisotropy of Dy ions. Their magnetic moments are aligned along the Dy−O bonds and are antiferromagnetically (AFM) coupled. At low temperatures, relaxation of magnetization in Dy_2_O@C_82_ proceeds via the ferromagnetically (FM)‐coupled excited state, giving Arrhenius behavior with the effective barriers equal to the AFM‐FM energy difference. The AFM‐FM energy differences of 5.4–12.9 cm^−1^ in Dy_2_O@C_82_ are considerably larger than in SMMs with {Dy_2_O_2_} bridges, and the Dy∙∙∙Dy exchange coupling in Dy_2_O@C_82_ is the strongest among all dinuclear Dy SMMs with diamagnetic bridges. Dy‐oxide clusterfullerenes provide a playground for the further tuning of molecular magnetism via variation of the size and shape of the fullerene cage.

## Graphical abstract

**The smallest fragment of dysprosium oxide is stabilized inside a fullerene.** Three isomers of oxide clusterfullerenes Dy_2_O@C_82_ are synthesized and characterized by single‐crystal X‐ray diffraction. The compact size of the Dy_2_O cluster leads to unusually short Dy—O bonds, strong magnetic anisotropy, and enhanced antiferromagnetic interactions between Dy ions. Dy_2_O@C_82_ isomers behave as single molecule magnets with broad magnetic hysteresis.

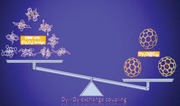

## 1 Introduction

The ability of fullerenes to stabilize the species which can hardly exist otherwise has been extensively used to create a number of endohedral metallofullerene (EMF) families.^1^ Among them, a great variety of clusterfullerenes,[qv: 1b] i.e., EMFs combining metal atoms with nonmetal endohedral species, has been obtained since the discovery of the first clusterfullerene, Sc_3_N@C_80_, in 1999.^2^ Endohedral units in clusterfullerenes comprise from one to four metal atoms (denoted as “M” in the following discussion), typically in their three‐valent state, and electronegative atoms or species, which attain a negative charge and compensate Coulomb repulsion of positively charged metal ions. The clusterfullerenes are then titled according to the negatively charge units, such as “nitride clusterfullerenes” for EMFs with the nitride ion N^3−^ in the center of the M_3_N endohedral cluster,^3^ or “sulfide clusterfullerenes” with the sulfide ion S^2−^ in the M_2_S cluster.^4^ Other well‐studied types of clusterfullerenes include carbide clusterfullerenes (M_2_C_2_,^5^ M_3_C_2_,^6^ M_2_TiC_1,2_,^7^ or Sc_4_C_2_ endohedral units^8^), carbonitride clusterfullerene (MCN^9^ and Sc_3_CN^10^), Sc_3_CH@C_80_,^11^ and oxide clusterfullerenes.^12^

Oxide clusterfullerenes were first discovered in 2008 by Stevenson et al., who reported the isolation of Sc_4_O_2_@C_80_ and Sc_4_O_3_@C_80_ when the arc‐discharge synthesis of Sc‐EMFs was performed in the presence of Cu(NO_3_)_2_.^13^ Unusual structures of these molecules comprise Sc_4_ tetrahedrons with two or three oxygen atoms in µ_3_‐coordination above Sc_3_‐faces. The same group soon isolated another type of oxide clusterfullerene, Sc_2_O@*C*_s_(6)‐C_82_, in which the Sc—O—Sc cluster has a bent shape with µ_2_‐coordinated oxide ion.^14^ Adding CO_2_ to the atmosphere in the arc‐discharge reactor, Feng and Chen et al. obtained and structurally characterized a series of Sc_2_O@C_2_ *_n_* molecules with 2*n* ranging from 70 to 82.^15^ The formal charge distribution in these clusterfullerenes can be described as (Sc^3+^)_2_O^2−^@C_2_ *_n_* ^4−^, and therefore the preferable fullerene cages for encapsulation of Sc_2_O are those, which form stable tetraanions. Compared to the extensively studied Sc_2_O@C_2_ *_n_*, the information on the oxide clusterfullerenes of metals other than Sc is very scarce. Mixed‐metal ScGdO@C_82_ was synthesized and characterized spectroscopically.^16^ Formation of M_2_O@C_2n_ species for M = Y, Lu was proved by mass‐spectrometry,[qv: 12b] but none of them has been isolated so far. Very recently, Ho_2_O@C_74_ was obtained and its molecular structure with an almost linear Ho_2_O cluster was elucidated by single‐crystal X‐ray diffraction.^17^

In this work, we report on the synthesis and characterization of the first Dy‐oxide clusterfullerenes. The recent interest in Dy‐EMFs is caused by their robust magnetic properties.^18^ In Dy‐clusterfullerenes, a short distance between metal ions and negatively charged nonmetal species creates a strong axial ligand field and hence a strong magnetic anisotropy for the Dy ions.[qv: 4c,19] Besides, with 4f^9^ electrons, Dy^3+^ is a Kramers ion, which ensures that its ground magnetic state is a doublet with orthogonal wave functions. This combination of a bistable magnetic ground state and a strong single‐ion magnetic anisotropy are essential for the single molecule magnetism (SMM).^20^ Indeed, a long relaxation of magnetization was proven first for DySc_2_N@C_80_ in 2012,^21^ and since then the SMM behavior was established for Dy‐nitride,[qv: 19c,22] Dy‐sulfide,[qv: 4c] and Dy‐carbide[qv: 4c,7b,23] clusterfullerenes as well as dimetallofullerenes.[qv: 18b,24] Furthermore, a possibility to combine two or more Dy ions within one fullerene molecule opens an additional degree of freedom for tuning magnetic properties of Dy‐EMFs by designing exchange interactions between the lanthanide ions.[qv: 22b] Finally, the carbon cage hosting Dy cluster also plays a certain but yet not fully understood role in the relaxation of magnetization in EMFs, and significant influence of the fullerene isomerism on the SMM characteristics was found for nitride and sulfide clusterfullerenes.[qv: 4c,22a]

The nitride clusterfullerene Dy_2_ScN@C_80_‐*I_h_* has been the strongest SMM among the clusterfullerenes so far. It has the blocking of magnetization at *T*_B_ = 8 K, and above 60 K its spin relaxation follows the Orbach mechanism with the thermal barrier of 1206 ± 15 cm^−1^ corresponding to the 5th Kramers doublet of Dy ions.[qv: 19c] The computational studies revealed that Dy‐oxide clusterfullerenes may have superior SMM properties with thermal barriers up to 1400 cm^−1^.[qv: 19e] Here we provide the first experimental study of the magnetic properties of three isomers of Dy_2_O@C_82_. We demonstrate that Dy_2_O‐clusterullerenes are excellent single molecule magnets exhibiting broad magnetic hysteresis and the strongest superexchange coupling between Dy ions ever reported for nonradical bridged compounds.

## 2 Results and Discussion

### 2.1 Synthesis, Isolation, and Cage Isomeric Structures of Three Isomers of Dy_2_O@C_82_

**Figure 1:**
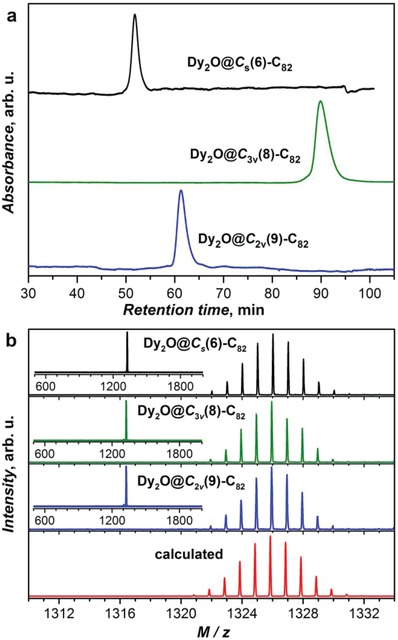
a) HPLC chromatograms of isolated Dy_2_O@*C*_s_(6)‐C_82_, Dy_2_O@*C*_3v_(8)‐C_82_, and Dy_2_O@*C*_2v_(9)‐C_82_ obtained on a 10 mm × 250 mm Buckyprep column with λ = 310 nm, a flow rate of 4.0 mL min^−1^, and toluene as the mobile phase. b) The corresponding experimental and theoretical mass‐spectra and isotopic distributions of Dy_2_O@C_82_.

Dy_2_O@C_82_ (*C*_s_(6), *C*_3v_(8), *C*_2v_(9)) were synthesized by a modified Krätschmer‐Huffman DC arc‐discharge method under a He/CO_2_ atmosphere (200 Torr of helium with 20 Torr of CO_2_ added).^25^ The soot was collected and refluxed in carbon disulfide (CS_2_) under an argon atmosphere for 12 h. The crude extract was treated with TiCl_4_ and most of the empty fullerenes were removed (Figure S1, Supporting Information).^26^ Dy_2_O@C_82_ isomers with *C*_s_(6), *C*_3v_(8), and *C*_2v_(9) fullerene cages were isolated and purified by multistage high‐performance liquid chromatography (HPLC) (Figures S2–S4, Supporting Information). In **Figure** **1**, the purity of each sample is confirmed by the single peak on the final‐stage HPLC chromatograms and the positive mode matrix‐assisted laser desorption/ionization‐time‐of‐flight mass spectrometry (MALDI‐TOF MS). Further, the isotopic distribution of the experimental MS spectra of all samples agrees well with a theoretical simulation for Dy_2_O@C_82_, which confirms their molecular formulas.

### 2.2 Single‐Crystal X‐Ray Diffraction

**Figure 2:**
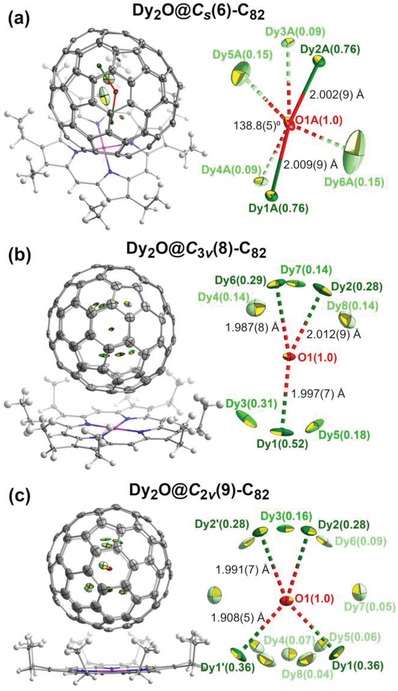
Single‐crystal X‐ray structure of Dy_2_O@C_82_ isomers co‐crystallized with Ni(OEP): a) Dy_2_O@*C*_s_(6)‐C_82_; b) Dy_2_O@*C*_3v_(8)‐C_82_; c) Dy_2_O@*C*_2v_(9)‐C_82_. For each isomer, the structure of Dy_2_O@C_82_ ∙ Ni(OEP) is shown on the left (structures are oriented so that molecular symmetry plane is parallel to the paper), and enlargement of the endohedral Dy_2_O unit with disordered Dy sites and selected structural parameters is shown on the right. The brightness of the color differentiates the site occupancies (the darker the color, the higher the occupancy). Solvent molecules are omitted for clarity. The displacement parameters are shown at the 10% probability level. Color code: grey for carbon, green for Dy, red for O, blue for N, white for H, and purple for Ni.

Crystals suitable for X‐ray diffraction analysis were grown by layering the benzene solution of nickel (II) octaethylporphyrin (Ni(OEP)) onto the CS_2_ solution of the Dy_2_O@C_82_ isomers. X‐ray diffraction of Dy_2_O@*C*_s_(6)‐C_82_∙Ni(OEP)∙2(C_6_H_6_) was measured at 100 K using the wavelength of 0.82653 Å with a CCD detector at the beamline BL17B of the Shanghai Synchrotron Radiation Facility (SSRF); Dy_2_O@*C*_3v_(8)‐C_82_∙Ni(OEP)∙1.5(C_6_H_6_)∙CS_2_ and Dy_2_O@*C*_2v_(9)‐C_82_∙Ni(OEP)∙C_6_H_6_ were measured with Bruker APEX II at room temperature and 173 K, respectively. The structures were solved by direct methods and refined using all data (based on F^2^) by SHELX 2016.^27^ Hydrogen atoms were located in a difference map, added geometrically, and refined with a riding model. The crystal data are presented in Table S1 (Supporting Information). The data can be obtained free of charge from the Cambridge Crystallographic Data Centre with CCDC Nos. 1908347–9. **Figure** **2** shows the structures of the fullerenes and their mutual orientations with the co‐crystallized Ni(OEP) molecules. The shortest fullerene cage to Ni(OEP) contact is 2.90(2), 2.87(2), and 2.77(2) Å for *C*_s_, *C*_3v_, and *C*_2v_ isomers, respectively, which is similar to the previous reports.[qv: 4c,14,15]

The asymmetric unit of Dy_2_O@*C*_s_(6)‐C_82_∙Ni(OEP)∙2(C_6_H_6_) contains two fullerene sites A and B, as well as two Ni(OEP) sites. This phenomenon was already reported for two similar crystals Sc_2_S@*C*_s_(6)‐C_82_∙Ni(OEP)∙2(C_6_H_6_)^28^ and Tb_2_C_2_@*C*_s_(6)‐C_82_∙Ni(OEP)∙2(C_6_H_6_),^29^ which have the same fullerene skeleton and similar shape of the endohedral cluster. Figure 2a shows the site A, which features a fully ordered fullerene cage, a single site for the endohedral oxygen atom, and a well‐ordered encapsulated Dy_2_O cluster with the major site occupancies of Dy of 0.76 and two minor sites with occupancy of 0.15 and 0.09. In the site B the Dy_2_O cluster is less ordered with the site occupancies of 0.52, 0.29, and 0.19 (Figures S5 and S6, Supporting Information). The cage and oxygen sites also show some sign of disorder, although the structure still can be modeled satisfactorily using a single cage orientation. Structural parameters of the encapsulated Dy_2_O cluster can be determined reliably from the major site in the well‐ordered structure A. The Dy—O bond lengths are 2.009(9) and 2.002(9) Å, and the Dy—O—Dy bond angle is 138.8(5)°.

In Dy_2_O@*C*_3v_(8)‐C_82_∙Ni(OEP)∙1.5(C_6_H_6_)∙CS_2_, the asymmetric unit contains one intact Ni(OEP) and one fullerene molecule with ordered carbon cage. The encapsulated Dy_2_O is strongly disordered, with 8 sites refined for Dy with occupancies ranging from 0.518(3) to 0.137(3) as shown in Figure 2b. The asymmetric unit of Dy_2_O@*C*_2v_(9)‐C_82_∙Ni(OEP)∙C_6_H_6_ contains a half of the fullerene molecule and a half of the Ni(OEP) molecule. The crystallographic mirror plane bisects both the Ni(OEP) and the fullerene cage through the mirror plane of the molecules. Oxygen atom is refined as a single site located on the crystallographic mirror plane near the center of the carbon cage. Dy atoms are severely disordered with 14 refined sites (some of them related via crystallographic mirror plane, Figure 2c) with occupancies ranging from 0.360(2) to 0.041(2).

**Figure 3:**
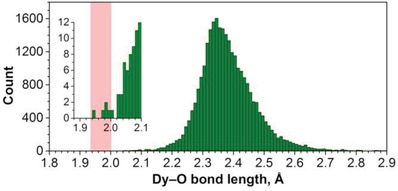
Distribution of experimental Dy—O bond lengths in molecular compounds based on 28 533 entries from the CSD database with additional data from this work. Step size in the histogram is 0.01 Å, the inset magnifies the range between 1.88 and 2.10 Å (abscissa scale is the same as in the main graph); the range of Dy—O bond lengths in Dy_2_O@C_82_ found in this work is highlighted as a pink rectangle.

A strong disorder of the Dy_2_O cluster positions in Dy_2_O@*C*_3v_(8)‐C_82_ and Dy_2_O@*C*_2v_(9)‐C_82_ precludes precise determination of the cluster structural parameters for these isomers. Average Dy—O distances (weighted with Dy site occupancies) are 1.978(9) and 1.944(8) Å in *C*_3v_(8) and *C*_2v_(9)‐C_82_ isomers. The average Dy—O distances in Dy_2_O@*C*_s_(6)‐C_82_ isomer are 1.985(11) Å for the site A and 1.939(12) Å for the less ordered site B, which shows that the disorder in the structure reduces the apparent Dy—O distances. Thus, based on the most reliable parameters determined for the site A of Dy_2_O@*C*_s_(6)‐C_82_, we conclude that the Dy—O bond lengths in Dy_2_O@C_82_ are close to 2.0 Å. This value is much shorter than 2.30 Å, which might be the expected based on the covalent radii of Dy (1.67 Å) and O (0.63 Å).^30^ Analysis of 28 533 entries in the Cambridge Structural Database (CSD) shows that Dy—O bonds in Dy_2_O@C_82_ are indeed among the shortest ever reported for molecular Dy compounds (**Figure** **3**; see also Ref. 31 for an analysis of correlations in lanthanide‐oxygen bond lengths). The vast majority of Dy—O bond lengths falls into the range of 2.1–2.7 Å, with the maximum between 2.3–2.4 Å. The compounds with Dy—O bond lengths shorter than 2.1 Å are quite rare and are all listed in Table S2 (Supporting Information). The Dy—O bonds shorter than in Dy_2_O@C_82_ are found in only one compound, [Dy_5_O(O*^i^*Pr)_13_], featuring Dy_5_ pyramids with the shortest Dy—O bond of 1.951(18) Å.^32^

All three fullerene cage isomers found in this work for Dy_2_O@C_82_ are rather common and occur in different types of EMFs, including oxide,^14^, ^15^ sulfide,[qv: 4c,28] carbide,^29^, ^33^ nitride,^34^ carbonitride[qv: 9a,c] clusterfullerenes as well as monometallo^35^ and dimetallofullerenes.[qv: 33b,36] A complete list of all 39 EMFs with *C*_s_(6)‐C_82_, *C*_3v_(8)‐C_82_, and *C*_2v_(9)‐C_82_ cages characterized by single‐crystal X‐ray diffraction in the form of co‐crystals with M(OEP) (M = Ni, Co) is given in Table S3 and Figures S7–S46 (Supporting Information). Analysis of these crystal structures shows that the orientation of the same cage isomer toward the porphyrin moiety is not fixed but can vary from one EMF to another. For 17 EMF structures with *C*_s_(6)‐C_82_ cage, we found four different ways how the cage is oriented with respect to Ni(OEP). The EMF∙Ni(OEP) orientation found in this work for Dy_2_O@*C*_s_(6)‐C_82_ is similar to several other EMFs, including carbides, sulfides, and dimetallofullerenes. Eight EMFs with the *C*_3v_(8) cage reported so far show three different orientation types. The cage‐Ni(OEP) orientation in Dy_2_O@*C*_3v_(8)‐C_82_∙Ni(OEP) appears to be unique and does not fit any of these three. The EMFs with C_2v_(9)‐C_82_ cage demonstrate the largest structural diversity with 6 orientations in 14 reported crystal structures. The Dy_2_O@C_2v_(9)‐C_82_ system adds to this diversity yet another unique cage‐Ni(OEP) orientation.

### 2.3 Density Functional Theory (DFT) Calculations and Molecular Dynamics

Whereas the fullerene cage structures are unambiguously elucidated by single‐crystal X‐ray diffraction, the strong disorder in positions of Dy atoms does not allow precise determination of the endohedral cluster positions. Therefore, we performed DFT calculations to find the preferable geometries and orientations of the Dy_2_O clusters inside the fullerenes. As the endohedral clusters may have multiple energy minima inside the carbon cages, it is important to have a comprehensive sampling including all possible structural configurations. We have recently shown that rotation of the endohedral cluster along the Fibonacci sphere nodes provides such efficient and homogenous sampling for structural studies of EMFs.^37^ Following this approach, 120 initial configurations (conformers) were generated for each cage isomer and then optimized at the PBE/TZ2P level. At this stage, computations were performed with Priroda code^38^ with Y as a model of Dy. At the second stage, unique conformers found in the first step were re‐optimized for Dy_2_O@C_82_ at the PBE‐D level with PAW potentials using the VASP 5.0 code.^39^ Besides, several sets of starting coordinates for optimization were also generated using the detected cluster sites from X‐ray data. The study resulted in seven unique conformers for *C*_s_(6) (Figure S49, Supporting Information), five for *C*_3v_(8) (Figure S51, Supporting Information), and eight for *C*_2v_(9) (Figure S53, Supporting Information). Note that the results of the calculations for Y_2_O@C_82_ and re‐optimization results for Dy_2_O@C_82_ are quite close to each other (Table S4–S6, Supporting Information), which confirms once again that Y is a good model for Dy in computational studies of EMFs.

**Figure 4:**
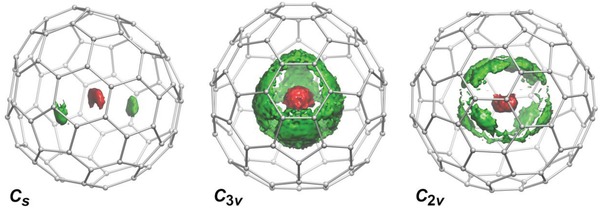
Spatial distribution of the probability density for Dy and O atoms in Dy_2_O@C_82_ isomers as determined from molecular dynamics simulations at *T* = 300 K. Displacements of carbon atoms are not shown.

The static DFT optimizations allow finding of the energy minima, but not provide sufficient information on the internal dynamics in the molecule. The presence of several energetically close minima may indicate a flat potential energy surface, which in due turn may result in complex internal dynamics of the endohedral cluster and its freezing in multiple conformers when the compound is cooled down. DFT‐based Born‐Oppenheimer molecular dynamics (BOMD) simulations were performed to shed more light onto this question. All BOMD calculations were performed for Y_2_O@C_82_ analogs with atomic masses of Dy assigned to Y. Single point energies and forces were calculated at the PBE/TZ2P level using Priroda code. These forces were used to propagate the system in the canonical ensemble (NVT) using Nose‐Hoover algorithm as implemented in the Python Atomic Simulation Environment libraries (ASE 3.0).^40^ The thermostat temperature was set to 300 K with the characteristic coupling time of 10 fs. All unique conformers within 20 kJ mol^−1^ for each isomer (one for *C*_s_, six for *C*_3v_, and seven for *C*_2v_) were propagated with time step 1 fs for 18–20 ps using the initial conformers geometries as starting points and initial velocities assigned randomly from Maxwell‐Boltzmann distribution at 300 K. For the sole conformer of *C*_s_ isomer, the propagation was continued for 80 ps. These trajectories were multiplied then by the cage symmetries and combined to evaluate the spatial distribution of O and Dy atoms at a given unit volume inside the fullerene with discretization of 0.042 × 0.042 × 0.042 Å^3^. The probability isosurfaces obtained by this approach for three isomers of Dy_2_O@C_82_ are plotted in **Figure** **4**. BOMD simulations also allow calculation of IR spectra via Fourier transformation of the time evolution of the dipole moment. Good agreement obtained between experimental and calculated spectra of three isomers (Figure S57, Supporting Information) confirms reliability of the calculated molecular dynamics. Importantly, the simulations predict a considerable variation of the spectral pattern with the isomeric structure of the fullerene cage, which agrees well with experimental observations.

For Dy_2_O@*C*_s_(6)‐C_82_ isomer, DFT calculations favor one particular conformer, which is more stable than all other six structures by 20–40 kJ mol^−1^. This conformer coincides with the major Dy1A–O1–Dy2A site resolved by X‐ray diffraction in Dy_2_O@*C*_s_(6)‐C_82_∙Ni(OEP)∙2(C_6_H_6_) (Figure 2a). The cluster is located on the symmetry plane of the cage. In reasonable agreement with diffraction data, DFT‐optimized Dy−O bond lengths are 2.031 and 2.045 Å, and Dy—O—Dy angle is 135.7°. Very similar positions of metal atoms with respect to the fullerene cage were found in the main crystallographic sites of Dy_2_S@*C*_s_(6)‐C_82_,[qv: 4c] Sc_2_S@*C*_s_(6)‐C_82_,^28^ Tb_2_C_2_@*C*_s_(6)‐C_82_,^29^ Lu_2_@*C*_s_(6)‐C_82_,[qv: 36c] Tm_2_@*C*_s_(6)‐C_82_,[qv: 33b] Tm_2_C_2_@*C*_s_(6)‐C_82_,[qv: 33b] and Er_2_@*C*_s_(6)‐C_82_.[qv: 36b] The BOMD trajectory started from the optimized structure of this conformer shows oscillation of the cluster near the starting position for at least 80 ps without rearrangement of the cluster. This dynamical behavior resembles that in Y_2_S@C_82_ with *C*_s_ cage symmetry reported by some of us recently.[qv: 4c] Thus, both experiment and theory agree in that the *C*_s_(6)‐C_82_ cage has two well‐defined positions for endohedral metal atoms common for different types of clusterfullerenes and dimetallofullerenes and that metal atoms tend to reside in these positions without extensive motion of the cluster. From two minor sites of the Dy_2_O cluster in Dy_2_O@*C*_s_(6)‐C_82_∙Ni(OEP)∙2(C_6_H_6_), the one with the occupancy of 0.15 is close to the third most stable DFT‐optimized conformer with the relative energy of 24 kJ mol^−1^ (Figure S54, Supporting Information). At this time we could not locate energy minimum close to the structure of the third minor site.

For Dy_2_O@*C*_3v_(8)‐C_82_ our calculations located five unique conformers with three of them lying within 1 kJ mol^−1^, and two others being at ≈10 kJ mol^−1^ higher (Figure S51 and Table S5, Supporting Information). *C*_3v_ point group further multiplies the Dy_2_O sites by symmetry operations, resulting in the dense filling of the space inside the fullerene with multiple quasienergetic positions of the cluster. The BOMD simulations reveal that the cluster easily rearranges itself between different minima, forming the egg‐shaped probability isosurface closely following the shape of the fullerene cage (Figure 4). The only positions avoided by metal atoms are located in front of pyrene fragments. These results agree well with the previous studies of internal dynamics in Y_2_S@C_82_,[qv: 4c] Sc_2_S@C_82_,[qv: 4a] and Sc_2_O@C_82_[qv: 15a] clusterfullerenes as well as dynamics of the Y_2_ dimer inside the *C*_3v_(8)‐C_82_ cage.^41^ ^13^C NMR spectroscopy also suggests free rotation of the endohedral cluster in M_2_C_2_@*C*_3v_(8)‐C_82_ and M_2_@*C*_3v_(8)‐C_82_ (M = Sc, Y, Lu)^41^, ^42^ since the cage carbon signals are averaged to give an apparent *C*_3v_ symmetry of the fullerene.

Thus, it comes as no surprise that the metal positions in the Dy_2_O@*C*_3v_(8)‐C_82_∙Ni(OEP)∙1.5(C_6_H_6_)∙CS_2_ crystal are disordered. In fact, the relative location of Dy sites and the shapes of thermal ellipsoids indicate that the Dy_2_O cluster is probably moving continuously, so that Dy atoms are “delocalized” in some regions inside the fullerene. The group of Dy sites (Dy1, Dy3, Dy5) with combined occupancy of 1.0 corresponds to one such region, whereas the sites (Dy2, Dy4, Dy6, Dy7, Dy8) demark moving areal of another Dy atom. Comparison with DFT‐optimized conformers shows that there is indeed a clustering of Dy positions of the two most stable conformers and one conformer with the energy of 9 kJ mol^−1^ in each of these regions (see Figure S55 in the Supporting Information for more details). The DFT‐optimized Dy—O bonds length and Dy—O—Dy angles in the two most stable conformers of Dy_2_O@*C*_3v_(8)‐C_82_ are 2.017 Å/2.044 Å/139° and 2.014 Å/2.035 Å/145°.

The conformer search for Dy_2_O@*C*_2v_(9)‐C_82_ located eight unique structures (Figure S53, Supporting Information), two of which are almost isoenergetic within 2 kJ mol^−1^, five structures have close relative energies in the range of 11–16 kJ mol^−1^, and one is rather unstable with Δ*E* at 63 kJ mol^−1^. The cluster dynamics in the *C*_2v_(9)‐C_82_ isomer presents an intermediate situation between localized motions as in *C*_s_(6)‐C_82_ and almost completely delocalized trajectory as in *C*_3v_(8)‐C_82_: BOMD simulations reveal that the regions of high probability density are more extended than in the *C*_s_ isomer but are not as delocalized as in *C*_3v_. Comparison to the diffraction data allows assignment of the real energy minima to sites Dy1–O–Dy2 and Dy1–O–Dy3, as well as their symmetry equivalents Dy1'–O–Dy2 and Dy1'–O–Dy3. The combined occupancy of these sites is 0.72. There is no theoretical minimum close the experimental Dy1–O–Dy2 cluster configuration. Remarkably, the relative energies of the conformers corresponding to the sites Dy1–O–Dy2' and Dy1–O–Dy3 are 14 and 16 kJ mol^−1^, respectively, and their Dy—O bonds length and Dy—O—Dy angles are 2.004 Å/2.040 Å/155° and 2.010 Å/2.047 Å/149°. Thus, unlike in *C*_s_(6) and *C*_3v_(8) isomers, the major experimental sites of Dy atoms in Dy_2_O@*C*_2v_(9)‐C_82_∙Ni(OEP)∙C_6_H_6_ do not correspond to the lowest energy conformers of the free Dy_2_O@*C*_2v_(9)‐C_82_ molecule.

To summarize, although there is an agreement between the DFT‐predicted conformers and experimentally determined positions of metal atoms inside the fullerene cages, it is also clear that intermolecular interactions in the crystals noticeably affects distribution of the Dy_2_O cluster positions inside the fullerene cages. For the C*_3_*_v_ isomer, this leads to the presence of a limited number of cluster positions from the multitude that might be expected based on the close energies of several conformers and a high symmetry of the cage. For the *C*_2v_ isomer, the observed conformations are not the most stable ones predicted theoretically. Besides, the application of the symmetry operations of the fullerene cage to the cluster positions should also give much more equivalent cluster sites than found experimentally. Our recent computational study of the interaction of M_3_N@C_80_ clusterfullerenes with Ni(OEP) in molecular complexes and crystals demonstrates that the intermolecular interactions result in the spread of the relative energies of the formally equivalent conformers in the range up to 20 kJ mol^−1^.^37^ That study also showed that the relative orientations of the EMF and Ni(OEP) molecules to a large extent are determined by the electrostatic interactions. In due turn, a spatial distribution of the electrostatic potential (ESP) around the EMF molecule depends strongly on the position of endohedral metal atoms.

Thus, the packing of the fullerene cages and the realization of certain cluster conformers in the crystals is a result of a subtle balance between the conformer stability and the cage‐Ni(OEP) interactions. When fullerene cage provides different conformations for the position of the endohedral cluster, the cluster can adjust its orientation during crystallization to minimize the energy for a given EMFs‐Ni(OEP) orientation. Furthermore, the minimization of the total crystal energy can be achieved for higher‐energy conformers of free EMF molecules, when they provide a more favorable spatial distribution of the electrostatic potential and optimal intermolecular interactions. Besides, if the EMF molecule has equally stable conformers with different location of metal atoms, a large variety of possible fullerene‐Ni(OEP) orientations may be also realized in different crystals as found for the EMFs with *C*_2v_(9)‐C_82_ cages.

The ratio of the total number of reported crystal structures to the number of orientation types can be used as a criterion of the structural diversity. With the ratio of 15:7, the *C*_2v_(9) isomer is obviously the most polymorphic one. On the other side is the *C*_s_(6) isomer with the ratio of 17:4. In fact, the vast majority of the crystal structures of EMFs with the C_82_‐*C*_s_(6) cage belong to only two orientation types: one is typical for EMFs with two endohedral metal atoms, whereas another type is found for EMFs with one metal (see the Supporting Information for more details). Thus, we conclude that the enhanced stability of one particular conformer in the isolated EMF molecule also results in the reduced variety of the crystal structures realized for this EMF, whereas the conformational flexibility also leads to a structural diversity.

### 2.4 Electronic Properties of Dy_2_O@C_82_ Isomers

Electronic properties of Dy_2_O@C_82_ isomers were studied by means of vis–NIR absorption spectroscopy and electrochemistry (cyclic voltammetry). Full details of these studies are presented in Supporting Information (Figures S58 and S59; Table S7, Supporting Information). In brief, absorption features of the studied compounds are similar to those of other metallofullerenes with C_82_ cages in a formal 4– charge state. The overall redox behavior of the three isomers of Dy_2_O@C_82_ varies from cage to cage and show noticeable deviation in redox potential from Sc_2_O@C_82_ analogs. DFT calculations show that frontier orbitals of all three isomers are localized on the fullerene with negligible contribution from the Dy_2_O cluster (Figure S59b, Supporting Information).

### 2.5 Magnetic Properties of Dy_2_O@C_82_ Isomers

#### 2.5.1 SQUID Magnetometry

**Figure 5:**
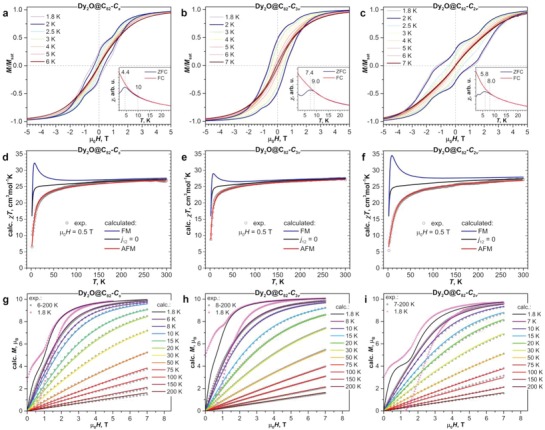
Magnetic hysteresis of Dy_2_O@C_82_ isomers: a) *C*_s_, b) *C*_3v_, and c) *C*_2v_; magnetic field sweep rate 2.9 mT s^−1^, inset in each figure shows determination of the blocking temperature *T*_B_ (temperature sweep rate 5 K min^−1^). Magnetic susceptibility χ (defined as M/H) of Dy_2_O@C_82_ isomers measured in the field of 0.5 T: d) *C*_s_, e) *C*_3v_, and f) *C*_2v_, experimental data (dots) are compared to simulations (lines) for antiferromagnetic coupling (AFM), ferromagnetic coupling (FM), and for noncoupled system (*j*_12_ = 0). Experimental (dots) and simulated (lines) magnetization curves of Dy_2_O@C_82_ isomers at different temperatures above *T*_B_: g) *C*_s_, h) *C*_3v_, and i) *C*_2v_. Simulation parameters are: *j*_12_ = −0.052 cm^−1^ and α = 50° for *C*_s_ isomer; *j*_12_ = −0.048 cm^−1^ and α = 60° for *C*_3v_ isomer; *j*_12_ = −0.093 cm^−1^ and α = 52° for *C*_2v_ isomer; for comparison, the curves measured at 1.8 K (pink dots) are also shown (they exhibit magnetic hysteresis and hence cannot be directly compared to simulated data).

Magnetic properties of Dy_2_O@C_82_ were studied by DC SQUID magnetometry using a Quantum Design VSM MPMS3 magnetometer. All three isomers showed magnetic hysteresis at low temperatures (average sweep rate 2.9 mT s^−1^; **Figure** **5**). However, the different shapes and closing temperatures of the hysteresis curves point to the significant dependence of the magnetic properties on the fullerene cage isomery. The *C*_s_ isomer exhibits comparatively narrow hysteresis loop closing near 6 K. At 2 K, the coercive field *H*_c_ is 0.35 T, and the loop remains open in the range from −3 to 3 T. The hysteresis loop of the *C*_3v_ isomer is broader (*H*_c_ = 0.59 T at 2 K, the loop is open till ≈4 T) and is closing near 8 K. The *C*_2v_ isomer shows the broadest hysteresis at 2 K (*H*_c_ = 1.1 T, open till 7 T), which is observed up to 8 K. Hysteresis loops of all three compounds have inflections at 1–1.5 T, which are most pronounced for the *C*_2v_ isomer. The nature of these features is discussed further in the text.

Blocking temperatures of magnetization (*T*_B_) were determined by comparing magnetic susceptibility (χ, defined here as M/H) of the sample measured during cooling in the field of 0.2 T (FC) to the curve measured during in‐field warming of the sample preliminary cooled down in zero field (ZFC). χ_ZFC_ curves measured with a temperature sweep rate of 5 K min^−1^ have well defined maxima at 4.4 K (*C*_s_), 7.4 K (*C*_3v_), and 5.8 K (*C*_2v_). At the same time, χ_ZFC_ and χ_FC_ curves diverge up to somewhat higher temperatures, 10 K (*C*_s_), 9 (*C*_3v_), and 8 K (*C*_2v_). Again, the shape of the carbon cage has a strong influence on the SMM properties of the encapsulated Dy_2_O cluster (see **Table** **1** for a summary of the data).

**Table 1:** Blocking temperatures, coercive fields, nature of Dy∙∙∙Dy interaction, and exchange barrier parameters in dinuclear Dy‐clusterfullerene SMMs with different bridging units (O^2−^, S^2−^, C_2_^2−^, C^4−^, N^3−^)

	*T*_B_ [K]	*T*_B100_ [K]	*H*_c_(2 K) [T]	Dy∙∙∙Dy	α [°]	*j*_12_ [cm^−1^]	*U* ^eff^ [K]	τ_0_ [s]	Ref.
Dy_2_O@C_82_‐*C*_s_	4.4	2.8	0.35	AFM	50	−0.052	10.8 ± 0.1	2.1 ± 0.1	t.w.
Dy_2_O@C_82_‐*C*_3v_	7.4	5.9	0.59	AFM	60	−0.048	7.7 ± 0.3	165 ± 27	t.w.
Dy_2_O@C_82_‐*C*_2v_	5.8	3.7	1.10	AFM	52	−0.093	18.6 ± 0.2	0.63 ± 0.06	t.w.
Dy_2_S@C_82_‐*C*_s_	≈2^a)^	–	0.02	FM	78	0.220	15.2 ± 0.3	0.003	[qv: 4c]
Dy_2_S@C_82_‐*C*_3v_	4.0	2.0	0.20	FM	79	0.18	6.5 ± 0.5	3.6 ± 0.8	[qv: 4c]
Dy_2_C_2_@C_82_‐*C*_s_	≈1.8^a)^	–	0.01	FM		0.175	17.4 ± 0.2	0.0005	[qv: 4c]
Dy_2_TiC@C_80_‐*I_h_*	≈2^a)^	1.7	0.08	FM	62	–	–	–	[qv: 7b]
Dy_2_ScN@C_80_‐*I_h_*	8.0	5.0	0.70	FM	63	0.073	10.7 ± 0.3	11.9 ± 1.5	[qv: 19c]
Dy_2_ScN@C_80_‐*D*_5_ *_h_*	5.3	2.6	0.48	FM	–	–	8.4 ± 0.2	4.1 ± 0.3	[qv: 22a]

^a)^ Approximate value since hysteresis is observed only near 2 K, where χ_ZFC_ cannot be measured reliably.

To get the information on the relaxation of magnetization in Dy_2_O@C_82_ isomers, their relaxation times (τ) were measured by DC magnetometry. The magnetization of the samples was first saturated in the field of 7 T, then the field was scanned to zero field (or to a certain finite field) with the highest possible sweep rate of 70 mT s^−1^ allowed by the magnetometer, and then the decay of magnetization was recorded while the sample was slowly restoring its equilibrium magnetization. The decay curves were then fitted with a stretched exponential function to determine an average relaxation time (single exponential function poorly describes the decay curves).

**Figure 6:**
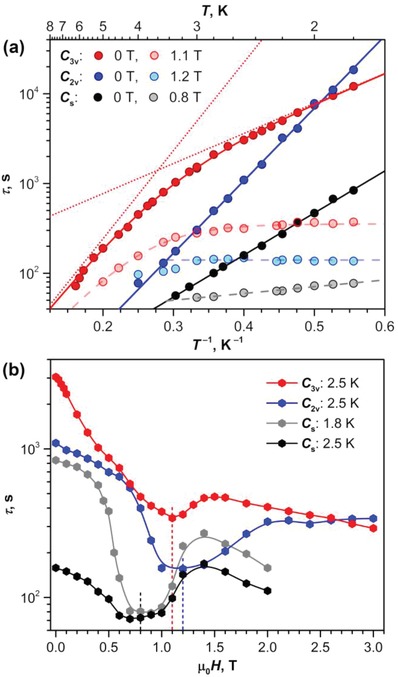
Magnetization relaxation times of Dy_2_O@C_82_ isomers: a) Temperature dependence in zero field (full red, blue, and black dots) and in the field, corresponding to the AFM‐FM level crossing for each isomer (pale dots); solid lines are fitting of zero‐field dependencies with one (black for *C*_s_, blue for *C*_2v_) or two Arrhenius processes (red for *C*_3v_), red dotted lines are contribution of individual processes for the *C*_3v_ isomer; dashed lines are fitting for in‐field relaxation times. b) Magnetic field dependence at the temperatures of 2.5 K (all isomers) and 1.8 K (*C*_s_ isomer); dashed vertical lines denote the fields, at which temperature dependencies shown in (a) were studied for each isomer.

Relaxation times measured in zero field are plotted in **Figure** **6**a in Arrhenius coordinates (log(τ)‐vs‐*T* ^−1^). A significant variation of the properties in dependence on the fullerene cage isomer can be well seen again. *C*_s_ and *C*_2v_ isomers show exponential decay of relaxation time with temperature (straight line in log(τ)‐vs‐*T* ^−1^ coordinates) in the whole accessible temperature range. Such a temperature dependence of relaxation time is usually associated with an Orbach relaxation mechanism

(1) $$\tau^{-1}(T)\;=\;\tau_{0}^{-1}\exp(-U^{\mathrm{eff}}/T)$$

where *U* ^eff^ is the energy barrier corresponding to the excited spin state involved in the spin reversal, and τ_0_ is the attempt time. Fit of the experimental data with Equation (1) gives *U* ^eff^ = 10.8 ± 0.1 K and τ_0_ = 2.1 ± 0.1 s for the *C*_s_ isomer and *U* ^eff^ = 18.6 ± 0.2 K and τ_0_ = 0.63 ± 0.06 s for the *C*_2v_ isomer. More complex temperature dependence is found for the *C*_3v_ isomer. Its relaxation times can be best fitted by a combination of two linear processes with *U* ^eff^/τ_0_ parameters of 7.7 ± 0.3 K/165 ± 27 s (prevails at the lowest temperatures) and 22.6 ± 0.8 K/2.63 ± 0.35 s. An onset of another relaxation mechanism can be also seen near 6 K, but shorter relaxation times cannot be measured reliably with DC magnetometry. Alternatively, a temperature dependence of the relaxation times of the *C*_3v_ isomer can be also described by a power function of temperature, $\tau_{}^{-1}(T)\;=\;CT^{n}$, with parameters *C* = (7.1 ± 0.2) × 10^−6^ s^−1^ K^−^ *^n^* and *n* = 4.12 ± 0.03.

The attempt times of the linear processes are much longer than normally observed for the Orbach mechanism involving a ligand‐field excited state of a lanthanide ion. Besides, the ligand field splitting for Dy ions in Dy_2_O@C_82_ isomers is hundreds of K (see discussion below). Hence, the Orbach relaxation via single‐ion LF‐excited states of Dy can be excluded as an explanation of the Arrhenius behavior at low temperatures. Instead, one has to consider the spin states formed by the intramolecular interaction of two Dy ions as had been already found in Dy_2_ScN and Dy_2_S clusterfullerenes.[qv: 4c,19c,22b]

#### 2.5.2 Ab Initio Calculations

**Figure 7:**
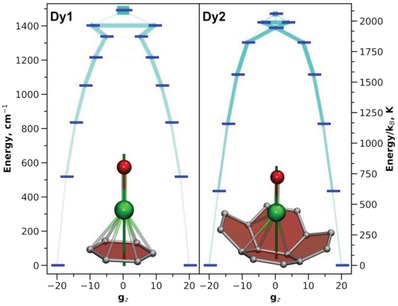
Ab initio computed ligand‐field states (thick blue dashes) and transition probabilities between them (light blue lines; the thicker the line, the higher the transition probability) for two Dy ions in the lowest‐energy conformer of Dy_2_O@C_82_‐*C*_s_. Also shown are Dy‐cage coordination sites and quantization axes for each Dy ions (dark green lines). Dy, green; O, red; C, gray; Dy—C distances less than 2.60 Å are shown as bonds.

Ab initio calculations were performed to get information on the single‐ion magnetic anisotropy of Dy ions in Dy_2_O@C_82_. For each Dy_2_O@C_82_ structure, one of the Dy ions was replaced by Y, and then the multiplet structure for Dy (^6^H_15/2_) in DyYO@C_82_ was calculated at the CASSCF(9,7)/SO‐RASSI level using SINGLEANISO routine to extract ab initio ligand‐field parameters.^43^ Calculations for different DFT‐optimized conformers of Dy_2_O@C_82_ isomers gave similar results. **Figure** **7** visualizes computed ligand‐field (LF) splitting, transition probabilities, and Dy‐cage coordination sites with quantization axes of Dy ions for Dy_2_O@C_82_‐*C*_s_ (major conformer). Analogous data for different conformers of other Dy_2_O@C_82_ isomers are summarized in the Supporting Information (Figures S60–S73, Tables S8–S21). The oxide ion at the distance of ≈2 Å from Dy^3+^ imposes a very strong uniaxial anisotropy in the latter. The quantization axes for Dy ions are almost coinciding with corresponding Dy—O bonds, and the overall LF splitting is in the range of 1360–1490 cm^−1^. The energy of the first excited Kramers doublet (KD) varies from 386 to 515 cm^−1^. In terms of the | *J*, *m_J_*〉 basis, the first KDs have nearly pure composition (near 100% | ±15/2〉 for the first KD, ≈98% | ±13/2〉 for the second KD, ≈90% | ±11/2〉 in the third KD etc.), and substantial mixing of *m_J_* functions starts from the fifth or sixth KD. Accordingly, transition probabilities within one KD attain significant values only in the fifth‐sixth KDs, which shows that the Orbach relaxation via the ligand‐field excited states should have a barrier of at least 1200–1300 cm^−1^ (≈1700–1900 K). Similar findings were reported by Singh and Rajaraman [qv: 19e] in a computational study of DyLuO@C_2n_ and DyScO@C_2n_ (2*n* = 72, 76, 82) molecules. Unfortunately, the isolable amount of Dy_2_O@C_82_ is not sufficient for its study by AC magnetometry, which is required for the measurement of relaxation times above *T*_B_. At the temperatures accessible for DC magnetometry, the relaxation of magnetization in Dy_2_O@C_82_ is not governed by excited ligand‐field states.

Although the overall anisotropy patterns for Dy ion in all Dy_2_O@C_82_ structures we studied are similar, a variation of the LF energies by 100–200 cm^−1^ should be pointed out. There seems to be no difference between cage isomers in this regard, i.e., the changes between different conformers are comparable for each cage isomer. The LF parameters may be influenced by the variation of geometrical parameters of the Dy_2_O cluster. However, we could not find any correlation between Dy—O distances and the energy of the first excited KD or the total LF splitting (Figures S74 and S75, Supporting Information). Most likely, the differences are caused by the fullerene cage, and in particular by the arrangement of carbon atoms at the Dy coordination sites.

Analysis of more than twenty Dy‐cage coordination sites in DFT‐optimized structures (Figures S60–S73, Supporting Information) revealed three main motifs. In some conformers, Dy is located above the center of a cage hexagon and exhibits η^6^ coordination. More often, Dy is displaced from the position above the center of a hexagon toward one of the pentagon/hexagon edges. In this situation, coordination motif changes to η^4^ or even η^2^. In the third coordination type, Dy is located close to the pentagon/hexagon/hexagon (acenaphthylene) moiety and exhibits η^2^ coordination to the hexagon/hexagon edge (but Dy—C distances to two carbon atoms of the pentagon are also rather short). In general, there is a quasicontinuum of Dy‐cage coordination geometries as the outlined motifs are interconvertible via small displacements of Dy ions. At this moment, we could not find any obvious correlation between Dy‐fullerene coordination site and variation of the LF splitting.

The highest LF splitting is realized for a high‐energy conformer of the *C*_2v_ isomer (Table S21, Figure S73, Supporting Information). Here Dy has a unique η^4^ coordination to a pentagon, in which metal atom is not exactly above the center of the latter, but is close to one of the 5/6 edges. This type of metal‐cage coordination imposes a stronger influence on the spin properties of Dy ion as its quantization axis is displaced noticeably from the Dy−O bond. Interestingly, the η^5^ coordination of Dy to cyclopentadienyl ring is known to produce the largest LF splitting in Dy‐SMMs, including Dy‐metallocenes with the highest blocking temperatures of magnetization among SMMs.^44^ Realization of such coordination in EMFs may also lead to unprecedented magnetic properties but is not described experimentally yet.

#### 2.5.3 Coupling of Dy Magnetic Moments in Dy_2_O@C_82_

**Figure 8:**
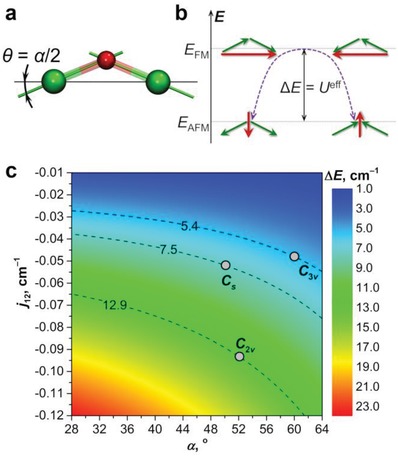
a) Ab initio computed single‐ion quantization axes (green lines) in Dy_2_O@C_82_‐*C*_s_ (carbon cage not shown, O is red, Dy is green) and definition of the angles θ and α (note also that the angle between magnetic axes of Dy ions α is approximately equal to π − ∠Dy—O—Dy). b) Schematic description of antiferromagnetically (AFM) and ferromagnetically (FM) coupled states of Dy_2_O@C_82_ (magnetic moments of individual Dy ions and the resulting moment of the whole Dy_2_O cluster are shown as green and red arrows, respectively). c) Energy difference between AFM and FM states (Δ*E*) as a function of the coupling constant *j*_12_ and the angle α. Isolines correspond to *U* ^eff^ values determined from the temperature dependence of relaxation times in zero field. Dots correspond to the pairs of *j*_12_/α parameters giving the best match to the experimental magnetization data as shown in Figure 5.

According to ab initio calculations, magnetic ground state for each Dy ion in Dy_2_O@C_82_ clusterfullerene is a Kramers doublet with essentially pure *m_J_* = ±15/2 composition and magnetic moment aligned along the Dy–O bond (**Figure** **8**a). For two noninteracting Dy ions in zero magnetic field, the ground magnetic state would be a quartet. But Dy∙∙∙Dy magnetic interactions split the four spin states of Dy_2_O into two quasidoublets schematically shown in Figure 8b. In one doublet, magnetic moments of Dy ions are oriented in the same direction along the Dy–Dy axis (defined as *z*‐axis hereafter). The total magnetic moment is then aligned along the *z* axis. In the other doublet, the moments have opposite orientation along the *z* axis, but inevitably have the same orientation in the perpendicular direction (defined as y‐axis hereafter). The total magnetic moment is then aligned along the *y*‐axis. In the following, the states with the moment along the *z*‐axis will be defined as ferromagnetic (FM), whereas the states with the moment along the *y*‐axis are defined as antiferromagnetic (AFM) since magnetic moment of the FM state is larger than that of the AFM state as long as the ∠Dy—O—Dy angle exceeds π/2.

The low‐energy spin states of Dy_2_O@C_82_ can be reasonably well described by the following spin Hamiltonian

(2) $${\hat{H}}_{\mathrm{spin}}\;=\;{\hat{H}}_{{\mathrm{LF}}_{1}}\;+\;{\hat{H}}_{{\mathrm{LF}}_{2}}\;-\;2j_{12}\hat{J_{1}}\cdot \hat{J_{2}}\;+\;{\hat{H}}_{\mathrm{ZEE}}$$

where ${\hat{H}}_{{\mathrm{LF}}_{i}}$ are single‐ion ligand‐field Hamiltonians (their parameters can be obtained from ab initio calculations), *j*_12_ is the coupling constant between the lanthanide moments, $\hat{J_{i}}$, and ${\hat{H}}_{\mathrm{ZEE}}$ describes Zeeman splitting. The energy difference between FM and AFM states can be then computed as

(3) $$\Delta E_{\mathrm{AFM}-\mathrm{FM}}\;=\;225j_{12}\cos\left(\alpha \right)$$

where α is the angle between quantization axes of Dy ions and is approximately equal (π−∠Dy—O—Dy), whereas the coefficient 225 appears because of the *m_J_* = ±15/2 ground state of Dy^3+^. The shape of the *χT* curves (Figure 5) points to the prevalence of the antiferromagnetic interactions between Dy ions, which means that *j*_12_ is negative. The Δ*E*_AFM−FM_ energy as a function of *j*_12_ and α is shown as 2D map in Figure 8c.

The assumption that the low‐temperature relaxation of magnetization in Dy_2_O@C_82_ proceeds via the FM excited state allows determination of the Δ*E*_AFM−FM_ energy difference as the *U* ^eff^ value from the temperature dependence of relaxation times. Furthermore, this assumption imposes a useful constraint on the possible pairs of (*j*_12_, α) parameters – they should be on the iso‐energy lines plotted in Figure 8c for the three isomers of Dy_2_O@C_82_. Using Hamiltonian Eq. (2) and PHI code^45^ to simulate magnetization curves at different temperatures with this constraint in mind, we found that the best match to the experimental data is obtained for the following *j*_12_/α values: −0.052 cm^−1^/50° for the *C*_s_ isomer, −0.048 cm^−1^/60° for the *C*_3v_ isomer, and −0.093 cm^−1^/52° for the *C*_2v_ isomer (Figure 5). These values also give a perfect match for the *χT* curves (which were not used in the optimization of parameters). Note that if the sign of *j*_12_ is reversed (favoring then the FM state), the *χT* curves develop a characteristic sharp peak at low temperatures. Such peaks are not observed experimentally, proving that the ground magnetic state of all three Dy_2_O@C_82_ isomers is indeed AFM. The optimal angles α should be understood as an average value since there is most probably a distribution of Dy—O—Dy angles in the real structures (see X‐ray data in Figure 2 and discussion of DFT results above). Variation of α within few degrees gives small changes in the magnetization curves, and hence the precision of at least ±2°–3° should be assumed. Finally, a good agreement between experimental and simulated magnetization and *χT* curves confirms the validity of the assumption that the *U* ^eff^ values correspond to Δ*E*_AFM−FM_ energy differences.

**Figure 9:**
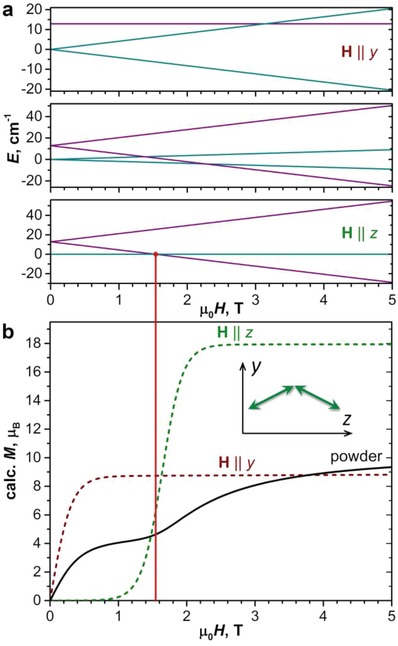
a) Low‐energy Zeeman diagrams of Dy_2_O@C_82_‐*C*_2v_ for different orientations of the magnetic field: field parallel to *y* axis (top), parallel to *z* axis (bottom), and arbitrary orientation. b) Simulated magnetization curves of Dy_2_O@C_82_‐*C*_2v_ at 1.8 K for different orientations of the magnetic field: parallel to *y* axis, parallel to *z* axis, and powder‐averaged; the inset shows orientation of the axes with respect to the magnetic moments of Dy ions, which are considered to be in the *yz* plane. Vertical red line shows correspondence between the level crossing in Zeeman diagram and an inflection in the magnetization curve.

The inflections in the low‐temperature magnetization curves of Dy_2_O@C_82_ can be explained by the splitting and variation of the energy of the FM and AFM states in the magnetic field. **Figure** **9** shows simulated Zeeman splitting and magnetization of the Dy_2_O@C_82_‐*C*_2v_ when the field is applied along *z*, *y*, and an arbitrary direction. When the field is applied along the *z*‐axis, the ground AFM state is not affected because its magnetic moment is oriented along the *y*‐axis. The FM state experiences strong Zeeman splitting, and at a field of 1.54 T one of the branches of the FM doublet crosses the energy of the AFM state. The magnetization remains zero before the field of 1 T as AFM remains the ground state, but then increases quickly to the saturation value of 10∙2cos(α/2) *µ*_B_ = 18.1 *µ*_B_ between 1 and 2 T as the FM state becomes lower in energy. If the field is applied along the *y*‐axis, the FM state remains intact, whereas the AFM state undergoes Zeeman splitting. The magnetization increases fast from zero to the saturation at 10∙2sin(α/2) *µ*_B_ = 8.5 *µ*_B_.

In the real powder sample, molecules are oriented at all possible angles with respect to the external field. However, the two regimes outlined above can be still distinguished in the powder‐averaged magnetization curve shown in Figure 9b. The crossing between AFM and FM‐dominated regimes is smeared because of the distribution of the molecular orientations but still can be observed as an inflection in the magnetization curve in the field range corresponding to the level crossing. This feature in the curve simulated for *T* = 1.8 K reproduces a similar inflection in the real experimental curve (Figure 5i), although the latter has a hysteresis and cannot be exactly compared to the simulations assuming thermodynamic equilibrium. At higher temperatures, when hysteresis is closed, the state populations are changing not so abruptly, and the inflection is not detectable any more.

#### 2.5.4 Quantum Tunneling of Magnetization in Dy_2_O@C_82_

Near the level crossing, a magnetic system may have an additional relaxation pathway caused by the quantum tunneling of magnetization (QTM). For instance, the QTM is usually the fastest relaxation process for single‐ion magnets in zero field. Dy∙∙∙Dy interactions in dinuclear systems quench the zero‐field QTM. But the QTM may be operational in the finite fields, at which the level crossing takes place. To analyze this possibility, we studied the field dependence of relaxation times. The dependencies were measured at 2.5 K to have reasonably but not extremely long relaxation times (accuracy of the measured times decrease as they become shorter than ≈100 s). For the *C*_s_ isomer exhibiting faster relaxation of magnetization, the field dependence was also studied at 1.8 K.

Figure 6b shows that in low fields, all Dy_2_O@C_82_ isomers exhibit a gradual decrease of the relaxation time with the increase of the field. This behavior can be attributed to the increasing contribution of the direct relaxation mechanism, in which the spin flip is caused by the phonon matching the Zeeman splitting. With further increase of the magnetic field, all isomers show an abrupt acceleration of the relaxation. For the *C*_s_ isomer the change of the relaxation mechanism starts already at 0.4 T, for the *C*_3v_ and *C*_2v_ isomers the changes become apparent at 0.7–0.8 T. At further increase of the field, relaxation times increase again and then turn to a gradual decay. The dips (negative peaks) in the τ‐*H* dependencies are ≈1 T broad and correspond to the inflections in the magnetization curves. We propose that they are caused by the QTM at the AFM‐FM level crossing. The large width of these “resonances” is caused by the distribution of the molecular orientations in the powder sample as well as by the distribution of dipolar fields.

To prove the QTM mechanism, we studied the temperature dependence of relaxation times at the fields close to the centers of the dips. The characteristic feature of the QTM is the temperature independence of the relaxation times (however, weak temperature dependence can be sometimes observed for the QTM and is presumably caused by the temperature dependence of the phonon collision rate^46^). Indeed, we found that the relaxation time measured in these fields fulfill this criterion (Figure 6a). For the *C*_3v_ and *C*_2v_ isomers, the QTM is dominant up to 3 K, whereas thermally activated processes start to contribute above that temperature. For *C*_3v_, we could fit the whole dependence by a combination of QTM with τ_QTM_ of 394 ± 6 s and an Orbach process with *U* ^eff^ of 19.3 ± 0.7 K and τ_0_ of 2.3 ± 0.4 s. Note that parameters of this Orbach process are similar to those observed for the second Orbach process in zero field. For the *C*_2v_ isomer, the τ_QTM_ is found to be 140 ± 5 s.

For the *C*_s_ isomer, the in‐field relaxation times gradually decrease from 77 s at 1.8. K to 53 s at 3 K. The temperature dependence can be fitted by the power function of temperature, $\tau_{}^{-1}(T)\;=\;CT^{n}$, with *C* = (8.4 ± 0.4) × 10^−3^ s^−1^ K^−^ *^n^* and *n* = 0.75 ± 0.06. Such a small exponent in the power function may correspond to the direct process (theoretical *n* = 1). Alternatively, this temperature dependence can be also described by an Orbach process with *U* ^eff^ = 1.7 ± 0.1 K and τ_0_ = 29.8 ± 1.5 s. But since the relaxation times of the *C*_s_ isomer are already rather short and therefore are not very precise, we prefer to restrain ourselves from a further discussion of the reason for the temperature dependence of the in‐field relaxation times in the *C*_s_ isomer. It is suffice to note that in‐field relaxation times of the *C*_s_ isomer are much faster than zero‐field values and that the temperature variation is substantially less pronounced than in zero field.

To summarize this section, we showed that all three isomers of Dy_2_O@C_82_ have AFM ground state and exhibit sufficiently slow relaxation of magnetization to develop a magnetic hysteresis. In zero field, relaxation of magnetization follows the Orbach mechanism involving the FM excited state, which allows precise determination of the AFM‐FM energy splitting. The latter, in due turn, simplifies determination of the coupling constant describing Dy∙∙∙Dy interaction and the average angle between magnetic moments of Dy ions. As the FM state shows stronger field dependence, this state becomes prevalent at high fields. At the fields corresponding to the AFM‐FM level crossing, all isomers exhibit QTM, which is apparent from the “dips” in the field dependence of the relaxation times as well as from the temperature‐independent in‐field relaxation times.

#### 2.5.5 Comparison to Other EMF‐SMMs

It is instructive to compare the magnetic properties of Dy_2_O@C_82_ to other SMMs based on Dy‐clusterfullerenes. Structurally, oxide clusterfullerenes are similar to sulfide clusterfullerenes. Both Dy_2_O and Dy_2_S species transfer four electrons to the fullerene cage and hence prefer the same cage isomers, and both clusters have an angular shape (but Dy—S—Dy angles are smaller than Dy—O—Dy angles). Earlier we isolated Dy_2_S@C_82_ with *C*_s_(6) and *C*_3v_(8) cage isomers and studied their SMM properties.[qv: 4c] *C*_s_ isomer of Dy_2_S@C_82_ was found to be a softer SMM than the *C*_3v_ isomer showing a similar effect of the fullerene cage isomer as found in this work for Dy_2_O@C_82_.

**Figure 10:**
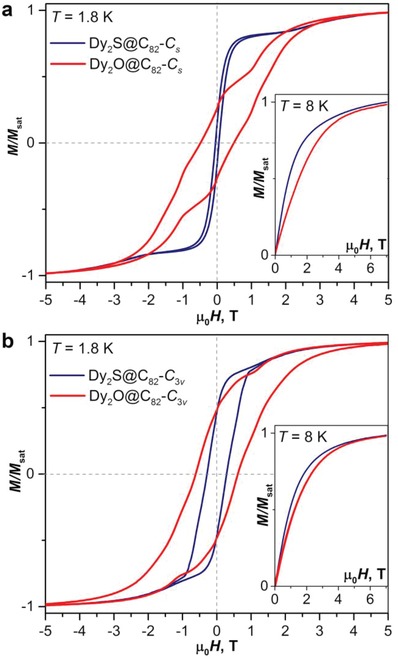
Comparison of magnetic hysteresis of Dy_2_S@C_82_ and Dy_2_O@C_82_ at 1.8 K: a) *C*_s_ isomers; b) *C*_3v_ isomers (average magnetic field sweep rate 2.9 mT s^−1^). The insets show magnetization curves measured at 8 K.

But in other aspects, magnetic properties of Dy_2_O@C_82_ are substantially different from those of Dy_2_S@C_82_. As can be seen from comparison of magnetic hysteresis curves of Dy_2_O@C_82_ and Dy_2_S@C_82_ with *C*_s_ and *C_3_*_v_ cage isomers shown in **Figure** **10**, oxide clusterfullerenes are considerably stronger SMMs than their sulfide analogs. They show broader hysteresis and much longer relaxation times (and hence higher blocking temperatures, see Table 1 for comparison of some SMM parameters). For instance, *T*_B_/*T*_B100_ temperatures of the *C*_3v_ isomer of Dy_2_S@C_82_ are 4.0/2.0 K, whereas for Dy_2_O@C_82_ they are increased to 7.4/5.9 K. Thus, replacement of S by O in otherwise similar structures results in a dramatic improvement of the SMM performance. The strength of Dy∙∙∙Dy exchange interactions in Dy_2_S cluster is similar to those in Dy_2_O but has an opposite sign (i.e., the FM state is the ground state). Furthermore, the Dy—S bonds of 2.4–2.5 Å are much longer than Dy−O bonds, and the LF in sulfide clusterfullerenes is almost twice smaller. Ab initio calculations predict that the overall LF splitting in Dy_2_S@C_82_ is 880–970 cm^−1^, whereas the energy of the first excited KD is 220–300 cm^−1^.

The size of the LF splitting in Dy_2_O@C_82_ is similar or even higher than in Dy_2_ScN@C_80_‐*I_h_*. So far, the latter has been the strongest SMM among the clusterfullerenes.[qv: 18a] Note that Dy_2_@C_80_(CH_2_Ph), which is the strongest SMM among all Dy‐EMFs, not considered as a clusterfullerene.[qv: 18b]*T*_B_ of Dy_2_O@C_82_‐C_3v_ studied in this work is close to that of Dy_2_ScN@C_80_, and the *T*_B100_ value of the oxide is even higher than for the nitride clusterfullerene. Apparently, similarity of the LF also results in comparable SMM properties, at least at low temperatures. Both types of EMF‐SMMs also exhibit similar spin‐relaxation mechanism at low temperature featuring the exchange‐excited state of the dinuclear cluster. But in the Dy_2_ScN cluster, as in the aforementioned Dy_2_S, the ground state is FM,[qv: 22b] whereas Dy_2_O favors AFM coupling.

For sulfide and nitride clusterfullerenes we also found a strong influence of the carbon cage on the low‐temperature (<10 K) relaxation behavior.[qv: 4c,22a] As insignificant variations of the LF are not relevant at these low temperatures, we hypothesized that the fullerene cage contributes to the relaxation of magnetization via the spin‐phonon coupling and the energy transfer from the spin system to the lattice. Free motion of the endohedral cluster indicates that the potential energy surface is flat, and that the cluster vibrations are weakly coupled to the fullerene modes. The spin‐lattice energy transfer is then not very efficient, i.e., the fullerene cage shields the endohedral cluster not only from chemically active environment but also from the lattice phonons. On the contrary, restricted motion of the endohedral cluster points to the stronger coupling to the cage vibrations, which facilitates the spin‐lattice relaxation. As a result, the EMFs with more “isotropic” symmetric cages (*I_h_* for Dy_2_ScN@C_80_ or *C*_3v_ for Dy_2_S@C_82_) are better SMMs than the analogous EMFs with less symmetric cages. The results of these work on three isomers of Dy_2_O@C_82_ perfectly follow this trend. The *C*_3v_ isomer with the most pronounced motions of the Dy_2_O cluster (Figure 4) is the best SMM in the series, the *C*_s_ isomer with the fixed cluster is the weakest SMM, whereas the *C*_2v_ isomer is in between. Why the exchange interactions in Dy_2_O@C_82_ are so much dependent on the fullerene cage isomer and why the coupling in Dy_2_O is antiferromagnetic whereas in other dinuclear Dy‐EMFs the coupling is ferromagnetic, remain open questions.

#### 2.5.6 Comparison to Nonfullerene SMMs

Dy–O bonding is frequently used in the first coordination sphere of the lanthanide ion in the design of SMMs. There are at least two ways, how Dy—O bonds can affect the SMM properties: (i) by affecting magnetic anisotropy of oxygen‐bonded Dy ions and (ii) by mediating Dy∙∙∙Dy exchange interactions via µ_2_‐O bridge. In the following we show that in Dy_2_O@C_82_ clusterfullerenes both factors are reaching their limiting values.

*LF Splitting*: When a Dy—O bond is short and oxygen bears a considerable negative charge, a strong LF splitting evolves for the Dy ion. In the computational study, Ungur and Chibotaru predicted^47^ that a hypothetical diatomic complex [DyO]^+^ would have a very short Dy−O bond length of 1.74 Å and a large spin relaxation barrier exceeding 2100 cm^−1^. Whereas [DyO]^+^ cannot be obtained as an isolated entity, the closest experimental realization of such unit was reported by Kazin et al. via partial substitution of Ca or Sr in corresponding apatite lattices,^48^ which gives semi‐isolated DyO^+^ fragments with the bond length of 2.05–2.14 Å. The solids then show Dy‐based magnetic hysteresis with blocking temperatures up to 11 K and thermal barriers of relaxation of 780–1040 cm^−1^ assigned to the 4th or 5th Kramers doublets.

Among the molecular compounds (see **Table** **2** for selected examples), several strongest SMMs have the pentagonal bipyramidal coordination of Dy, in which short Dy−O bonds are located axially, whereas equatorial ligands are comparably weak. The strongest SMM from such series is the [Dy(O^t^Bu)_2_(py)_5_][BPh_4_] salt with *T*_B_ of 14 K and the relaxation barrier of 1261 cm^−1^, corresponding to the 5th‐8th Kramers doublets. The average axial Dy−O bond length in this molecule is 2.111(2) Å.^49^

**Table 2:** Selected SMMs with short Dy—O bonds and their structural and SMM parameters

Compound^a)^	Coordination	Dy—O [Å]	*T*_B_ [K]	exp. *U* ^eff^ [cm^−1^]	calc. *U* ^eff^ [cm^−1^]	KD	Ref.
[DyO]^+^	linear	1.74	–	–	2100	5–7	47
Ca_9.5_Dy_0.5_(PO_4_)_6_(OH_0.75_)_2_	PBP^b)^	≈2.05	4.5	792(19)		4	48
Sr_9.9_Dy_0.1_(PO_4_)_6_(OH_0.95_)_2_	PBP^b)^	≈2.05	11	1025(15)		5	48
[Dy(O^t^Bu)_2_(py)_5_][BPh_4_]	PBP^b)^	2.112(2)	14	1261(1)	≈1200	5–8	49
[Dy_5_O(O*^i^*Pr)_13_]	Distorted octahedron	1.95(2)	–	559^c)^	685	3	32, 50
[Dy_4_K_2_O(O^t^Bu)_12_]	Distorted octahedron	2.07(1)	–	585^c)^	617	3	50
[L_2_Dy(H_2_O)_5_][I]_3_ ∙ L_2_ ∙ (H_2_O)^d)^	PBP^b)^	2.208(2) 2.203(2)	12	452/511	478	3	51
[Dy(µ‐OH)(DBP)_2_(THF)]_2_ ^e)^	Distorted square pyramid	2.094(2) 2.120(2)	≈7–8	501	530	3	52
[Dy(Cy_3_PO)_2_(H_2_O)_5_]^3+^	PBP^b)^	2.189(3) 2.210(3)	11	377	250	2	53
[Dy(bbpen)Br]^f)^	PBP^b)^	2.163(3)	9.5	712	721	4	54
Dy_2_O@*C*_s_‐C_82_	quasilinear	1.985(11)	4.4	–	≈1200	5–7	t.w.
Dy_2_O@*C*_3v_‐C_82_	quasilinear	1.978(9)	7.4	–	≈1200	5–7	t.w.
Dy_2_O@*C*_2v_‐C_82_	quasilinear	1.944(8)	5.8	–	≈1200	5–7	t.w.

^a)^ Dy—O distances are mainly from X‐ray structures; for compounds with multiple Dy—O bonds only the shortest values are listed, but for Dy_2_O@C_82_ we list the average Dy—O bond length; *T*_B_ is the blocking temperature from FC/ZFC measurements; “exp. *U* ^eff^” is the experimental relaxation barrier via LF excited states; “calc. *U* ^eff^” is the relaxation barrier via LF excited states predicted ab initio by CASSCF calculation; KD gives the number of the Kramers doublet which offers efficient relaxation

^b)^ PBP, pentagonal bipyramid

^c)^ In the samples strongly diluted with Y

^d)^ L = (^t^BuPO(NH^i^Pr)_2_)

^e)^ DBP–,2,6‐di‐tert‐butylphenolate

^f)^ H_2_bbpen, *N*,*N*′‐bis(2‐hydroxybenzyl)‐*N*,*N*′‐bis(2‐methylpyridyl)ethylenediamine.

As already discussed above, Dy—O bonds of ≈2.0 Å in Dy_2_O@C_82_ are among the shortest Dy—O bonds in molecular compounds. Besides, the oxygen has quite a large negative charge because of the ionic Dy−O interactions. Ab initio analysis shows that the LF splitting in Dy_2_O@C_82_ is the highest among all experimentally available SMMs with Dy—O bonds, and the relaxation of magnetization is predicted to proceed via the 6th‐7th Kramer doublets with the energies exceeding 1200–1300 cm^−1^ (Figure 7 and the Supporting Information). In the low‐temperature range accessible in this work, the relaxation of magnetization in Dy_2_O@C_82_ does not follow the single‐ion LF states, and hence the large size of the thermal barrier for this mechanism cannot be proven yet. However, similarly high ab initio predictions of the Orbach barrier in Dy_2_ScN@C_80_ were confirmed by AC magnetometry, which revealed the thermal barrier of 1206 ± 16 cm^−1^ (1735 ± 21 K) in the temperature range of 60–76 K.[qv: 19c] Importantly, large LF splitting in Dy_2_O@C_82_ ensures that Dy ions remain in their ground magnetic state and that there are no efficient relaxation channels via LF excited state.

*Exchange Interactions*: Bridging oxo‐ligand is a ubiquitous element in the structure of polynuclear SMMs. Dozens of di‐nuclear Dy SMMs and a number of SMMs with higher nuclearity containing {Dy_2_O_2_} bridges have been reported,^55^ and exchange interactions between Dy atoms in such bridges were a matter of detailed studies. A comparison of the Dy∙∙∙Dy interactions in Dy_2_O@C_82_ with one Dy—(µ_2_‐O)—Dy exchange channel to the exchange parameters in other SMMs with {Dy_2_O_2_} bridges, which have two such channels, is of high interest (**Table** **3**).

**Table 3:** Geometry parameters and Dy∙∙∙Dy interactions in Dy_2_O@C_82_ isomers and selected {Dy_2_O_2_} molecular magnets

	µ_2_‐O type	Dy—O [Å]	Dy∙∙∙Dy [Å]	∠Dy—O—Dy [°]	Δ*E*_AFM−FM_ [cm^−1^]	*J*_tot_ [cm^−1^]	*J*_dip_ [cm^−1^]	*J*_exch_ [cm^−1^]	Ref.
**{Dy_2_O}**									
Dy_2_O@C_82_‐*C*_s_	O^2–^	1.985(11)^a)^	3.754(2)	130^b)^	−7.5	−23.3	9.3	−32.6	t.w.
Dy_2_O@C_82_‐*C*_3v_	O^2–^	1.978(9)^a)^	≈3.9^c)^	120^b)^	−5.4	−21.6	10.2	−31.8	t.w.
Dy_2_O@C_82_‐*C*_2v_	O^2–^	1.944(8)^a)^	≈3.9^c)^	128^b)^	−12.9	−41.9	8.6	−50.5	t.w.
**{Dy_2_O_2_}** ^d)^									
**A**	enol	2.352(6) 2.355(5)	3.990(1)	115.9(2)	6.0	11.39	4.64	6.75	57
**B**	enol	2.397(4) 2.313(4)	3.677(1)	102.6(1)	−5.3	−11.03	−2.65	−8.38	58
**C**	enol	2.330(7) 2.437(7)	4.015(1)	114.7(3)	4.5	9.69	4.69	5.00	59
**D**	oxime	2.319(4) 2.320(4)	3.838(5)	111.7(1)	−4.7	−9.2	−2.4	−6.8	60
**E**	enol	2.332(1) 2.307(1) 2.296(1) 2.334(1)	3.781(2)	109.6(1) 109.1(1)	−3.5	−6.77	−2.40	−4.37	61
**F**	enol	2.376(3) 2.332(3)	3.735(1)	107.7(1)	3.0	5.93	6.18	−0.25	62
**G**	enol	2.266(5) 2.324(3)	3.809(1)	113.4(1)	−2.9	−6.05	−2.05	−4.00	63
**H**	enol	2.287(9) 2.368(1)	3.700(1)	105.3(1)	−2.9	−5.87	−3.12	−2.75	64
**I**	enol	2.323(4) 2.333(4) 2.335(4) 2.340(4)	3.864(1)	112.2(2) 111.5(2)	2.9	5.88	5.36	0.52	[qv: 55d]
**J**	hydroxyl enol	2.263(2) 2.300(2) 2.511(2) 2.482(2)	3.878(5)	116.4(1) 101.9(1)	2.8	6.20	4.20	2.00	65
**K**	enol	2.382(6) 2.328(6)	3.938(1)	113.4(1)	2.7	6.31	4.56	1.75	66
**L**	enol	2.318(5) 2.336(5) 2.337(5) 2.304(5)	3.840(1)	111.1(1) 111.7(2)	2.6	5.09	5.84	−0.75	62
**M**	alkoxide	2.245(2)	3.728(1)	119.1(1)	−2.5	−5.41	0.09	−5.50	67
**N**	alkoxide	2.231(2)	3.707(1)	110.7(1)	2.5	5.04	5.79	−0.75	68
**O**	enol	2.347(4) 2.341(4)	3.942(1)	114.5(2)	2.3	4.98	5.01	−0.03	59
**P**	enol	2.270(3) 2.277(2)	3.851(1) 3.845(1)	113.0(1) 113.3(1)	−2.2	−4.49	−2.74	−1.75	69
**Q**	enol	2.324(3) 2.346(3)	3.919(1)	114.1(1)	2.1	4.47	5.22	−0.75	59
**R**	enol	2.312(2) 2.345(2)	3.900(1)	113.7(1)	2.0	3.82	4.82	−1.00	66
**S**	enol	2.348(3) 2.368(3)	3.755(1)	105.5(1)	0.3	−1.30	6.03	−7.33	70
**T**	enol	2.318(3) 2.323(4)	3.787(5)	109.3(1)	0.1	0.25	6.10	−5.85	63

^a)^ Average Dy—O distance weighted with Dy site occupancy; the values are likely to be somewhat underestimated; in the *C*_s_(6) isomer two well‐defined Dy—O bond lengths are 2.009(9) and 2.002(9) Å

^b)^ Estimation from magnetization curves; the X‐ray value in the site A of *C*_s_(6) isomer is 138.8(5)

^c)^ Average value from X‐ray data for main Dy sites

^d)^ Compounds with {Dy_2_O_2_} bridges are denoted by capital letters, their real composition is listed below.

**A**, [Dy(L)Cl(CH_3_OH)]_n_ (H_2_L = *N*′‐(5‐bromo‐2‐hydroxybenzylidene)pyrazine‐N‐oxide‐carbohydrazide)

**B**, [Dy_2_(a'povh)_2_(OAc)_2_(DMF)_2_] (H_2_a'povh = *N*′‐[amino(pyrimidin‐2‐yl)methylene]‐o‐vanilloyl hydrazine)

**C**, Dy_2_(HL)_2_(NO_3_)_2_(DMF)_4_ (H_3_L = 3‐hydroxy‐*N*′‐(2‐hydroxy‐3‐methoxybenzylidene)picolinohydrazide)

**D**, [Ga_4_Dy_2_(shi^3–^)_4_(Hshi^2–^)_2_(H_2_shi^–^)_2_(C_5_H_5_N)_4_(CH_3_OH)(H_2_O)] ∙ 3C_5_H_5_N ∙ 2CH_3_OH ∙ 3H_2_O (H_3_shi = salicylhydroxamic acid)

**E**, [Dy(hmac)_2_]_2_(µ‐HMq)_2_ (hmac = hexamethylacetylacetonate, HMq = 2‐methyl‐8‐hydroxyquinoline)

**F**, [Dy_2_(L1)_2_(NO_3_)_2_(MeOH)_2_] · 2MeOH (H_2_L1 = 4‐chloro‐2‐(((2‐hydroxy‐3‐methoxybenzyl )imino)methyl )phenol)

**G**, [Dy_2_(DMOP)_2_(BTFA)_4_] (DMOP = 2,6‐Dimethoxyphen, BTFA = Benzoyltrifluoroacetone)

**H**, [Dy_2_(nb)_4_(H_2_L)_2_] (H_3_L = 2‐hydroxyimino‐*N*′‐[(2‐hydroxy‐3‐methoxyphenyl)methylidene]propanohydrazone,Hnb = m‐nitrobenzoic acid)

**I**, [Dy_2_ovph_2_Cl_2_(MeOH)_3_] ∙ MeCN (H_2_ovph = pyridine‐2‐carboxylic acid [(2‐hydroxy‐3‐methoxyphenyl)methylene] hydrazide)

**J**, 2D‐layered [Dy(3‐py‐4‐pmc)(C_2_O_4_)0.5(OH)(H_2_O)] (H3‐py‐4‐pmc = 2‐(3‐pyridyl) pyrimidine‐4‐carboxylic acid)

**K**, [DyL(HCOO)(CH_3_OH)]_n_ (H_2_L = *N*′‐(2‐hydroxybenzylidene)picolinohydrazide)

**L**, Dy_2_(L2)_2_(NO_3_)_2_(MeOH)_2_ (H_2_L2 = 2‐(((2‐hydroxybenzylidene)amino)methyl )‐6‐methoxy‐phenol)

**M**, [Dy_2_(Py_3_CO)_2_(CF_3_SO_3_)_4_(H_2_O)_2_] · CH_3_CN

**N**, [Dy_2_(MeOH)_2_(HL^1^)_2_(NO_3_)_2_] · 2MeOH (H_3_L^1^ = 3‐(((2‐hydroxynaphthaen‐1‐yl)methylene)amino)‐propane‐1,2‐diol)

**O**, [Dy_2_(HL)_2_(NO_3_)_2_(DMF)_2_] · 2H_2_O (H_3_L = 3‐hydroxy‐ *N*′‐(2‐hydroxy‐3‐methoxybenzylidene)picolinohydrazide)

**P**, [Dy_2_(DMOMP)_2_(DBM)_4_]_2_ · CHCl_3_ (DMOMP = 1‐methyl‐3,5‐dimethoxy‐4‐hydroxybenzene, DBM = 1,3‐diphenylpropane‐1,3‐dione)

**Q**, [Dy_2_(HL)_2_(NO_3_)_2_(CH_3_CN)_2_] · 2CH_3_CN (H_3_L = 3‐hydroxy‐*N*′‐(2‐hydroxy‐3‐methoxybenzylidene)picolinohydrazide)

**R**, [DyLClCH_3_OH)]_2_ (H_2_L = *N*′‐(2‐hydroxybenzylidene)picolinohydrazide)

**S**, [Dy_2_(L)_2_(DBM)_2_(DMA)_2_] · 2DMA · 2CH_3_CN (H_2_L = 2‐(2‐hydroxy‐3‐methoxy‐benzylideneamino)phenol, HDBM = dibenzoylmethane, DMA = dimethylacetamide)

**T**, [Dy_2_L_2_(NO3)_2_(MeOH)_2_] (H_2_L = 2‐ethoxy‐6‐{[(2‐hydroxy‐3‐methoxybenzyl)imino]methyl}phenol).

The most common approach to the exchange interactions in {Dy_2_O_2_} SMMs is based on the pseudospin model, in which exchange Hamiltonian is written down as

(4) $${\hat{H}}_{\mathrm{exch}}\;=\;-J_{\mathrm{tot}}\hat{{\tilde{s}}_{1}}\cdot \hat{{\tilde{s}}_{2}}\;=\;-\left(J_{\mathrm{dip}}\;+\;J_{\mathrm{exch}}\right)\hat{{\tilde{s}}_{1}}\cdot \hat{{\tilde{s}}_{2}}$$

where one applies a pseudospin $\tilde{s}$ =1/2 with highly anisotropic g‐tensor to describe the ground magnetic state of each Dy ion. As Dy ground state is usually *m_J_* = ±15/2, the pseudospin g‐tensor is close to (0, 0, 20). It is not difficult to see that the FM‐AFM energy difference with this Hamiltonian is Δ*E*_AFM − FM_ = 0.5*J*_tot_cos(α) and that *J*_tot_ in Equation (4) and *j*_12_ in Equation (2) are related simply as *J*_tot_ = 450 *j*_12_ . *J*_tot_, which is usually determined from the fit to experimental magnetization data, is often further divided into dipolar and exchange contributions, *J*_dip_ and *J*_exch_. Dipolar term can be computed from the energy of the dipole‐dipole interaction:

(5) $$E_{12}^{\mathrm{dip}}\;=\;-\;\frac{\mu_{0}}{4\pi R_{12}^{3}}\left(3\left(\vec{n_{r}},\vec{\mu_{1}}\right)\left(\vec{n_{r}},\vec{\mu_{2}}\right)\;-\;\left(\vec{\mu_{1}},\vec{\mu_{2}}\right)\right),$$

where $\vec{n_{r}}$ is the normal of the radius vector connecting two magnetic moments $\vec{\mu_{1}}$ and $\vec{\mu_{2}}$, and *R*_12_ is the distance between them. $E_{12}^{\mathrm{dip}}$ can be computed exactly using experimental or DFT‐computed atomic coordinates and ab initio derived orientations of the magnetization axes and g‐tensors of individual Dy centers. The exchange term can be then determined as a difference of *J*_tot_ and *J*_dip_. Comprehensive EPR and magnetometry study showed that taking *J*_tot_ in Equation (3) as an isotropic constant is an oversimplification, as the experimental EPR spectra could be reproduced by simulation only when *J*_tot_ was treated as a tensor.^56^ However, the same authors noted that magnetometry data alone could be reasonably well reproduced by an effective scalar *J*_tot_. In the vast majority of magnetic studies of {Dy_2_O_2_} complexes the coupling was treated as isotropic, and we follow this approach in this work (keeping in mind that *J*_tot_ determined from magnetometry data should be understood as an average value).

Table 3 compares some structural and Dy∙∙∙Dy interactions parameters of Dy_2_O@C_82_ isomers to a list of selected dinuclear SMMs with {Dy_2_O_2_} bridge described in the literature. The list includes all {Dy_2_O_2_}‐SMMs with Δ*E*_AFM−FM_ exceeding 2 cm^−1^, as well as few other illustrative examples. The largest Δ*E*_AFM−FM_ difference in complexes with {Dy_2_O_2_} bridges was reported recently by Gao et al. for the chain compound, in which dinuclear moieties are further linked via chloride bridges.^57^ The Δ*E*_AFM−FM_ value for the {Dy_2_O_2_} fragment in this compound is 6 cm^−1^, *J*_tot_ amounts to 11.4 cm^−1^ (positive sign means FM interactions are favored), and *J*_dip_/*J*_exch_ constants are both positive and of similar size. Four more {Dy_2_O_2_} compounds have |Δ*E*_AFM−FM_| values exceeding 3 cm^−1^, of them three feature antiferromagnetic coupling in the ground‐state. Again, *J*_dip_/*J*_exch_ constants in the compounds with the strongest overall coupling have the same sign. For the vast majority of {Dy_2_O_2_} compounds, the |Δ*E*_AFM−FM_| is less than 3 cm^−1^. In some cases (see, e.g., last two entries in Table 3), *J*_dip_ and *J*_exch_ have relatively large values but opposite signs, leading to small overall *J*_tot_. In most cases, dipolar interactions favor FM coupling, while exchange coupling tends to be of AFM nature. To our knowledge, there is no uniform model relating structural parameters and coupling constants in {Dy_2_O_2_} bridges so far. Analysis of the structural data in Table 3 does not reveal any obvious correlation between angles, bond lengths, Dy∙∙∙Dy distances, and *J*‐constants.

Comparing then {Dy_2_O_2_} complexes to Dy_2_O@C_82_, one can find that the strongest coupling in the former is of the same size as in the *C*_3v_ isomer of Dy_2_O@C_82_, which has the weakest Dy∙∙∙Dy coupling among the three Dy_2_O@C_82_ isomers. The coupling in *C*_s_ and especially *C*_2v_ isomer is much stronger than in any {Dy_2_O_2_} complex reported to date. Further, dipolar interactions in Dy_2_O@C_82_ isomers all favor FM coupling, and because of the relatively short Dy∙∙∙Dy distances, *J*_dip_ values are comparably large and range from 8.6 to 10.2 cm^−1^. But these strong FM dipolar interactions are outweighed by even stronger AFM exchange coupling, so that the overall Dy∙∙∙Dy coupling in Dy_2_O@C_82_ is antiferromagnetic. To compensate positive *J*_dip_ constants, *J*_exch_ constants take the values from −31.8 cm^−1^ in *C*_3v_ isomer and −32.6 cm^−1^ in *C*_s_ isomer to −50.5 cm^−1^ in *C*_2v_. These are the largest exchange coupling constants in polynuclear Dy compounds aside from the complexes with radical bridges.

## 3 Conclusions

In this work, we succeeded in the synthesis of the first Dy‐oxide clusterfullerenes, the structural characterization of the three isomers of Dy_2_O@C_82_, and in depth studies of their magnetic properties. In accordance with the fourfold electron transfer expected for the Dy_2_O cluster, the three cage isomers isolated for Dy_2_O@C_82_ are *C*_s_(6), *C*_3v_(8), and *C*_2v_(9). Single crystal X‐ray diffraction studies revealed that endohedral Dy_2_O cluster in Dy_2_O@C_82_ has bent shape and features unusually short Dy—O bonds of ≈2.0 Å.

The compact geometry of Dy_2_O clusters in Dy_2_O@C_82_ leads to both strong axial ligand field and unprecedentedly strong antiferromagnetic exchange coupling between Dy ions. All three isomers of Dy_2_O@C_82_ exhibit slow relaxation of magnetization and develop a magnetic hysteresis at low temperature. In zero field, the relaxation of magnetization follows the Orbach mechanism involving the ferromagnetic excited state. Thus, the effective barriers determined from Arrhenius plots of the temperature dependence of the relaxation times are equal to the energy difference between AFM‐ and FM‐coupled states. These differences in Dy_2_O@C_82_ are considerably higher than in any SMM with Dy—O—Dy bridges reported to date. At the fields corresponding to the AFM‐FM level crossing, all isomers of Dy_2_O@C_82_ exhibit quantum tunneling of magnetization, which is apparent from the negative peaks in the field dependence of the relaxation times as well as from the temperature‐independent in‐field relaxation times.

The role of the fullerene cage in the magnetic properties of encapsulated Dy_2_O clusters, though not fully understood, is quite considerable. First, the size of exchange coupling constant in the Dy_2_O cluster varies considerably with the cage isomer. Second, the fullerene cage isomerism affects internal dynamics of the Dy_2_O cluster, which appears to correlate strongly with the relaxation of magnetization. The *C*_3v_ isomer, in which the cluster is moving almost freely, is found to be the strongest SMM among all three studied structures, whereas the *C*_s_ isomer with the fixed position of the endohedral cluster has the shortest magnetization relaxation times. In addition to dynamical properties, the size and the shape of the fullerene cage can also affect the structural parameters of the endohedral cluster, such as Dy—O bond lengths and Dy—O—Dy angle. Thus, further exploration of the Dy_2_O@C_2_ *_n_* clusterfullerene family may reveal even better SMMs.

## Conflict of Interest

The authors declare no conflict of interest.

## Supporting information

Supplementary
