# Supplementary material for: Single Molecule Magnetism with Strong Magnetic Anisotropy and Enhanced Dy∙∙∙Dy Coupling in Three Isomers of Dy‐Oxide Clusterfullerene Dy2O@C82
Source: Adv Sci (Weinh). 2019 Aug 15;6(20):1901352. doi: 10.1002/advs.201901352 (PMC6794633; doi:10.1002/advs.201901352)
Supplement: Supplementary file 1 — Supplementary [file ADVS-6-1901352-s001.pdf]

Copyright WILEY-VCH Verlag GmbH & Co. KGaA, 69469 Weinheim, Germany, 2019.

ADVANCED SCIENCE
Open Access

Supporting Information

for *Adv. Sci.,* DOI: 10.1002/advs.201901352

Single Molecule Magnetism with Strong Magnetic Anisotropy and Enhanced Dy···Dy Coupling in Three Isomers of Dy-Oxide Clusterfullerene $Dy_2O@C_{82}$

Wei Yang, Georgios Velkos, Fupin Liu, Svetlana M. Sudarkova, Yaofeng Wang, Jiaxin Zhuang, Hanning Zhang, Xiang Li, Xingxing Zhang, Bernd Büchner, Stanislav M. Avdoshenko,* Alexey A. Popov,* and Ning Chen**

Single molecule magnetism with strong magnetic anisotropy and enhanced Dy···Dy coupling in three isomers of Dy-oxide clusterfullerene $Dy_2O@C_{82}$

Wei Yang,‡ Georgios Velkos,‡ Fupin Liu,* Svetlana Sudarkova, Yaofeng Wang, Jiaxin Zhuang, Hanning Zhang, Xiang Li, Xingxing Zhang, Bernd Büchner, Stanislav M. Avdoshenko,* Alexey A. Popov,* Ning Chen*

Supporting information

Synthesis and separation S2

Single-crystal X-ray analysis S5

Crystal structures of EMF·Ni(OEP) with C_{82}-C_s(6), C_{82}-C_{3v}(8), and C_{82}-C_{2v}(9) cages S19

DFT calculations of $M_2O@C_{82}$ conformers (M = Y, Dy) S27

Comparison of DFT and X-ray structures S31

IR Spectroscopy S34

Electronic properties (UV, Echem) S36

Ab initio calculations of ligand-field splitting S39

Magnetization relaxation times S54

DFT-optimized Cartesian coordinates of $Dy_2O@C_{82}$ conformers S62

References S90

Synthesis and separation

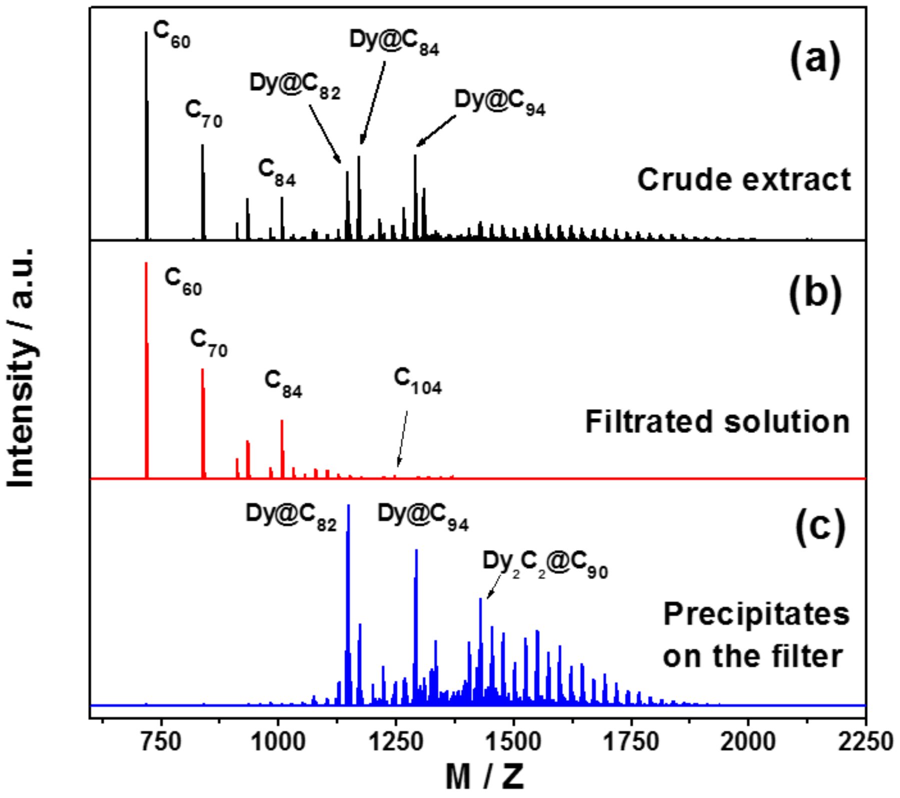

Figure S1. MALDI-TOF of (a) crude extract, (b) filtered solution, and (c) precipitates on the filter for Dy-metallofullerenes.

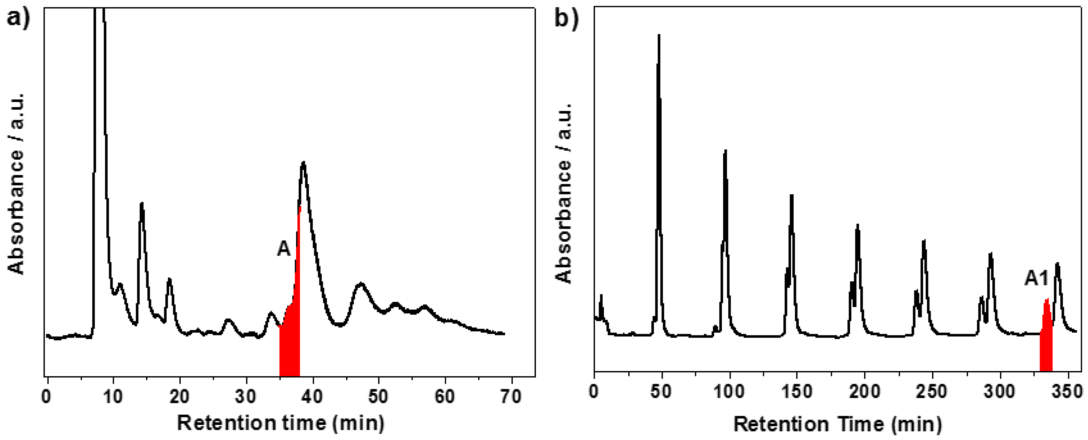

Figure S2. HPLC separation of $Dy_2O@C_s(6)\text{-}C_{82}$. a) The first stage HPLC chromatogram of extract on a Buckyprep-M column (Φ = 25 mm × 250 mm) and b) the second stage HPLC chromatogram of fraction A on a Buckyprep column (Φ = 10 mm × 250 mm). The HPLC conditions were: eluent = toluene; flow rate = 4 mL/min; detecting wavelength = 310 nm.

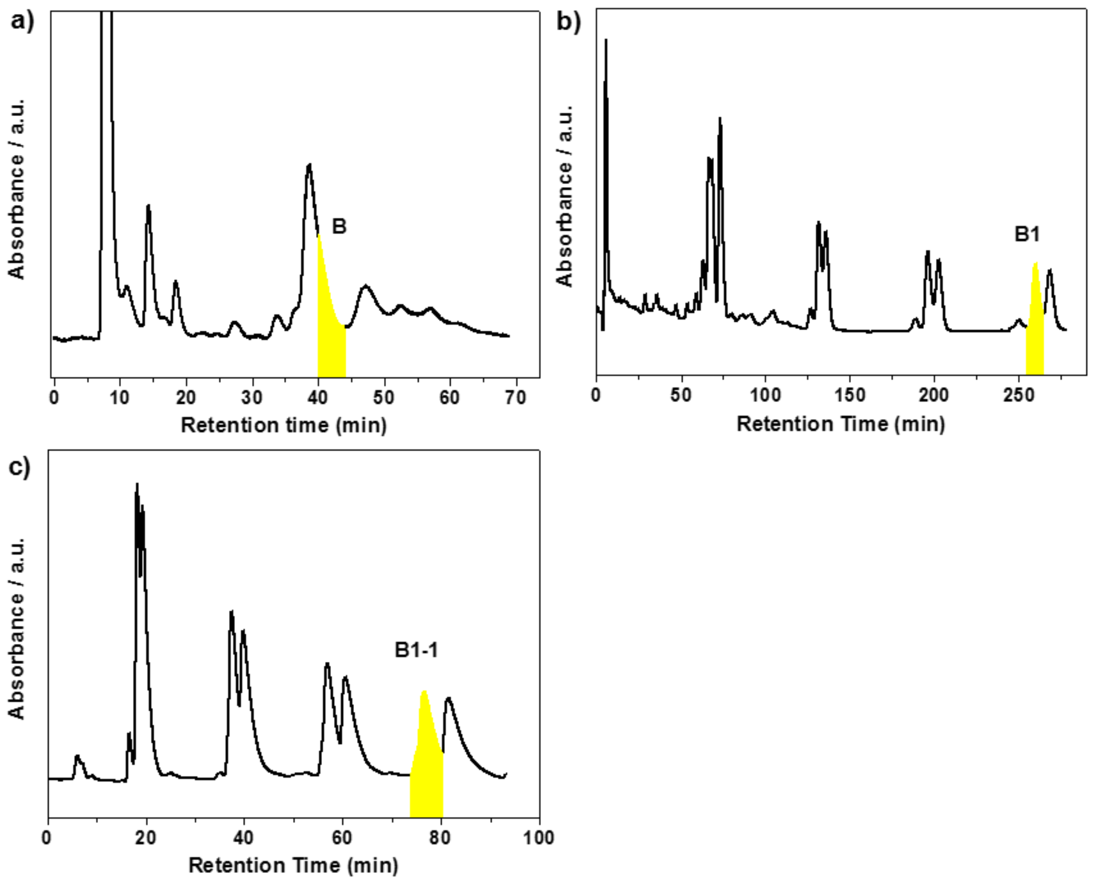

Figure S3. HPLC separation of $Dy_2O@C_{2v}(9)\text{-}C_{82}$. a) The first stage HPLC chromatogram of extract on a Buckyprep-M column (Φ = 25 mm × 250 mm), b) the second stage HPLC chromatogram of fraction B on a Buckyprep column (Φ = 10 mm × 250 mm) and c) the third stage HPLC chromatogram of fraction B1 on a Buckyprep-D column (Φ = 10 mm × 250 mm). The HPLC conditions were: eluent = toluene; flow rate = 4 mL/min; detecting wavelength = 310 nm.

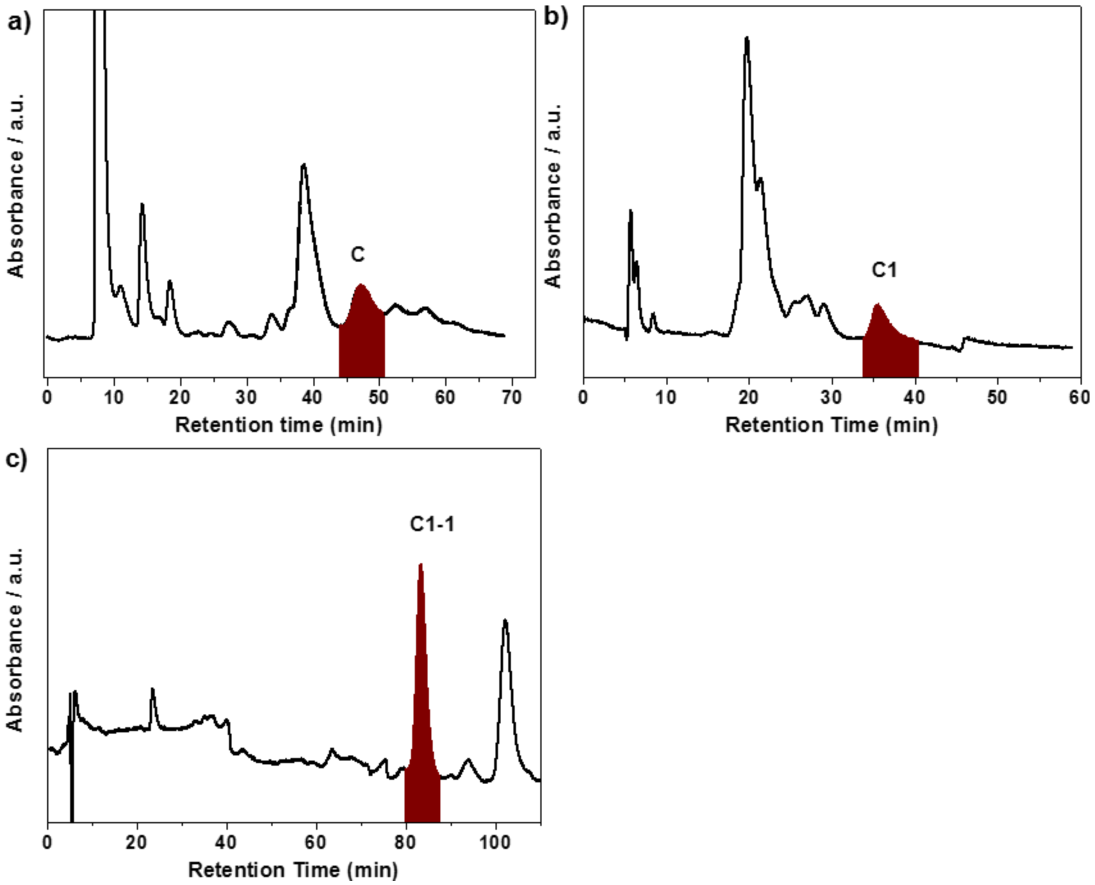

Figure S4. HPLC separation of $Dy_2O@C_{3v}(8)$-C_{82}. a) The first stage HPLC chromatogram of extract on a Buckyprep-M column (Φ = 25 mm × 250 mm), b) the second stage HPLC chromatogram of fraction C on a Buckyprep-D column (Φ = 10 mm × 250 mm) and c) the third stage HPLC chromatogram of fraction C1 on a Buckyprep column (Φ = 10 mm × 250 mm). The HPLC conditions were: eluent = toluene; flow rate = 4 mL/min; detecting wavelength = 390 nm.

X-ray analysis

Crystals were grown by layering the benzene solution of nickel octaethylporphyrin (Ni(OEP)) onto the CS_2 solution of the $Dy_2O@C_{82}$ isomers. The as-prepared crystals suitable for X-ray diffraction analysis were measured with a diffractometer. Specifically, $Dy_2O@C_s(6)\text{-}C_{82}\cdot Ni(OEP)\cdot 2(C_6H_6)$ was measured at 100 K using the wavelength of 0.82653 Å with an CCD detector at beamline BL17U1 of the Shanghai Synchrotron Radiation Facility (SSRF). The structure was found to be a twin. Specifically, on the basis of indexing using the program CELL_NOW, the crystal was determined to be a two-component, nonmerohedral twin with the domains related by a rotation of 179.9 degrees about the direct and reciprocal [1 0 0] axis. $Dy_2O@C_{3v}(8)\text{-}C_{82}\cdot Ni(OEP)\cdot 1.5(C_6H_6)\cdot CS_2$ and $Dy_2O@C_{2v}(9)\text{-}C_{82}\cdot Ni(OEP)\cdot C_6H_6$ were measured with Bruker APEX II at room temperature and 173 K, respectively. The structures were solved by direct methods and refined using all data (based on F^2) by SHELX 2016.[1] Hydrogen atoms were located in a difference map, added geometrically, and refined with a riding model. There is a fourth benzene site at the C19S and C20S with severe disorder present in the $Dy_2O@C_s(6)\text{-}C_{82}\cdot Ni(OEP)\cdot 2(C_6H_6)$ lattice. Similarly, a second benzene site at the C7S and C8S with severe disorder is present in the $Dy_2O@C_{2v}(9)\text{-}C_{82}\cdot Ni(OEP)\cdot C_6H_6$ lattice. The structure of $Dy_2O@C_{3v}(8)\text{-}C_{82}\cdot Ni(OEP)\cdot 1.5(C_6H_6)\cdot CS_2$ is a pseudo-merohedral twin with twin law (0 -1 0 1 0 0 0 0 -1) and refined with twin parameter of 0.49271. The crystal data are presented in Table S1. The data can be obtained free of charge from the Cambridge Crystallographic Data Centre with CCDC Nos. 1908347-9.

Figure S5 compares the mutual relationship between sites A and B of $Dy_2O@C_s(6)\text{-}C_{82}\cdot Ni(OEP)$. The fullerene cage of site A is fully ordered, while the fullerene cage of site B is slightly disordered, even though we modeled it with the fully ordered structure. Most of the metal sites locate at similar positions in the fullerene cage except for the strong differences of the site occupancies (Fig. S6).

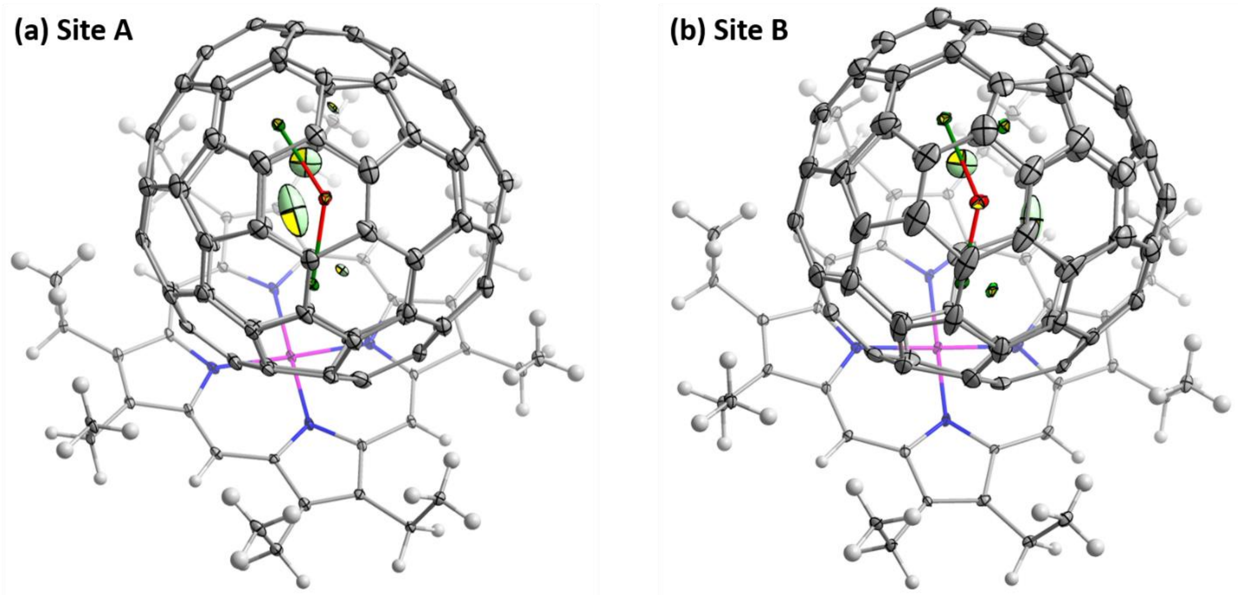

Figure S5. Comparison between sites A and B of $Dy_2O@C_s(6)\text{-}C_{82}\cdot Ni(OEP)$. The displacement parameters are shown at the 10% probability level. Color code: grey for carbon, green for Dy, red for O, blue for N, white for H, and purple for Ni. The disordered Dy sites are highlighted with the brightness of the green color to differentiate the site occupancies, the darker the color, the higher the occupancy.

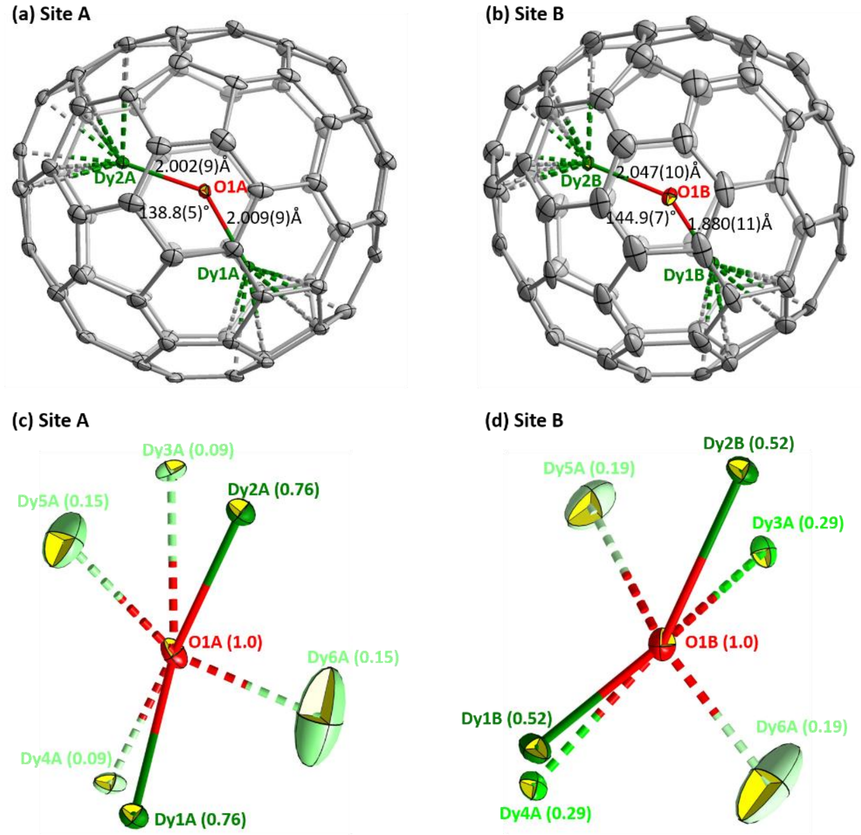

Figure S6. (**a, b**) Comparison between sites A (**a**) and B (**b**) of $Dy_2O@C_s(6)-C_{82}$. Only the main site of Dy_2O cluster is shown to highlight the bond lengths and angles. (**c, d**) Sites A (**c**) and B (**d**) of the encapsulated Dy_2O cluster of $Dy_2O@C_s(6)-C_{82}$. Distribution of the disordered Dy sites are highlighted with the brightness of the green color to differentiate the site occupancies, the darker the color, the higher the occupancy. The site occupancies are noted in parenthesis. The displacement parameters are shown at the 10% probability level. Color code: grey for carbon, green for Dy, and red for O.

Table S1a. Crystal data

	$Dy_2O@C_s(6)$-C_{82}·Ni(OEP)·2(C_6H_6)	$Dy_2O@C_{3v}(8)$-C_{82}·Ni(OEP)·1.5(C_6H_6)·CS_2	$Dy_2O@C_{2v}(9)$-C_{82}·Ni(OEP)·C_6H_6
**Formula**	C256 H106 Dy4 N8 Ni2 O2	C128 H53 Dy2 N4 Ni O S2	C128 H50 Dy2 N4 Ni O
**Formula weight**	4092.9	2110.57	2043.43
**Color, habit**	Black, block	Black, block	Black, block
**Crystal system**	triclinic	triclinic	monoclinic
**Space group**	$P\overline{1}$	$P\overline{1}$	$C2/m$
***a*, Å**	15.006(3)	16.046(3)	25.335(3)
***b*, Å**	19.968(4)	16.097(3)	15.2798(13)
***c*, Å**	25.324(5)	17.686(4)	19.8904(19)
***α*, deg**	85.38(3)	75.79(3)	90
***β*, deg**	89.36(3)	75.76(3)	96.099(10)
***γ*, deg**	88.66(3)	64.13(3)	90
**Volume, Å^3**	7561(3)	3933.6(18)	7656.2(13)
Z	2	2	4
***T*, K**	100	296	173
**Radiation (λ, Å)**	Synchrotron (0.82653)	Mo K-α (0.71073)	Cu K-α (1.54187)
**Unique data (R_{int})**	26156 (0)	14101 (0.0844)	7039 (0.0378)
**Parameters**	2515	1307	708
**Restraints**	1002	1879	752
**Observed data**	22625	8387	6253
**R_1[a]**	0.1479	0.1323	0.1234
**wR_2[b]**	0.4042	0.4232	0.3527
**CCDC NO.**	1908347	1908348	1908349

[a]For observed data with $I > 2\sigma(I)$, $R_1 = \frac{\sum||F_o|-|F_c||}{\sum|F_o|}$. [b]For all data, $wR_2 = \sqrt{\frac{\sum[w(F_o^2-F_c^2)^2]}{\sum[w(F_o^2)^2]}}$.

Table S1b. Occupancy of Dy sites, Dy–O distances, and selected Dy–O–Dy angles from X-ray diffraction data

	Dy site occupancy	Dy–O bond lengths (Å)[1)]	Selected Dy–O–Dy angles (°)[2)]
$Dy_2O@C_s(6)$-C_{82}	Dy1A: 0.764(2)<br>Dy2A: 0.764(2)<br>Dy3A: 0.087(2)<br>Dy4A: 0.087(2)<br>Dy5A: 0.149(2)<br>Dy6A: 0.149(2)<br><br>Dy1B: 0.519(2)<br>Dy2B: 0.519(2)<br>Dy3B: 0.289(2)<br>Dy4B: 0.289(2)<br>Dy5B: 0.192(2)<br>Dy6B: 0.192(2)	Dy1A–O1A: 2.009(9)<br>Dy2A–O1A: 2.002(9)<br>Dy3A–O1A: 2.172(11)<br>Dy4A–O1A: 1.723(12)<br>Dy5A–O1A: 2.001(17)<br>Dy6A–O1A: 1.80(2)<br><br>Dy1B–O1B: 1.880(11)<br>Dy2B–O1B: 2.047(10)<br>Dy3B–O1B: 1.816(12)<br>Dy4B–O1B: 2.056(10)<br>Dy5B–O1B: 1.910(13)<br>Dy6B–O1B: 1.843(17)	Dy1A–O1A–Dy2A: 138.8(5)<br><br><br><br><br><br><br>Dy1B–O1B–Dy2B: 144.9(6)
$Dy_2O@C_{3v}(8)$-C_{82}	Dy1: 0.518(3)<br>Dy2: 0.280(3)<br>Dy3: 0.308(3)<br>Dy4: 0.144(3)<br>Dy5: 0.184(3)<br>Dy6: 0.291(3)<br>Dy7: 0.137(3)<br>Dy8: 0.139(3)	Dy1-O1: 1.997(7)<br>Dy2-O1: 2.012(9)<br>Dy3-O1: 2.009(8)<br>Dy4-O1: 1.900(14)<br>Dy5-O1: 1.952(11)<br>Dy6-O1: 1.987(8)<br>Dy7-O1: 1.952(12)<br>Dy8-O1: 1.888(13)	Dy1–O1–Dy2: 164.8(7)<br>Dy1–O1–Dy6:164.8(7)<br>Dy3–O1–Dy2:165.3(7)<br>Dy3–O1–Dy6:136.9(6)<br>Dy5–O1–Dy2: 131.9(8)<br>Dy5–O1–Dy6: 158.8(6)
$Dy_2O@C_{2v}(9)$-C_{82}	Dy1: 0.360(2)<br>Dy2: 0.279(3)<br>Dy3: 0.162(5)<br>Dy4: 0.067(3)<br>Dy5: 0.061(2)<br>Dy6: 0.089(3)<br>Dy7: 0.055(2)<br>Dy8: 0.041(2)	Dy1-O1: 1.908(5)<br>Dy2-O1: 1.991(7)<br>Dy3-O1: 1.970(9)<br>Dy4-O1: 1.976(17)<br>Dy5-O1: 1.954(13)<br>Dy6-O1: 1.896(10)<br>Dy7-O1: 1.999(16)<br>Dy8-O1: 1.885(18)	Dy1–O1–Dy2: 116.8<br>Dy1–O1–Dy2’: 152.7<br>Dy1–O1–Dy3: 135.8

[1)] Due to the strong disorder in Dy positions, Dy–O bond length for Dy sites with low occupancy are probably not very reliable.

[2)] Dy–O–Dy angles are listed only for Dy sites with high occupancy. Note that because of the disorder of Dy sites, it is not always possible to identify real Dy_2O cluster positions, and hence the values may not necessary correspond to valence angles in Dy_2O cluster.

Table S2. Dy–O bond lengths shorter than 2.1 Å (X-ray diffraction data)

Dy–O bond environment	Dy–O bond length (Å)	Reference
$[Dy_5O(O^iPr)_{13}]$	1.951(18)-2.66(2)	1
$[(dipp\text{-}Bian)Ga\text{-}Ga(dipp\text{-}Bian)](C_4H_8O)DyI(THF)_5$	2.005(5)-2.444(4)	2
$[Dy(1\text{-}O_2CDBP)(DBP)_2]_2$	2.034(6)-2.277(7)	3
$[Dy_4K_2O(O^tBu)_{12}]$	2.068(10)-2.515(10)	4
$[Dy(CH[PPh_2NSiMe_3]_2)(I)]_2(\mu\text{-}O)$	2.03499(19)	5
$[Cp'Dy(\mu\text{-}H)(OAr)]_2$	2.0826(19)	6
$[Dy(OC_2H_4O^iPr)_3]_8$	2.079(5)-2.667(6)	7
$[Dy(\mu\text{-}ONep)_2(ONep)]_4$	2.049(3)-2.303(5)	8
$Dy_3(\mu_3\text{-}ONep)_2(\mu\text{-}ONep)_3(ONep)_4(py)_2$	2.073(5)-2.510(5)	8
$Dy(DMP)_3(py)_3$	2.077(5)-2.118(5)	8
$Dy_3(\mu_3\text{-}O^tBu)_2(\mu\text{-}O^tBu)_3(O^tBu)_4(HO^tBu)_2$	2.089(3)-2.579(3)	8
$Dy(DIP)_3(NH_3)_2$	2.036(11)-2.143(13)	8
$Dy_3(\mu_3\text{-}O^tBu)_2(\mu\text{-}O^tBu)_3(O^tBu)_4(THF)_2$	2.077(5)-2.577(6)	8
$Dy(DIP)_3(THF)_2$	2.089(2)-2.372(2)	8
$Dy_3(\mu\text{-}DMP)_4(DMP)_5(NH_3)_2$	2.023(12)-2.326(12)	8
$[Dy(\eta^6\text{-}DIP)(DIP)_2]_2$	2.053(8)-2.123(8)	8
$Dy(DBP)_3(NH_3)$	2.0886(19)-2.0996(19)	8
$Dy(DBP)_3$	2.047(3)-2.060(3)	8
$Dy(DBP)_3(THF)$	2.078(4)-2.392(4)	8
$[Dy(\mu\text{-}TPS)(TPS)_2]_2$	2.071(3)-2.301(3)	8
$DyLi(Ph_3CO)_3(NPh_2)THF$	2.068(3)-2.195(3)	9
$Cp_2Ln(THF)OAlL(Me)$	2.052(5), 2.364(5)	10
$Dy[^tBu_2CHO]_3CH_3CN$	2.057-2.063	11
$[K(crypt)][((^{Ad,Me}ArO)_3mes)DyOH]$	2.087(12)-2.683(13)	12
$((^{Ad,Me}ArO)_3mes)Dy$	2.093(3)-2.095(3)	12
$[K(18\text{-}crown\text{-}6)(THF)_2][((^{Ad,Me}ArO)_3mes)Dy]$	2.066(3)-2.188(3)	12
$[K(crypt)][((^{Ad,Me}ArO)_3mes)DyH]$	2.095(3)-2.182(3)	12
$[Dy(\mu\text{-}OH)(DBP)_2(THF)]_2$	2.0943(17)-2.3843(18)	13
$[Dy(^tBuO)Cl(THF)_5][BPh_4]\cdot 2THF$	2.043(4)-2.426(3)	14

Orientation of C_{82}-EMF cages in co-crystals with Ni(OEP)

Table S3. EMF with C_s(6), C_{3v}(8), and C_{2v}(9) isomers of C_{82} cage characterized by single-crystal X-ray diffraction in the form of co-crystals with Ni(OEP) or Co(OEP).

EMF	*q*	C_s(6)	C_{3v}(8)	C_{2v}(9)
$Dy_2O@C_{82}$	−4	Ia, t.w.	I, t.w.	I, t.w.
$Sc_2O@C_{82}$	−4	Ib, Ref. [15]	IIb, Ref. [16]	–
$Sc_2S@C_{82}$	−4	Ia, Ref. [17]	IIa, Ref. [17]	–
$Dy_2S@C_{82}$	−4	Ib, Ref. [18]	IIa, Ref. [18]	–
$Sc_2C_2@C_{82}$	−4	–	IIa, Ref. [19]	–
$Tb_2C_2@C_{82}$	−4	Ia, Ref. [20]	–	–
$Tm_2C_2@C_{82}$	−4	Ia, Ref.[21]	–	–
$Sc_3N@C_{82}$	−6	–	–	VI, Ref. [22]
$Lu_3N@C_{82}$	−6	–	–	V, Ref. [23]
$YCN@C_{82}$	−2	II, Ref. [24]	–	–
$TbCN@C_{82}$	−2	III, Ref. [25]	–	IV, Ref. [25]
$Y_2@C_{82}$	−4	Ia, Ref. [26]	III, Ref. [26]	–
$Er_2@C_{82}$	−4	Ia, Ref. [27]	IIb, Ref. [28]	–
$Tm_2@C_{82}$	−4	III, Ref.[21]	–	–
$Lu_2@C_{82}$	−4	Ia, Ref. [29]	III, Ref. [29]	–
$Sc@C_{82}$	−3	–	–	V, Ref. [30]
$Y@C_{82}$	−3	III, dimer, Ref. [31]	–	V, Ref. [30, 31]
$La@C_{82}$	−3	–	–	III, Ref. [30, 32]
$Ce@C_{82}$	−3	–	–	III, dimer, Ref. [30]
$Sm@C_{82}$	−2	III, Ref. [33]	–	III, Ref. [34]
$Gd@C_{82}$	−3	–	–	II, Ref. [35]
$Er@C_{82}$	−3	III, dimer, Ref. [36]	–	VII, Ref. [36]
$Tm@C_{82}$	−2	III, Ref. [37]	–	–
$Yb@C_{82}$	−2	III, Ref. [38]	–	IV, Ref. [38]
$Th@C_{82}$	−4	–	IIb, Ref. [39]	–
$U@C_{82}$	−3	–	–	VII, Ref. [40]
		**17**	**9**	**15**

Cage-Ni(OEP) Orientation types found in the analysis of single-crystal X-ray structures

EMF-C_s(6)-C_{82}:

Type Ia: Dy_2O, Tb_2C_2, Tm_2C_2, Sc_2S, Er_2, Lu_2, Y_2

Type Ib: Dy_2S, Sc_2O

Type II: YCN

Type III: TbCN, Yb, Sm, Tm, Tm_2, Y, Er

EMF-C_{3v}(8)-C_{82}:

Type I: Dy_2O

Type IIa: Sc_2S, Dy_2S, Sc_2C_2

Type IIb: Sc_2O, Er_2, Th

Type III: Lu_2, Y_2

EMF-C_{2v}(9)-C_{82}:

Type I: Dy_2O

Type II: Gd

Type III: La, Sm, Ce

Type IV: Yb, TbCN, Y

Type V: Y, Sc, Lu_3N

Type VI: Sc_3N

Type VII: U, Er

Crystal structures of EMF-C_s(6)-C_{82}·Ni(OEP) co-crystals, only Ni(OEP) molecule and main fullerene orientation are shown

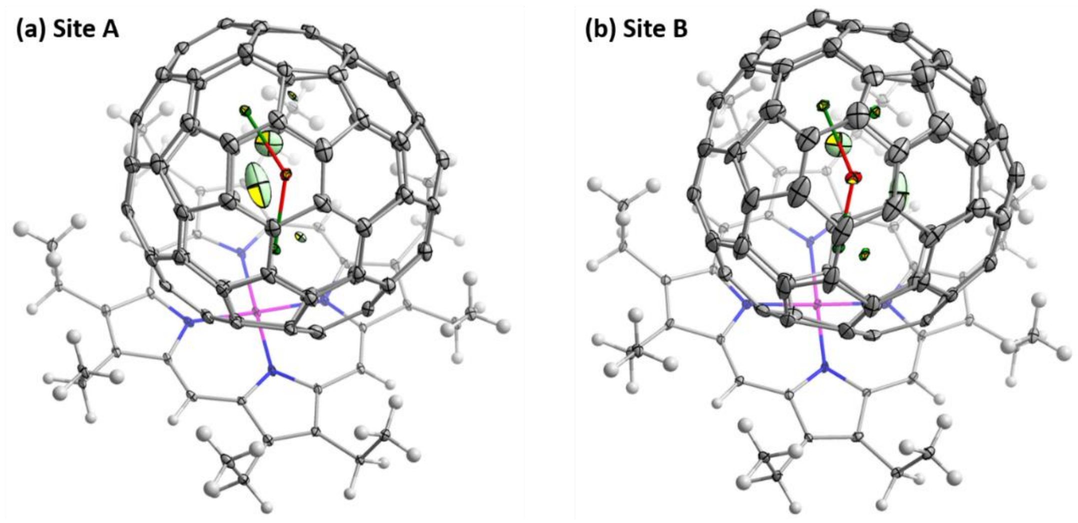

Figure S7. $Dy_2O@C_s(6)-C_{82}$·Ni(OEP)·2(C_6H_6)

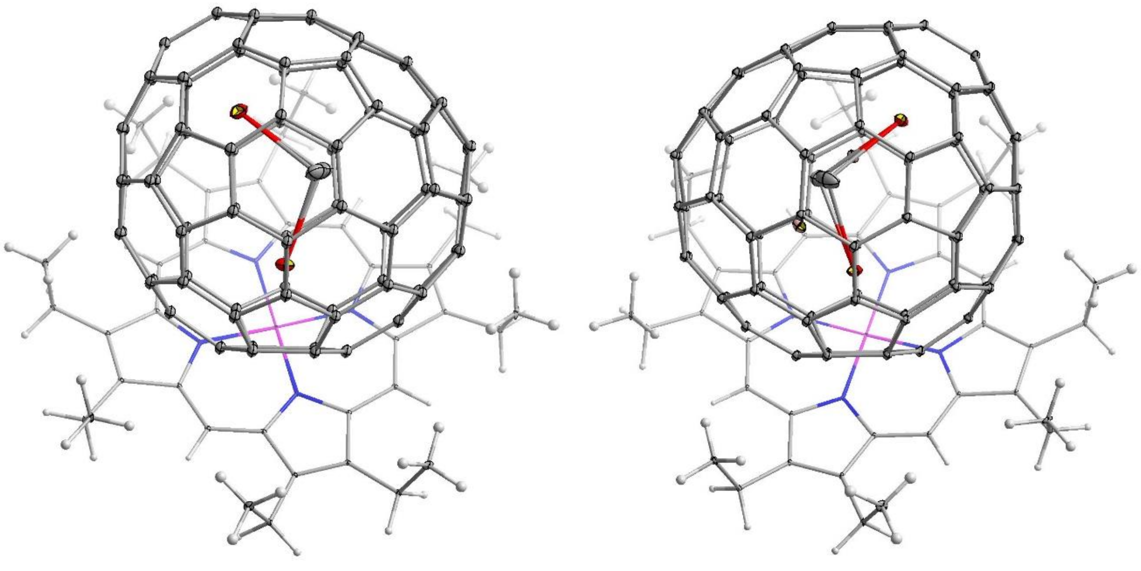

Figure S8. $Tb_2C_2@C_s(6)-C_{82}$·Ni(OEP)·2(C_6H_6)[20]

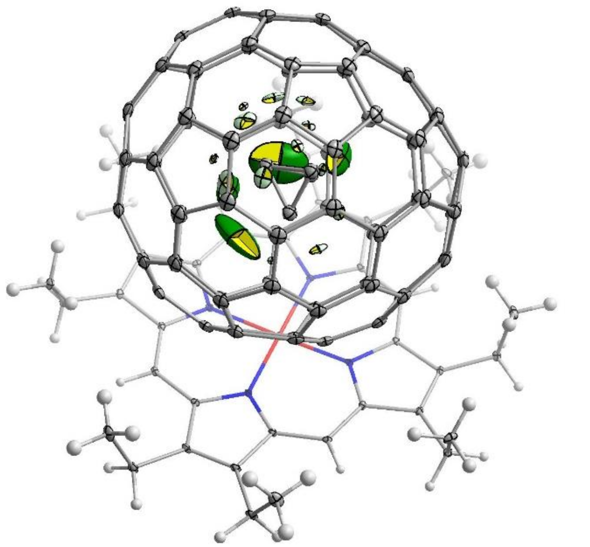

Figure S9. $Tm_2C_2@C_s(6)$-C_{82}·Ni(OEP)·1.906($C_6H_5Cl_1$)·0.093($CHCl_3$)[21]

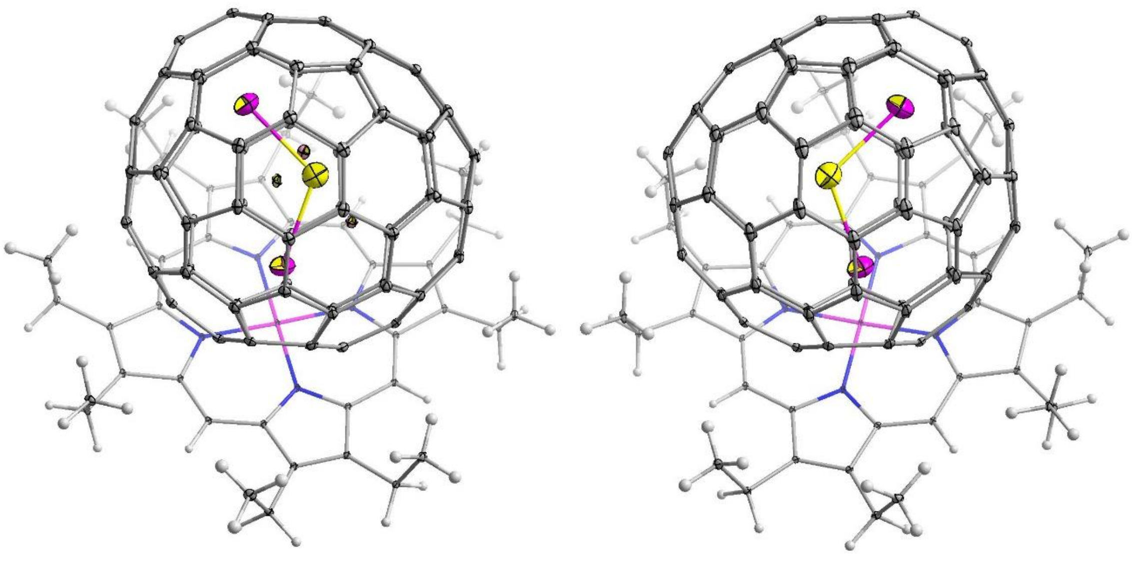

Figure S10. $Sc_2S@C_s(6)$-C_{82}·Ni(OEP)·2(C_6H_6)[17]

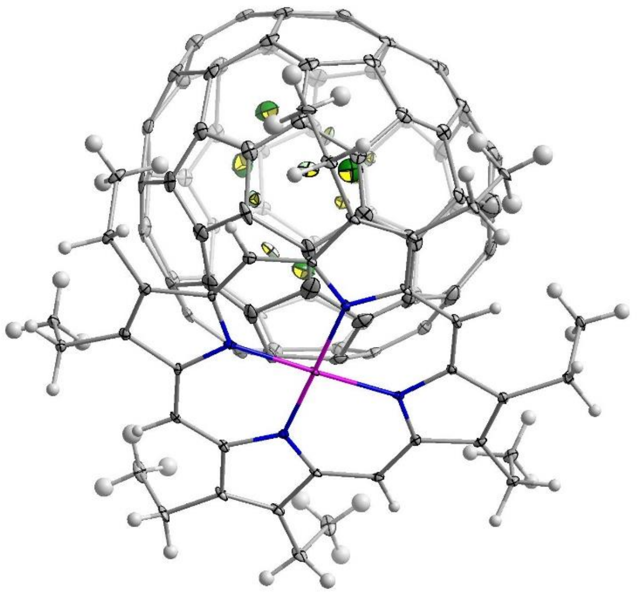

Figure S11. $Dy_2S@C_s(6)$-C_{82}·2Ni(OEP)·2toluene[18]

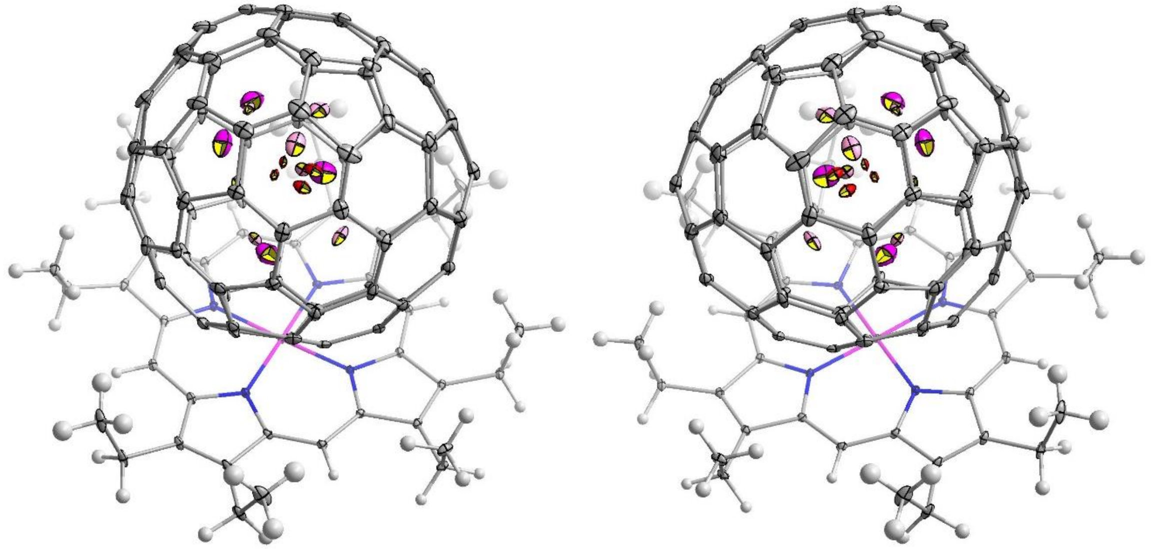

Figure S12. $Sc_2O@C_s(6)$-C_{82}·Ni(OEP)·1.4toluene·0.6(C_6H_6), four orientations[15]

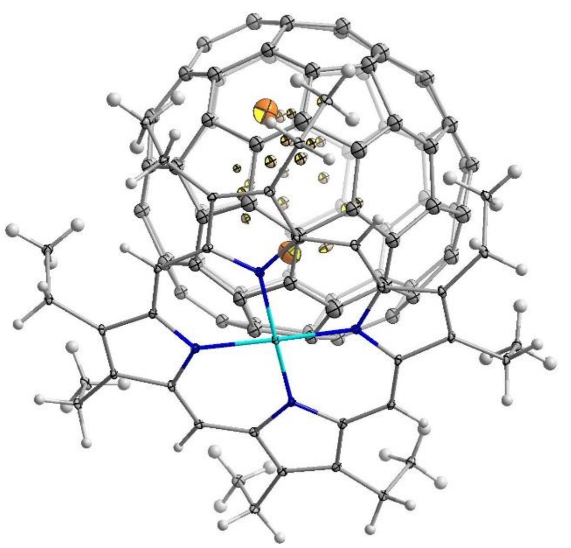

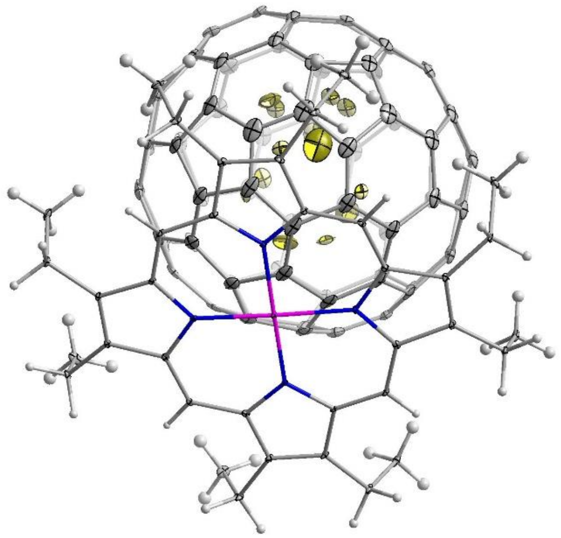

Figure S13. $Er_2@C_s(6)$-C_{82}·Co(OEP)·1.4(C_6H_6)·0.3($CHCl_3$)[27] **Figure S14.** $Lu_2@C_s(6)$-C_{82}·Ni(OEP)·2(C_6H_6)[29]

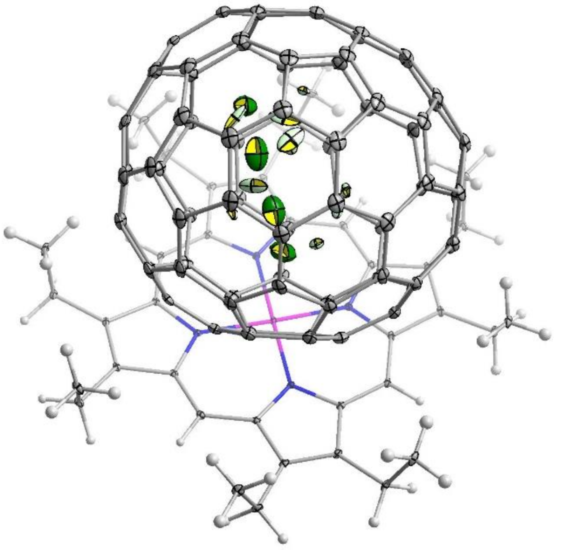

Figure S15. $Y_2@C_s(6)$-C_{82}·2Ni(OEP)·1.7(C_6H_6)·0.3(CS_2)[26]

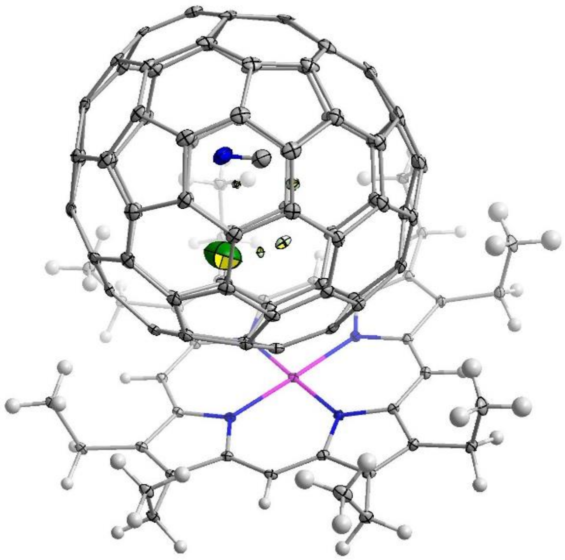

Figure S16. YCN@C_s(6)-C_{82}·Ni(OEP)·1.73(C_6H_6)·1.27($CHCl_3$)[24]

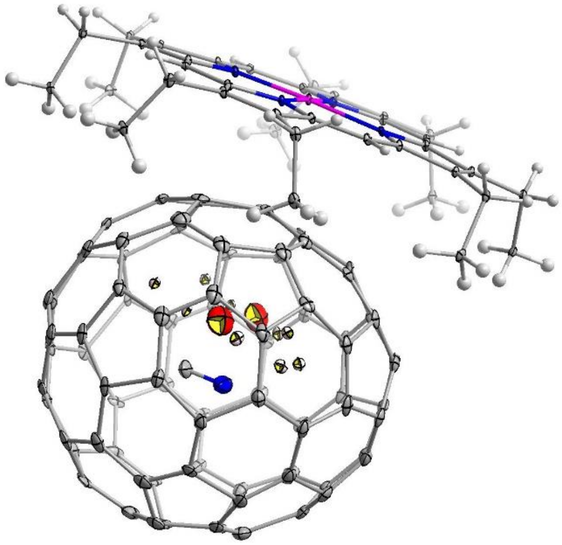

Figure S17. TbCN@C_s(6)-C_{82}·Ni(OEP)·2(C_6H_6)[25]

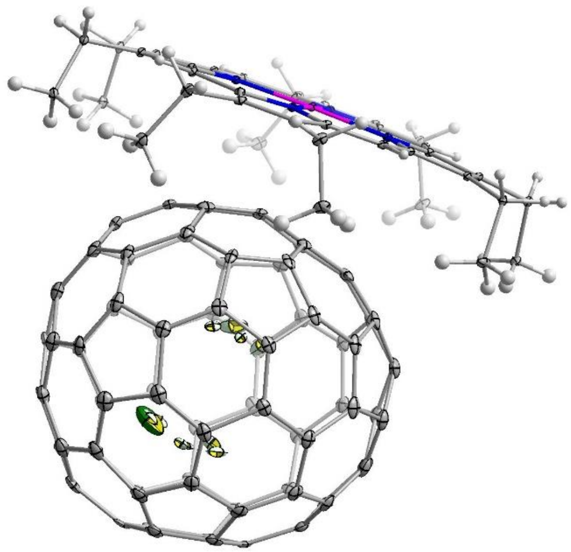

Figure S18. Yb@C_s(6)-C_{82}·Ni(OEP)·2(C_6H_6)[38]

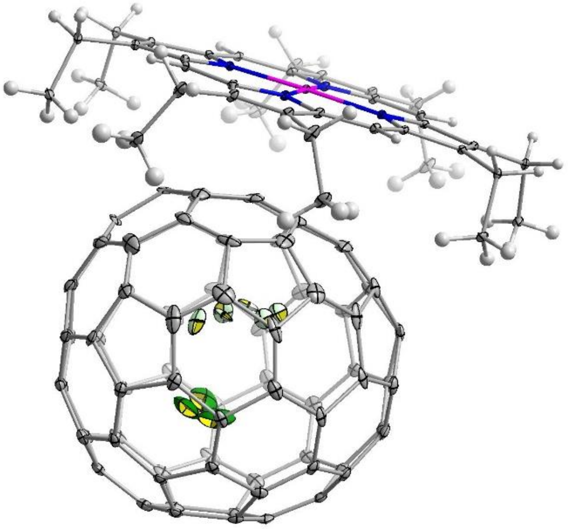

Figure S19. Sm@0.667C_{3v}(7)-C_{82}/0.333C_s(6)-C_{82}·Ni(OEP)·2toluene[33]

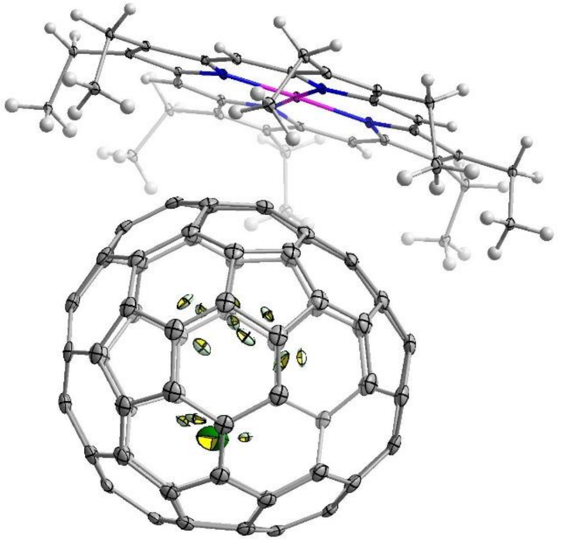

Figure S20. Tm@C_s(6)-C_{82}·Ni(OEP)·1.7($CHCl_3$)[37]

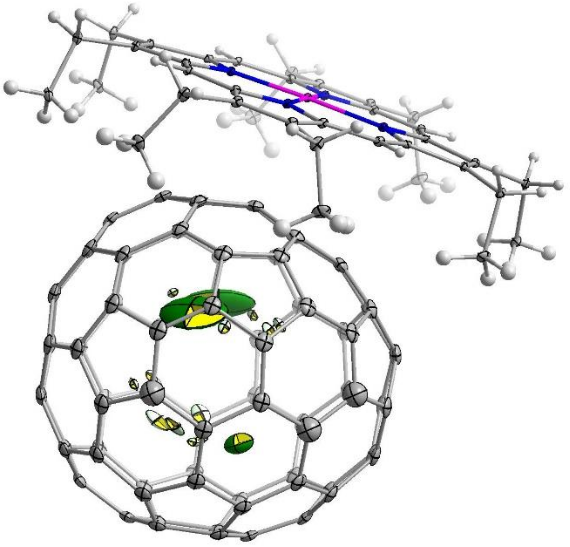

Figure S21. Tm_2@C_s(6)-C_{82}·Ni(OEP)·1.776($C_6H_5Cl_1$)·0.224($CHCl_3$)[21]

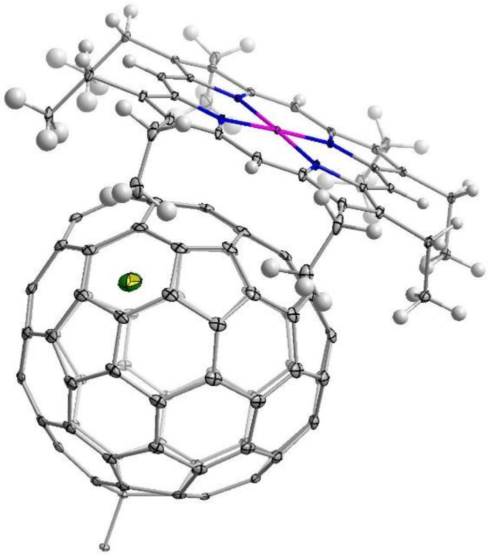

Figure S22. 2Y@C_s(6)-C_{82}·2Ni(OEP)·3(C_6H_6)·2(CS_2)[31]

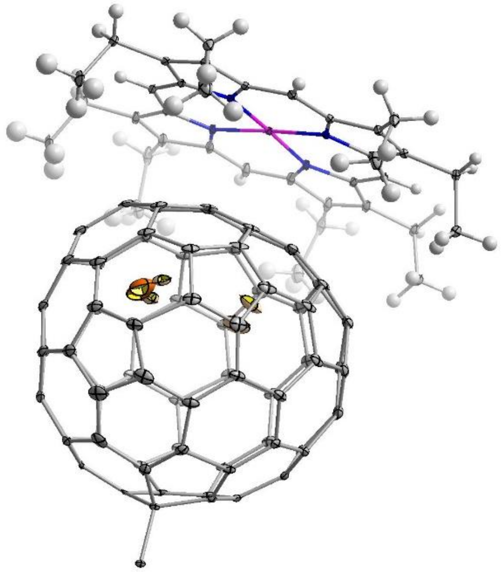

Figure S23. 2Er@C_s(6)-C_{82}·2Ni(OEP)·3(C_6H_6)·2(CS_2)[36]

Crystal structures of EMF-C_{3v}(8)-C_{82}·Ni(OEP) co-crystals, only Ni(OEP) molecule and main fullerene orientation are shown

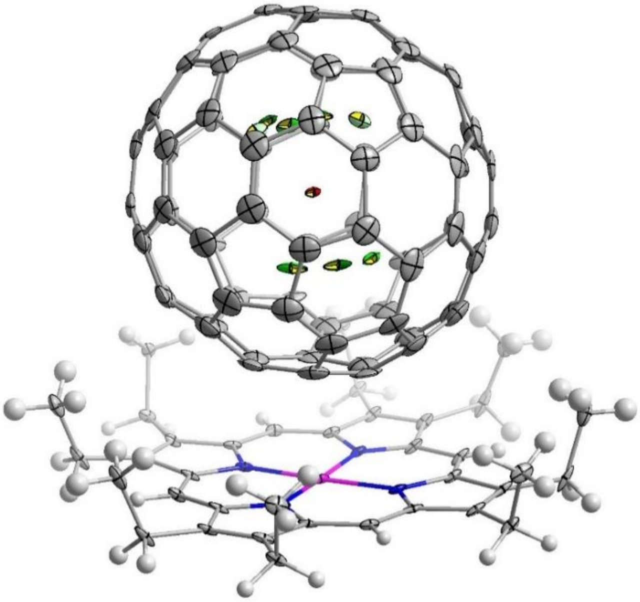

Figure S24. $Dy_2O@C_{3v}(8)-C_{82}$ · Ni(OEP)·1.5(C_6H_6)·CS_2

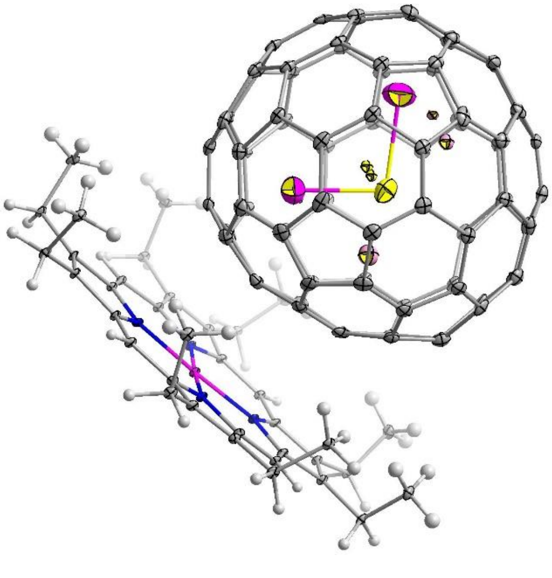

Figure S25. $Sc_2S@C_{3v}(8)-C_{82}$·Ni(OEP)·2(C_6H_6)[17]

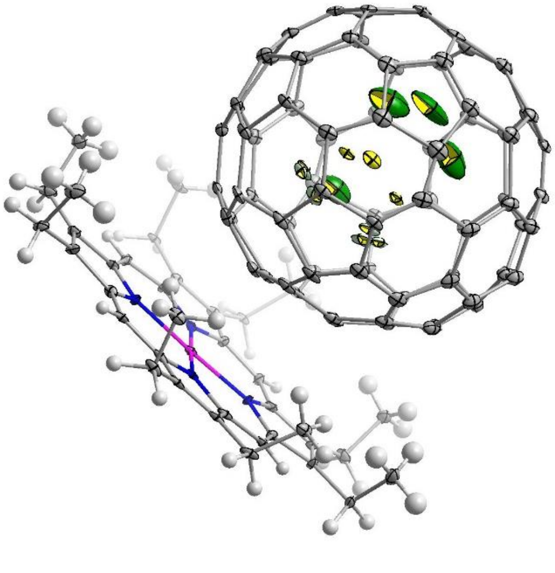

Figure S26. $Dy_2S@C_{3v}(8)-C_{82}$·Ni(OEP)·2toluene[18]

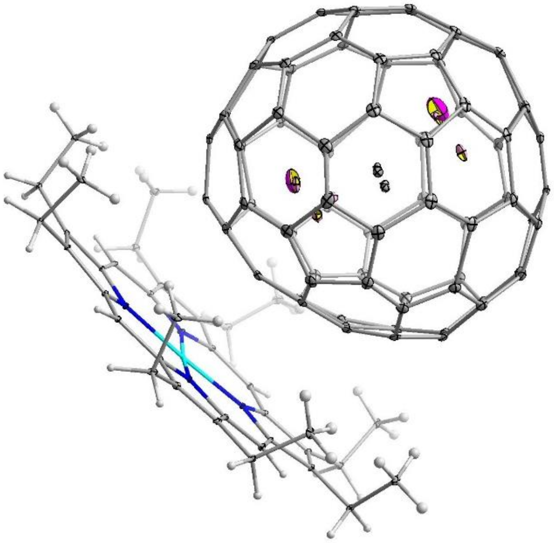

Figure S27. $Sc_2C_2@C_{3v}(8)-C_{82}\cdot Co(OEP)\cdot x(CHCl_3)$[19]

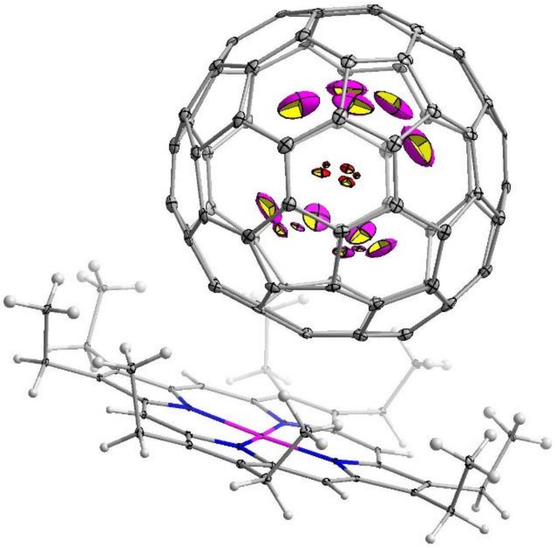

Figure S28. $Sc_2O@C_{3v}(8)-C_{82}\cdot Ni(OEP)\cdot 0.9(C_6H_6)\cdot 0.1(CHCl_3)$[16]

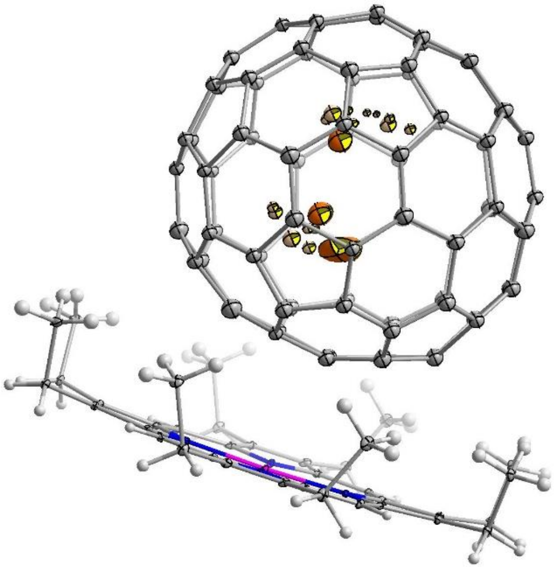

Figure S29. $Er_2@C_{3v}(8)-C_{82}\cdot Ni(OEP)\cdot 2(C_6H_6)$[28]

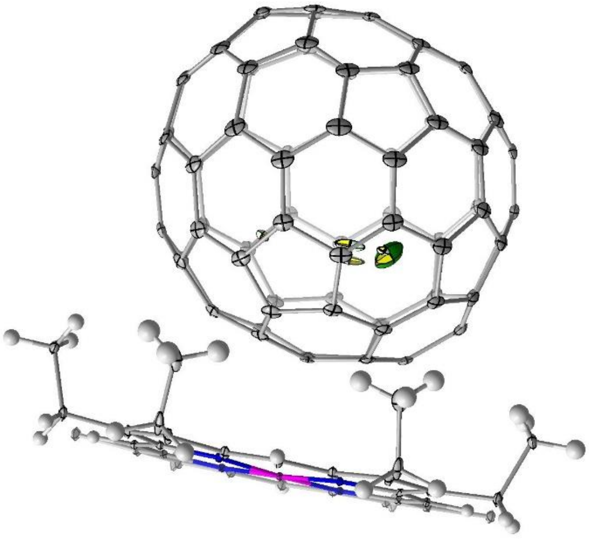

Figure S30. $Th@C_{3v}(8)-C_{82}\cdot Ni(OEP)\cdot 1.5(C_6H_6)\cdot CS_2$[39]

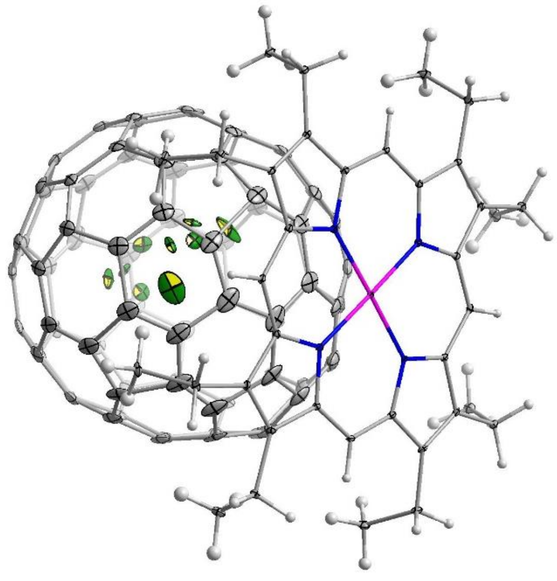

Figure S31. $Lu_2@C_{3v}(8)-C_{82}\cdot Ni(OEP)\cdot 1.16(CS_2)\cdot 0.84(CHCl_3)$[29]

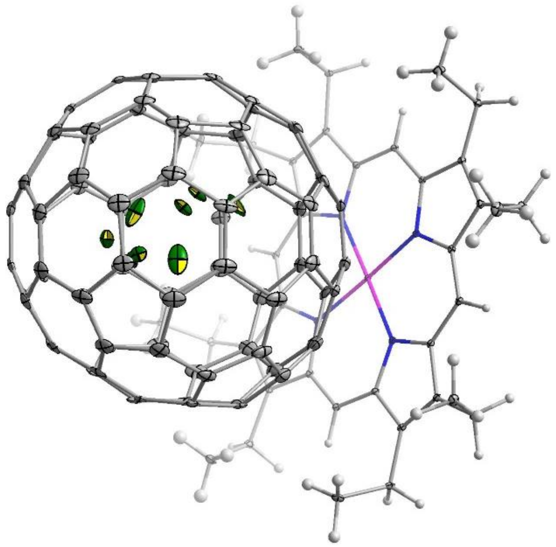

Figure S32. $Y_2@C_{3v}(8)-C_{82}\cdot Ni(OEP)\cdot x(C_6H_6)$[26]

Crystal structures of EMF-C_{2v}(9)-C_{82}·Ni(OEP) co-crystals, only Ni(OEP) molecule and main fullerene orientation are shown

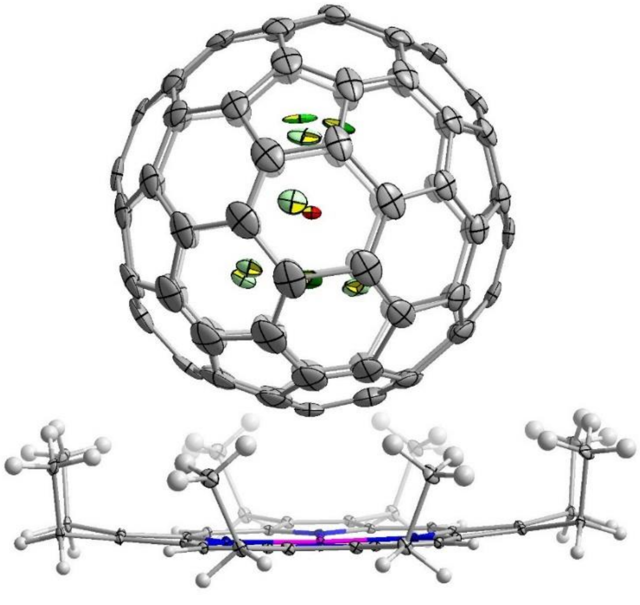

Figure S33. $Dy_2O@C_{2v}(9)\text{-}C_{82}\cdot Ni(OEP)\cdot C_6H_6$

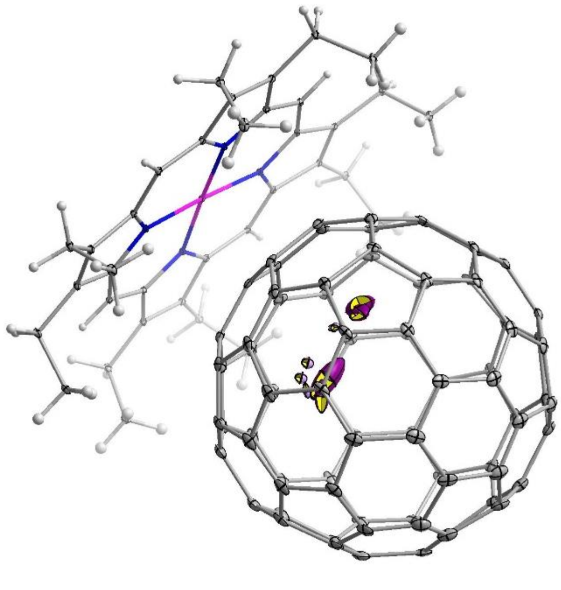

Figure S34. $Gd@C_{2v}(9)\text{-}C_{82}\cdot Ni(OEP)\cdot 1.4(C_6H_6)\cdot 0.6(CHCl_3)$[35]

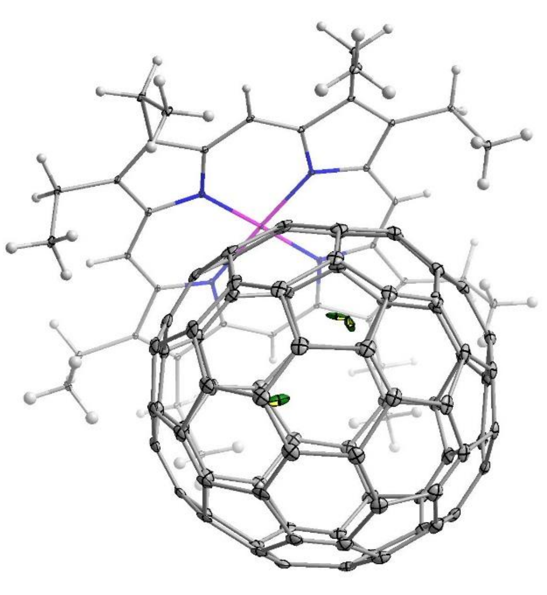

Figure S35. La@C_{2v}(9)-C_{82}·Ni(OEP)·2(C_6H_6)[32]

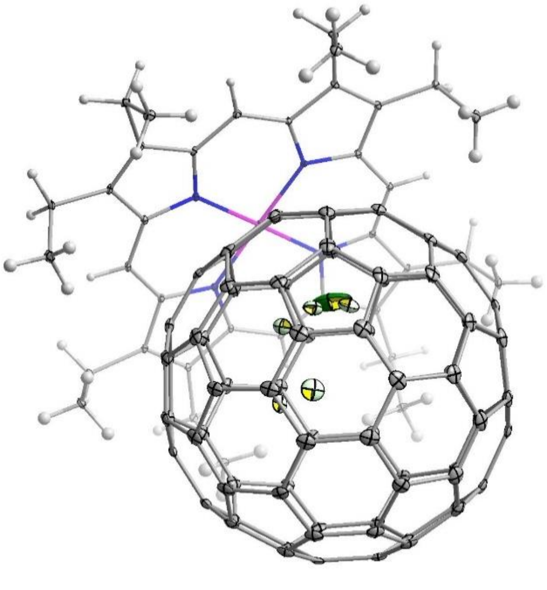

Figure S36. La@C_{2v}(9)-C_{82}·Ni(OEP)·2(C_6H_6)[30]

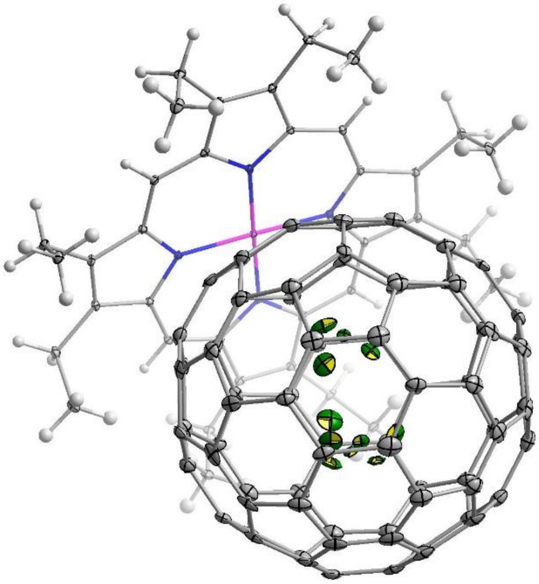

Figure S37. Sm@C_{2v}(9)-C_{82}·Ni(OEP)·0.87(C_6H_6)·0.13($CHCl_3$) (4 orientations)[34]

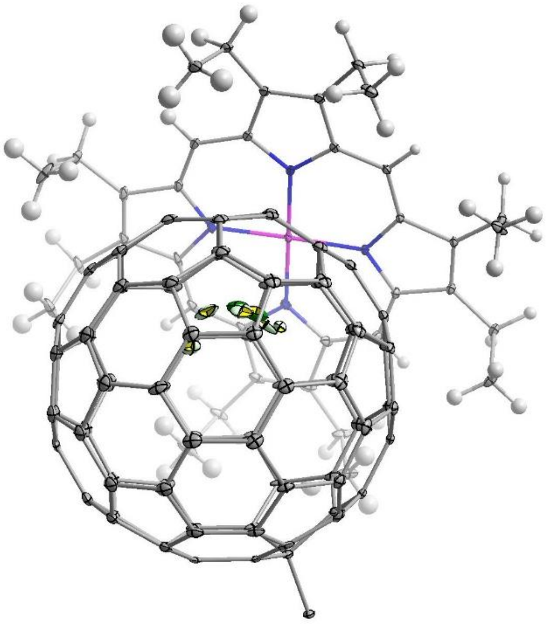

Figure S38. Ce@C_{2v}(9)-C_{82}·Ni(OEP)·2(C_6H_6)[30]

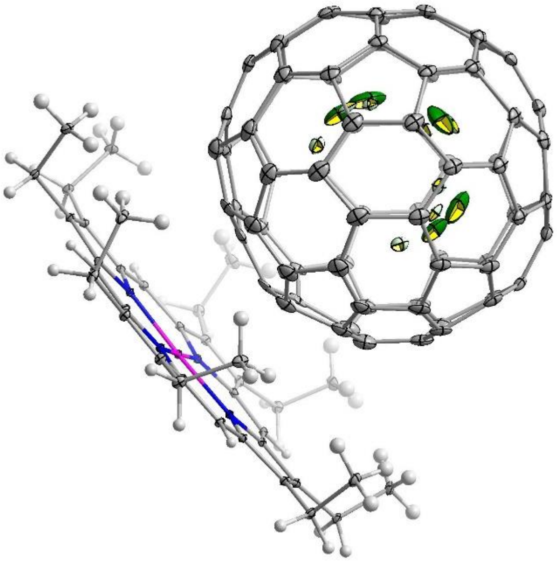

Figure S39. Yb@C_{2v}(9)-C_{82}·Ni(OEP)·2(C_6H_6), three orientations[38]

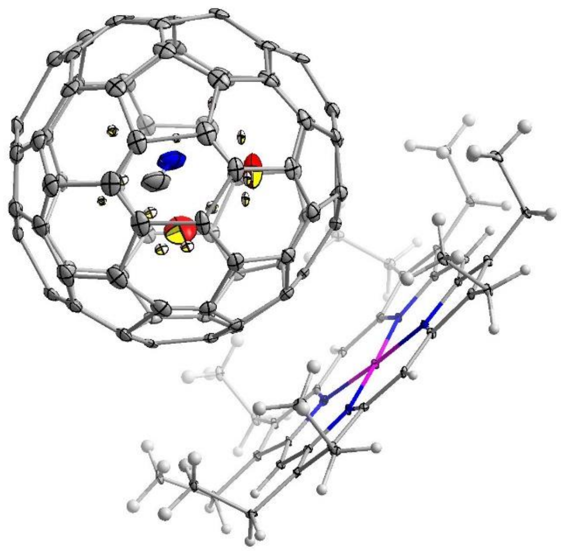

Figure S40. TbCN@C_{2v}(9)-C_{82}·Ni(OEP)·2(C_6H_6)[25]

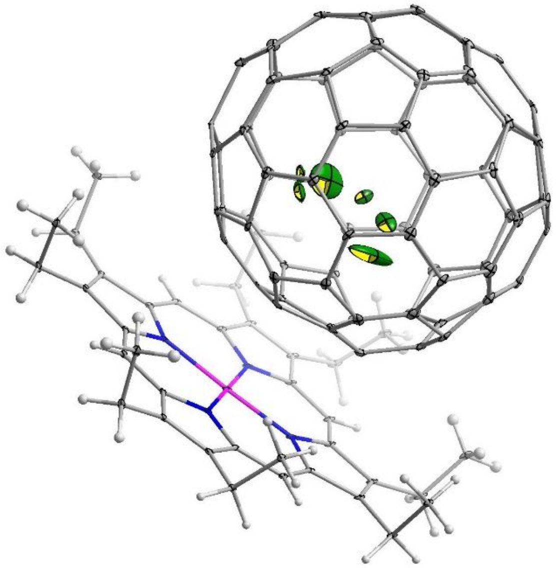

Figure S41. 2Y@C_{2v}(9)-C_{82}·2Ni(OEP)·3(C_6H_6)·2(CS_2)[31]

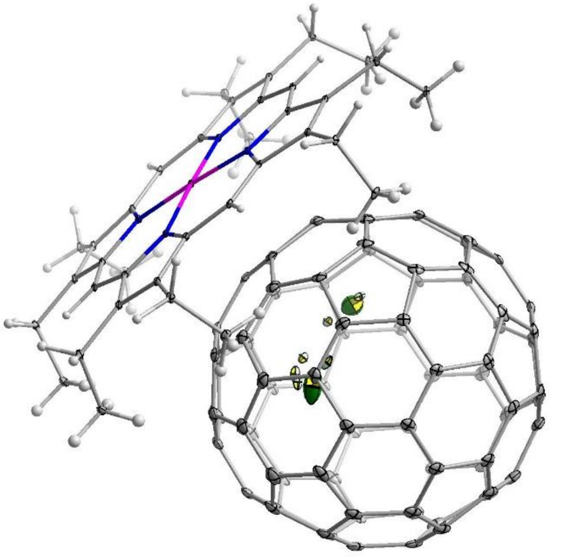

Figure S42. Y@C_{2v}(9)-C_{82}·Ni(OEP)·1.38(C_6H_6)·0.62($CHCl_3$)[30]

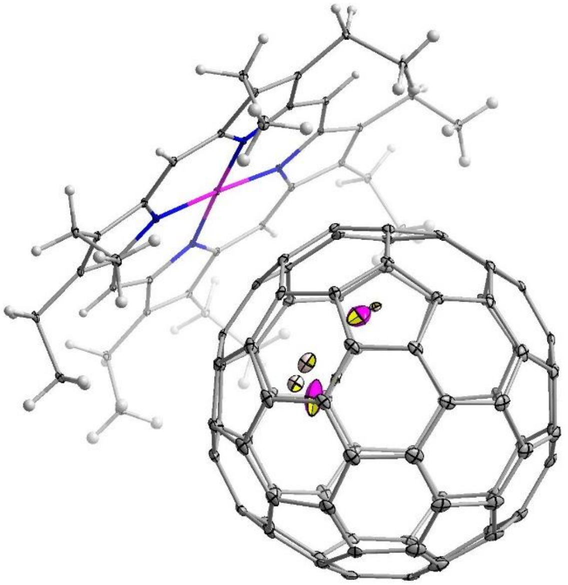

Figure S43. Sc@C_{2v}(9)-C_{82}·Ni(OEP)·1.36(C_6H_6)·0.64($CHCl_3$)[30]

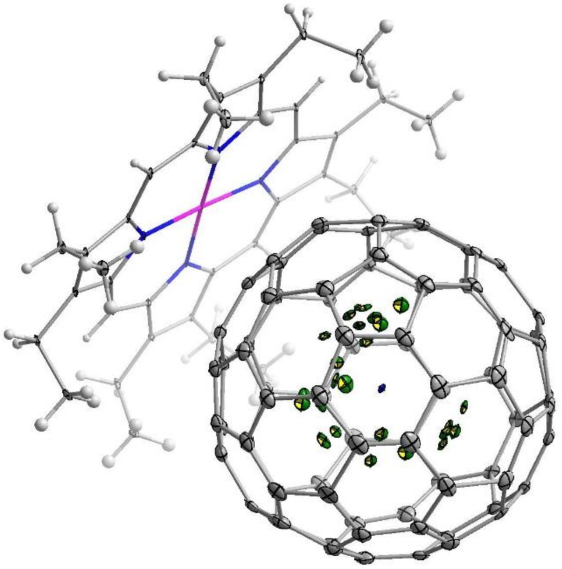

Figure S44. Lu_3N@C_{2v}(9)-C_{82}·Ni(OEP)·2(C_6H_6)[23]

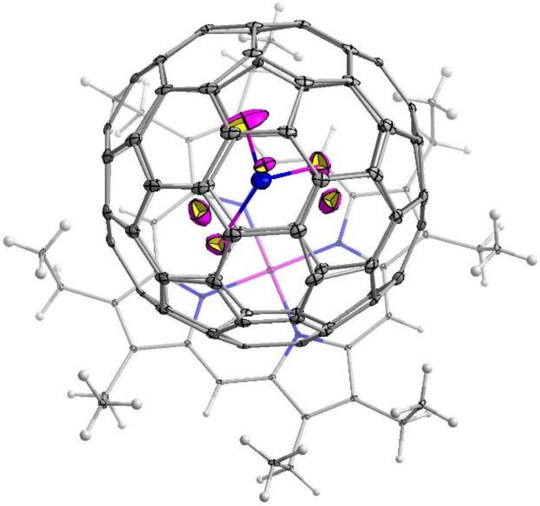

Figure S45. Sc_3N@C_{2v}(9)-C_{82}·Ni(OEP)·2(C_6H_6)[22]

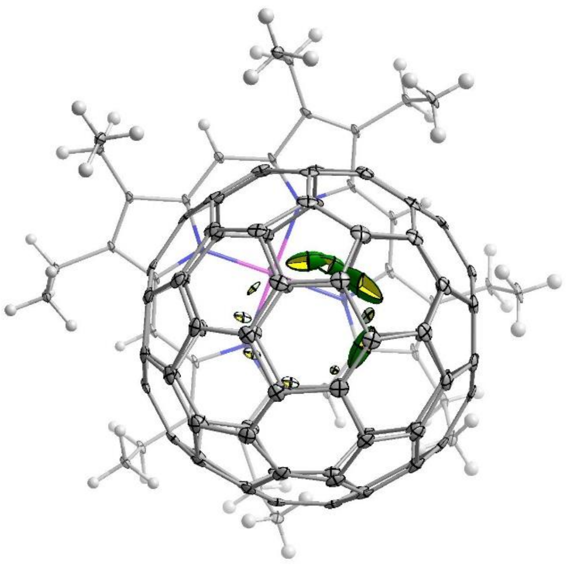

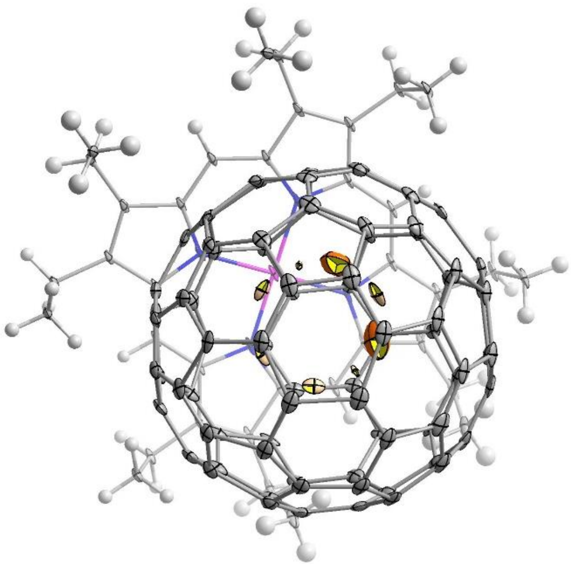

Figure S46. U@C_{2v}(9)-C_{82}·Ni(OEP)·1.5(C_6H_6)·CS_2[40] **Figure S47.** Er@C_{2v}(9)-C_{82}·Ni(OEP)·1.93(C_6H_6)·0.57(CS_2)[36]

DFT calculations of cluster conformers

The conformer search algorithm included 3 major steps. In the first one, all possible orientations of M_2O cluster inside the C_{82} cages are generated by the rotation over the Fibonacci nodes (shown at Figure S48, S50, S52; also see main text for details). At the second step, each conformer was optimized at PBE/TZ2P level using M=Y substitution (Priroda code, version 6), which led to a limited number of unique conformers, that further were optimized at the PBE/PAW level of theory using M=Dy substitution (VASP code). The relative energies for the conformers are summarized in Table S4-S6 for cages symmetries C_s, C_{3v}, and C_{2v}, respectively.

As it was argued in the main text, the Y substitution is a very accurate approximation, but in some cases, the potential energy has a very complicated and shallow topology and so the minima location may become sensitive to the optimization procedure. Thus, pre-optimization with M=Y inadvertently might have overlooked some small barrier local minimum accessible for M=Dy. To ensure the comprehensive consideration and most accurate geometric fit between theory and experiment, we optimized set of the $Dy_2O@C_{82}$ conformers for all cage symmetries with starting geometries constructed based on X-ray observed. The main sites for C_s and C_{3v} symmetries were already well-predicted by the search algorithm, thus no new structures were detected this way. However, in the C_{2v} cage, one new conformer (**5**) was detected. This conformer is less stable by 14.2 kJ/mol then the most stable one in the set. However structurally, this conformer is closely connected to one of the algorithm predicted conformer (Table S6). All this indicates the high complexity of the potential energy surface.

Conformers of $M_2O@C_s(6)$-C_{82}

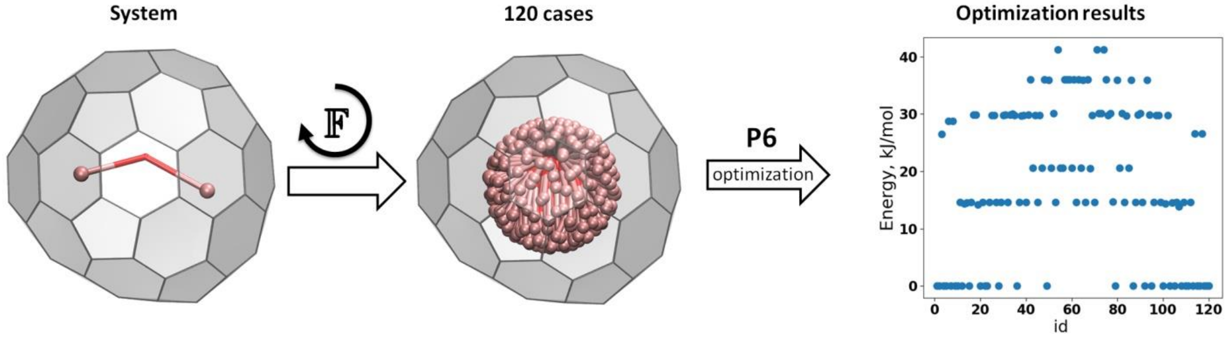

Figure S48. Left: the original C_{82}-C_s system. Center: superposed all 120 initial configurations of the Y_2O cluster regenerated with Fibonacci algorithm (𝔽). Right: relative energies of $Y_2O@C_{82}$ conformers after DFT optimization at P6/PBE/TZ2P level.

Table S4. Relative energies (ΔE, kJ/mol) and M–O–M angles (°) in $M_2O@C_{82}$-C_s conformers

	$Y_2O@$ C_{82}-C_s		$Dy_2O@$ C_{82}-C_s	
# Conformer	ΔE	Y–O–Y	ΔE	Dy–O–Dy
**1**	0.0	135.2	0.0	136.1
**2**	14.3	157.9	20.6	169.0
**3**	14.6	158.0	24.3	135.5
**4**	20.4	135.5	25.6	165.6
**5**	26.5	157.0	32.0	132.4
	28.7	153.7		
**6**	20.6	135.5	34.7	160.2
**7**	29.8	132.8	39.9	143.4
	36.0	141.8		

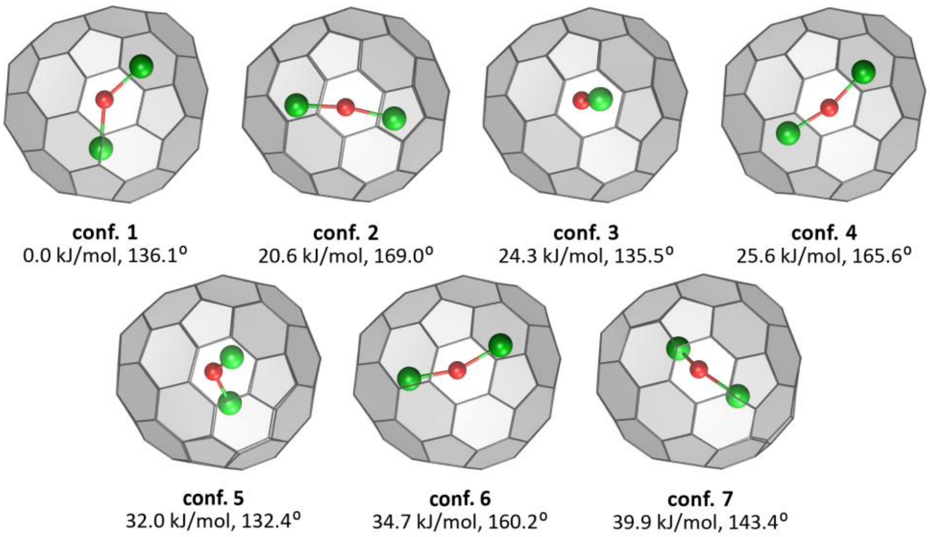

Figure S49. Stable unique conformers of $Dy_2O@C_{82}$-C_s as predicted at PBE/PAW level.

Conformers of $M_2O@C_{3v}(8)$-C_{82}

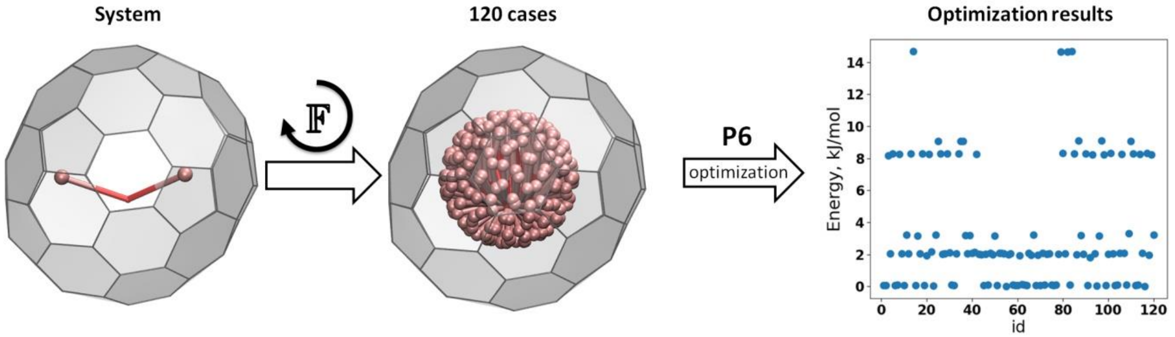

Figure S50. Left: the original C_{82}-C_{3v} system. Center: superposed all 120 initial configurations of the Y_2O cluster regenerated with Fibonacci algorithm (𝔽). Right: relative energies of $Y_2O@C_{82}$ conformers after DFT optimization at P6/PBE/TZ2P level.

Table S5. Relative energies (Δ*E*, kJ/mol) and M–O–M angles (°) in $M_2O@C_{82}$-C_{3v} conformers

	$Y_2O@$ C_{82}-C_{3v}		$Dy_2O@$ C_{82}-C_{3v}	
# Conformer	Δ*E*	Y–O–Y	Δ*E*	Dy–O–Dy
**1**	0.0	142.9	0.0	145.2
**2**	3.1	137.9	0.2	139.1
**3**	2.0	133.6	0.9	133.1
	14.6	131.7	0.9	133.1
**4**	8.1	165.1	9.9	172.7
**5**	9.0	134.0	10.5	132.7

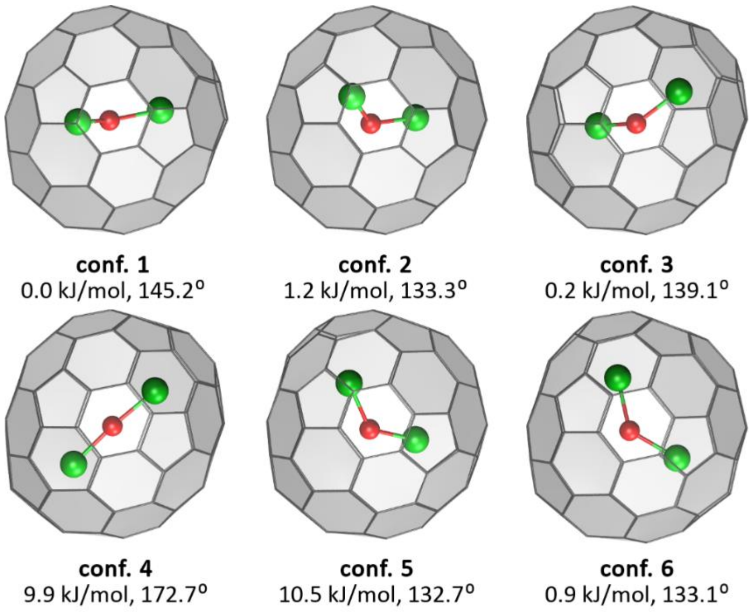

Figure S51. Stable unique conformers of $Dy_2O@C_{82}$-C_{3v} as predicted at PBE/PAW level.

Conformers of $M_2O@C_{2v}(9)$-C_{82}

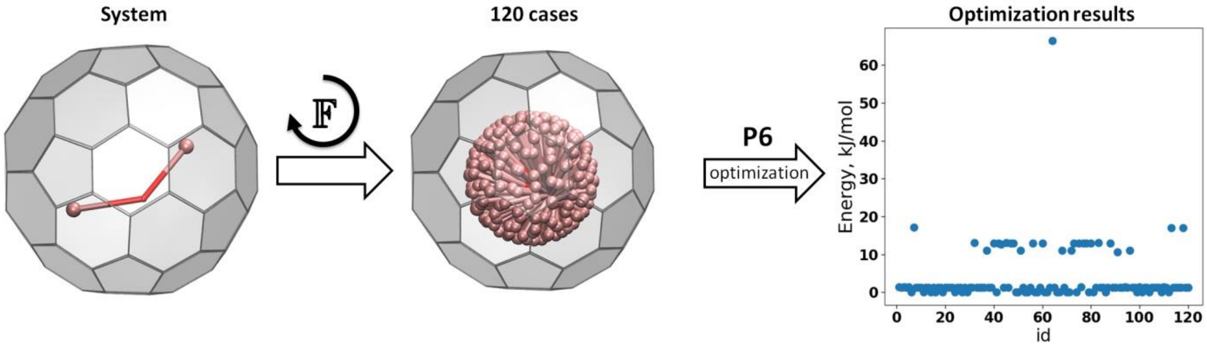

Figure S52. Left: the original C_{82}-C_{2v} system. Center: superposed all 120 initial configurations of the Y_2O cluster regenerated with Fibonacci algorithm (𝔽). Right: relative energies of $Y_2O@C_{82}$ conformers after DFT optimization at P6/PBE/TZ2P level.

Table S6. Relative energies (Δ*E*, kJ/mol) and M–O–M angles (°) in $M_2O@C_{82}$-C_{2v} conformers

	**Y_2O**@ C_{82}-C_{2v}		**Dy_2O**@ C_{82}-C_{2v}	
# Conformer	Δ*E*	Y–O–Y	Δ*E*	Dy–O–Dy
**1**	1.3	137.5	-1.9	138.9
**2**	0.0	143.8	0.0	145.9
**3**	11.0	136.5	11.2	138.3
**4**	13.0	137.0	12.9	137.5
**5**	*	*	14.2	154.8
**6**	13.0	146.2	15.9	150.1
**7**	17.1	144.6	16.1	148.6
**8**	66.4	126.3	63.3	125.7

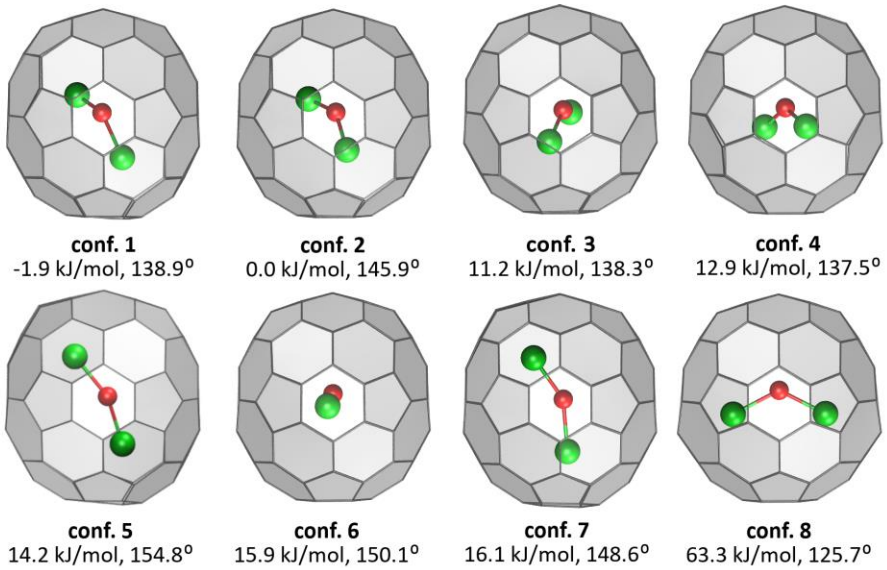

Figure S53. Stable unique conformers of $Dy_2O@C_{82}$-C_{2v} as predicted at the PBE/PAW level.

Correspondence between DFT-optimized conformes and X-ray structure of $Dy_2O@C_s(6)$-C_{82}

For the site A of $Dy_2O@C_s(6)$-C_{82} X-ray diffraction gives three sites for the Dy_2O cluster. These sites were used as starting coordinates for DFT optimization. The site Dy1A–O–Dy2A with the occupancy of 0.76 corresponds to the lowest energy conformer **1** found by DFT (Fig. S49). Optimization of the site Dy3A–O–Dy4A also converged to the conformer **1**. At the same time, optimization stated from the site Dy5A–O–Dy6A resulted in the conformer **3** (relative energy of 24 kJ/mol).

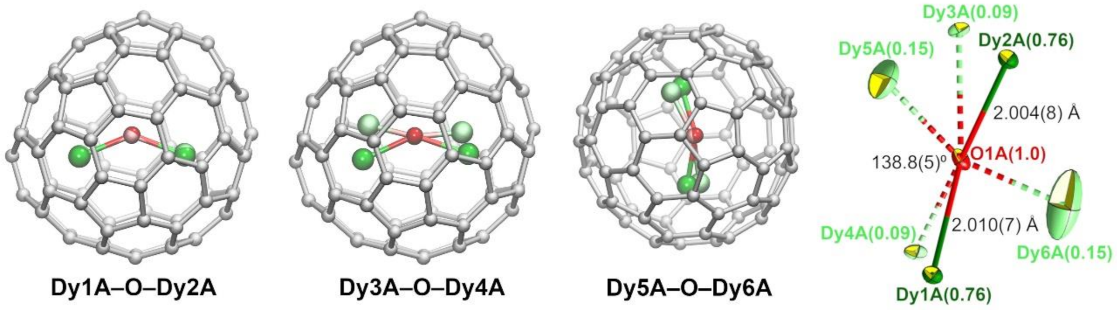

Figure S54. Comparison of the optimized Dy_2O positions (intense-colored atoms) with the starting coordinates obtained from X-ray structure (pale atoms). Dy sites and occupancies in the X-ray structures are shown on the right.

Correspondence between DFT-optimized conformes and X-ray structure of $Dy_2O@C_{3v}(8)-C_{82}$

For a reliable comparison of diffraction data and the structures of the DFT-optimized conformers, additional calculations were performed, in which coordinates of Dy sites from X-ray structures were used as starting coordinates for optimization. Dy sites in the crystal are divided into two groups, (Dy1, Dy3, Dy5) and (Dy2, Dy4, Dy6, Dy7, Dy8). All pairwise combinations gives 15 different structures used for DFT optimization. DFT optimization did not add new conformes, all optimized structures are among the conformers described in the Table S5. Interestingly, although the site Dy1 has the highest occupancy in the X-ray structures, there are no Dy atoms in the DFT-optimized conformers close to that site. We suggest that the group Dy3-Dy1-Dy5 describes the moving trajectory, rather than the static positions of metal atoms. The same holds for the second group of Dy sites.

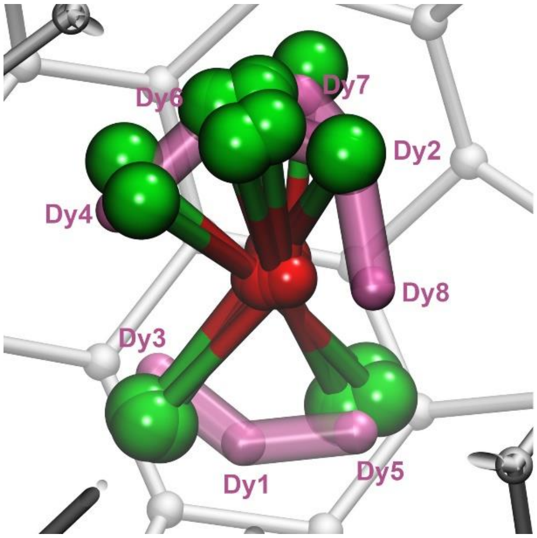

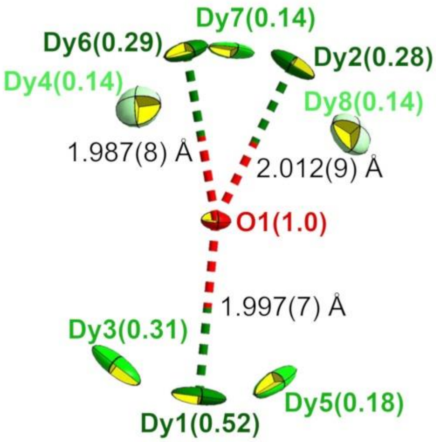

Figure S55. Left: Experimental Dy sites in $Dy_2O@C_{82}$-C_{3v} from X-ray diffraction data (shown as magenta spheres) overlaid with positions of Dy_2O cluster in DFT-optimized conformers. Right: Dy sites in the crystal structure of $Dy_2O@C_{82}$-C_{3v} with their occupancies.

Correspondence between DFT-optimized conformes and X-ray structure of $Dy_2O@C_{2v}(9)$-C_{82}

For $Dy_2O@C_{2v}(9)$-C_{82} we analyzed only positions with the highest occupancies, namely Dy1 (0.36), Dy1’ (0.36), Dy2 (0.28), Dy2’ (0.28), and Dy3 (0.16). Taking into account crystallographic symmetry plane, only three unique pairs of Dy atoms can be obtained from these sites: Dy1–O–Dy2, Dy1–O–Dy2’, and Dy1–O–Dy3. Optimization of these three structures showed that the combination Dy1–O–Dy2 (and Dy1’–O–Dy2’) does not correspond to an energy minimum (Dy atom moved considerably in the course of optimization), whereas Dy1–O–Dy2 and Dy1–O–Dy3 (and likewise Dy1’–O–Dy2 and Dy1’–O–Dy3) do correspond to the energy minima assigned as conformers **5** and **7**.

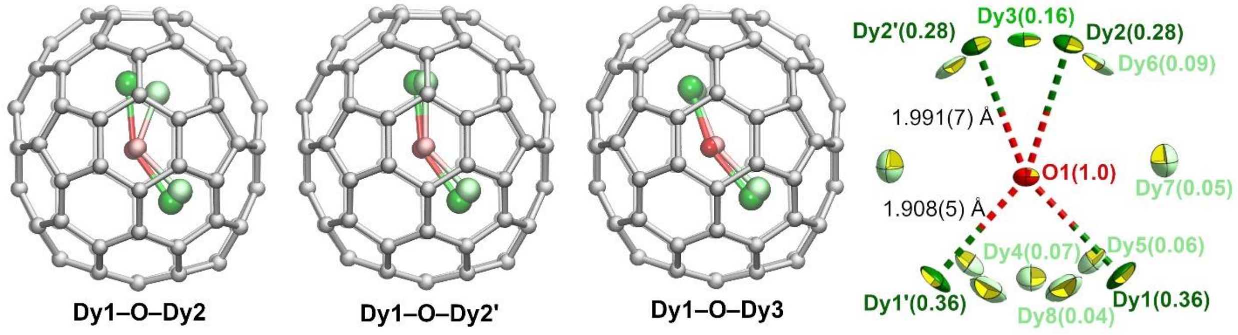

Figure S56. Comparison of the optimized Dy_2O positions (intense-colored atoms) with the starting coordinates obtained from X-ray structure (pale atoms). Dy sites and occupancies in the X-ray structures are shown on the right.

IR spectra

IR spectra of $Dy_2O@C_{82}$ samples drop-casted on KBr substrates were measured at room temperature with Vertex 80 FTIR spectrometer (Bruker) equipped with Hyperion microscope. The spectra of the three isomers of $Dy_2O@C_{82}$ are found to be strongly sensitive to the isomeric structure of the fullerene cage (Fig. S57). A proper description of the cluster dynamics is also important for the modelling of IR spectra. DFT calculations for different low energy conformers predict a noticeable variation in the spectra depending on the position of the metal-oxide cluster, and hence their possible coexistence should be taken into account. From the BOMD simulations, IR spectra can be obtained by Fourier transformation of the time evolution of the dipole moment. The contribution of different conformers is then included implicitly. Figure S57 shows that a good agreement is obtained between experimental and calculated spectra. Importantly, the simulations predict a considerable variation of the spectral pattern with the isomeric structure of the fullerene cage structure, which agrees well with experimental observations.

Of particular interest is the identification of the vibrations of the Dy_2O cluster. In the mid-IR range, DFT calculations reveal only one cluster vibration, the Dy−O antisymmetric stretching mode, which has a relatively high intensity. In the experimental spectra these vibrations can be assigned to medium-intensity absorption bands at 680–700 cm^{-1} (marked by arrows in Fig. S57). For comparison, analogous vibration in $Dy_2ScN@C_{80}$[98] is found at 740 cm^{-1}, and the Dy−C antisymmetric stretching mode in $Dy_2TiC@C_{80}$[18] occurs at 660 cm^{-1}.

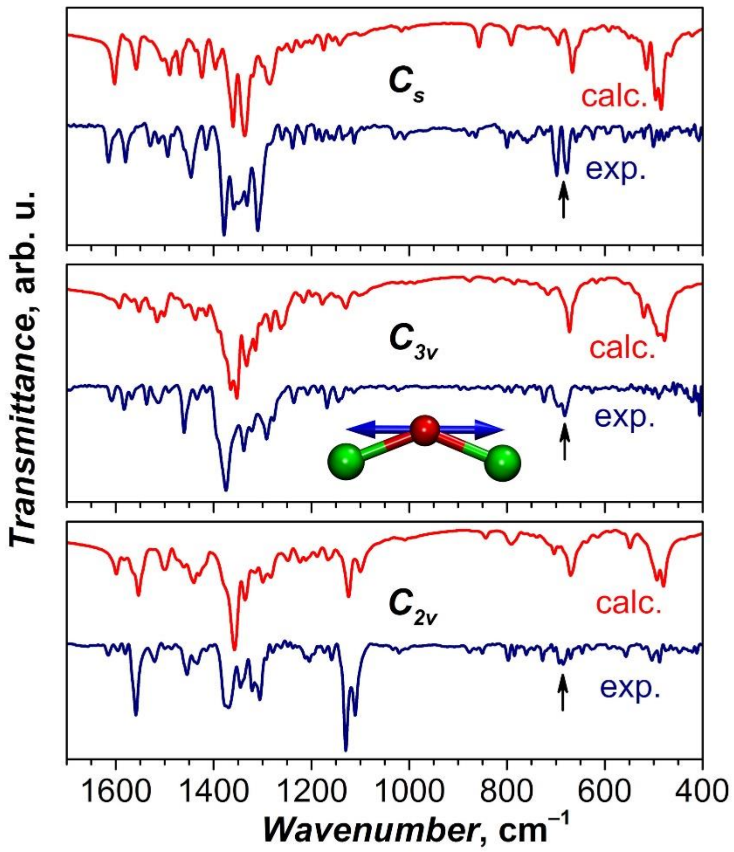

Figure S57. Experimental infrared spectra of $Dy_2O@C_{82}$ isomers (dark blue lines) compared to the spectra computed from the molecular dynamics simulations (red lines). Black arrows denote antisymmetric Dy–O stretching mode; vibrational displacements of the oxygen atoms in this mode are visualized as blue arrows in the inset: the oxygen atom is moving along the line parallel to the Dy···Dy axis so that one Dy–O bond is always shortened whereas another one is elongated at the same time.

Electronic structure and spectroscopic properties of $Dy_2O@C_{82}$ isomers

Vis−NIR absorption spectroscopy. The purified samples dissolved in toluene were characterized by Vis−NIR absorption spectroscopy, as shown in Fig. S58. The absorption spectrum of $Dy_2O@C_s(6)$-C_{82} exhibits strong absorptions at 503, 675, 753 , 838 and a broad and relatively weaker absorption centered around 1300 nm, while that of $Dy_2O@C_{2v}(9)$-C_{82} shows pronounced absorptions at 615, 864 and 937 nm, and $Dy_2O@C_{3v}(8)$-C_{82} shows two broad and weak absorptions at 730, 920 nm, respectively. The spectra of fullerenes are dominated by the π−π* transitions of the carbon cages and the cage symmetry.[1, 99] The absorption spectrum of $Dy_2O@C_s(6)$-C_{82} showed great resemblance to that of the previously reported $Sc_2O@C_s(6)$-C_{82},[63] indicating that they may share the same cage symmetry of $C_s(6)$-C_{82} and the electronic structure of $(M_2O)^{4+}@(C_s(6)\text{-}C_{82})^{4-}$ (M = Dy or Sc). Similarly, the absorption pattern and the characteristic peaks of $Dy_2O@C_{2v}(9)$-C_{82} and $Dy_2O@C_{3v}(8)$-C_{82} are almost identical to those of $Sc_2C_2@C_{2v}(9)$-C_{82}[100, 101] and $Sc_2O@C_{3v}(8)$-C_{82}[33], respectively, again indicating the resemblance of their cage symmetry and electronic structure.

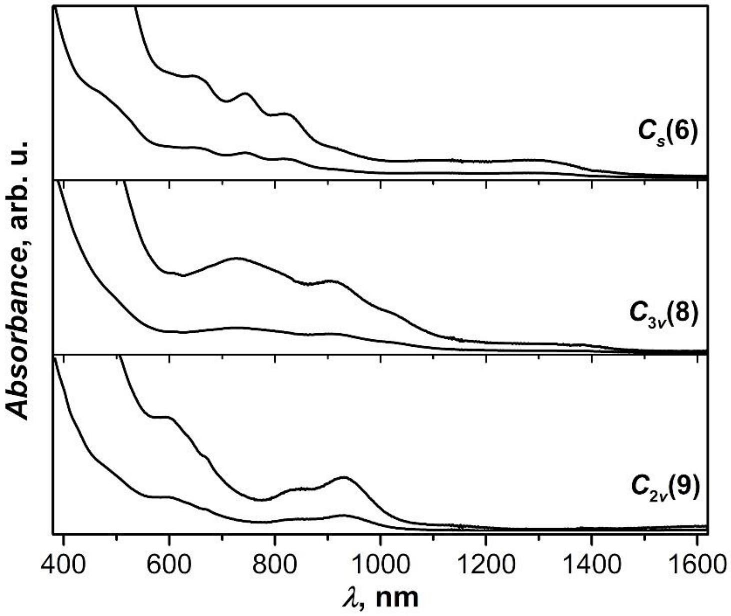

Figure S58. Vis-NIR absorption spectra of three $Dy_2O@C_{82}$ isomers measured at room temperature in toluene solution.

Electrochemical Studies. The electrochemical properties of $Dy_2O@C_{82}$ ($C_s(6)$, $C_{3v}(8)$, $C_{2v}(9)$) were investigated by cyclic voltammetry (CV). The characteristic redox potentials are summarized in Table S7, along with those of analogous C_{82}-based endohedral fullerenes which contain the valence isoelectronic cluster Sc_2X (X = O or C_2).[33, 63, 100] The cyclic voltammogram of $Dy_2O@C_s(6)$-C_{82} presents four reversible reduction peaks at −0.75, −1.17, −1.86, −2.24 V, respectively (Fig. S59a). For the oxidative processes, $Dy_2O@C_s(6)$-C_{82} exhibits two reversible oxidation steps at +0.19, +0.42 V, and one irreversible oxidation step at +0.95 V. The first and second oxidation peaks of $Dy_2O@C_s(6)$-C_{82} are shifted negatively from those of $Sc_2O@C_s(6)$-C_{82},[63] while the first and second reduction peaks of $Dy_2O@C_s(6)$-C_{82} are shifted positively from those of $Sc_2O@C_s(6)$-C_{82}. Thus, the resulting the electrochemical behavior electrochemical gap (0.94 V) is much smaller than that of $Sc_2O@C_s(6)$-C_{82} (1.31 V). Likewise, for $Dy_2O@C_{3v}(8)$-C_{82}, the first oxidation peak shifted negatively and the first reduction peak is shifted positively from those of $Sc_2O@C_{3v}(8)$-C_{82}.[33] On the other hand, the overall redox behavior of the three isomer of $Dy_2O@C_{82}$ varies from each other. The first reduction peak changes slightly from $Dy_2O@C_{2v}(9)$-C_{82} (−0.69 V) to $Dy_2O@C_s(6)$-C_{82} (−0.75 V) to $Dy_2O@C_{3v}(8)$-C_{82} (−0.77 V), and the first oxidation peak changes more obviously from $Dy_2O@C_{2v}(9)$-C_{82} (0.23 V) to $Dy_2O@C_s(6)$-C_{82} (0.19 V) to $Dy_2O@C_{3v}(8)$-C_{82} (0.43 V), and the resulting electrochemical gap of the three compounds vary from 0.92 to 0.94 to 1.20 V. DFT calculations show that frontier orbitals of all three isomers are localized on the fullerene with negligible contribution from the Dy_2O cluster (Fig. S59b). These results indicate that the change of cage symmetry can exert a noticeable influence on their electrochemical behavior as well as the electronic structures.

Table S7. Redox Potentials (V vs Fc/Fc$^+$) of $Dy_2O@C_{82}$ ($C_s(6)$, $C_{3v}(8)$, $C_{2v}(9)$) obtained in (n-Bu_4)NPF_6/*ortho*-dichlorobenzene with Ferrocene (Fc) as the Internal Standard

compound	$E^{3+/2+}$	$E^{2+/+}$	$E^{+/0}$	$E^{0/-}$	$E^{-/2-}$	$E^{2-/3-}$	$E^{3-/4-}$	$E_{gap,ec}$	Ref.
$Dy_2O@C_s(6)$-C_{82}	+0.95^b	+0.42^a	+0.19^a	−0.75^a	−1.17^a	−1.86^a	−2.24^a	0.94	t.w.
$Sc_2O@C_s(6)$-C_{82}		+0.72^a	+0.35^a	−0.96^a	−1.28^a	−1.74^b		1.31	32
$Dy_2O@C_{3v}(8)$-C_{82}		+0.91^b	+0.43^a	−0.77^b	−1.20^a	−1.78^a	−2.08^a	1.20	t.w.
$Sc_2O@C_{3v}(8)$-C_{82}		+1.09^b	+0.54^a	−1.17^b	−1.44^b	−1.55^b	−1.78^b	1.71	33
$Dy_2O@C_{2v}(9)$-C_{82}		+0.68^a	+0.23^a	−0.69^a	−0.98^a	−1.81^b	−2.12^b	0.92	t.w.
$Sc_2C_2@C_{2v}(9)$-C_{82}		+0.67^a	+0.25^a	−0.74^a	−0.96^b			0.99	100

^{a}Half-wave potential in volts (reversible redox process). ^{b}Peak potential in volts (irreversible redox process).

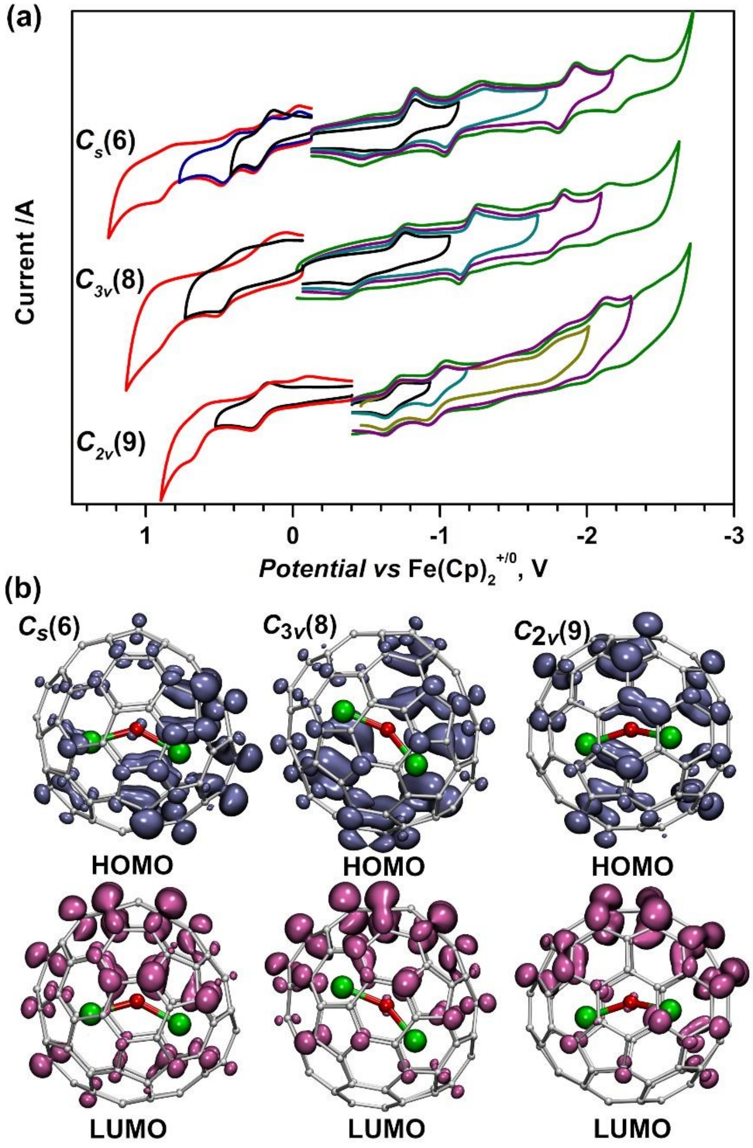

Figure S59. (a) Cyclic voltammetry of $Dy_2O@C_{82}$ isomers measured in $(n\text{-}Bu_4)NPF_6$/*ortho*-dichlorobenzene solution, potential sweep rate 100 mV/s. (b) Orbital density of the Kohn-Sham HOMO (upper row) and LUMO (lower row) of $Dy_2O@C_{82}$ isomers.

Ab initio computed LF splitting and transition probabilities

Table S8. *Ab initio* computed ligand-field splitting energies for Dy ions in $Dy_2O@C_{82}$-C_s, **Conf. 1**

KD	**Dy-1** (cm^{-1})	**Dy-2** (cm^{-1})
1	0.0	0.0
2	515.3	439.3
3	835.6	821.6
4	1048.0	1109.3
5	1212.2	1301.9
6	1336.0	1390.9
7	1402.7	1422.5
8	1490.0	1473.2
d(Dy–N), Å	2.0308	2.0447
	**KD-1**	**KD-1**
g_x	0.000099135	0.000235430
g_y	0.000145333	0.000263369
g_z	19.885932550	19.866277107

Geometrical angle Dy–O–Dy: 136.1°

Angle between axes of KD-1 states: 133.8° (46.2°)

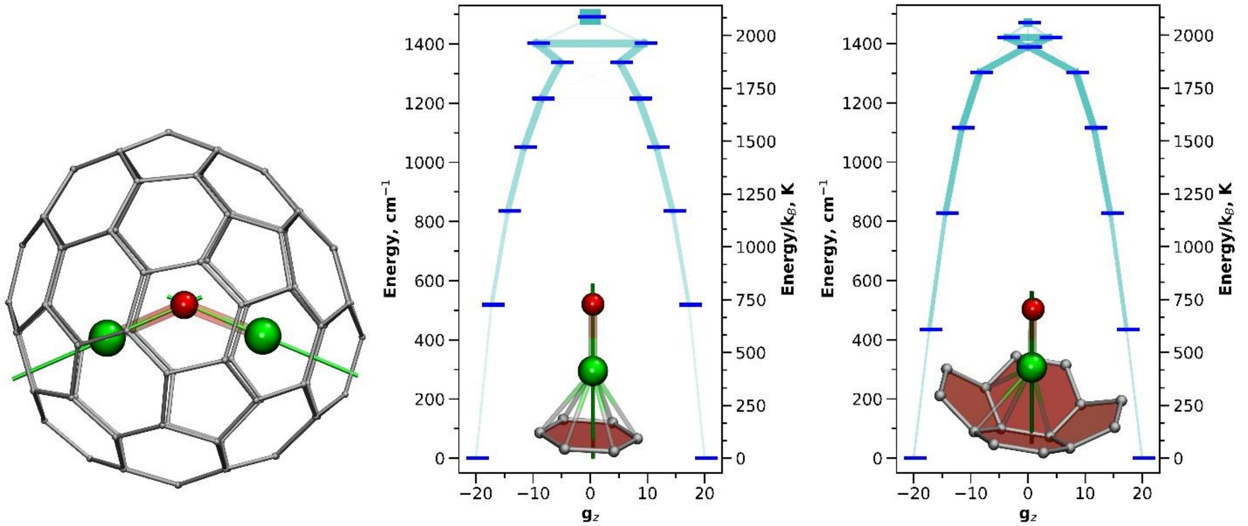

Figure S60. Left: DFT-optimized molecular structure of $Dy_2O@C_{82}$-C_s, **Conf. 1,** showing quantization axes for each Dy ion (green lines).Middle and right: *Ab initio* computed ligand-field states (thick blue dashes) and transition probabilities between them (light blue lines, the thicker the line – the higher the transition probability) for two Dy ions. Also shown are Dy-cage coordination sites and quantization axes for each Dy ions (dark green lines). Dy – green, O – red, C – gray, Dy–C distances less than 2.60 Å are shown as bonds.

Table S9. *Ab initio* computed ligand-field splitting energies for Dy ions in $Dy_2O@C_{82}$-C_{3v}, **Conf. 1**

KD	**Dy-1** (cm^{-1})	**Dy-2** (cm^{-1})
1	0.0	0.0
2	401.0	410.6
3	801.8	773.8
4	1116.4	1061.7
5	1271.9	1211.3
6	1338.5	1263.9
7	1396.0	1336.3
8	1435.6	1381.3
d(Dy–N), Å	2.0148	2.0355
	**KD-1**	**KD-1**
g_x	0.000091072	0.000137526
g_y	0.000096617	0.000170688
g_z	19.870580651	19.887397896

Geometrical angle Dy–O–Dy: 145.2°
Angle between axes of KD-1 states: 142.5° (37.5°)

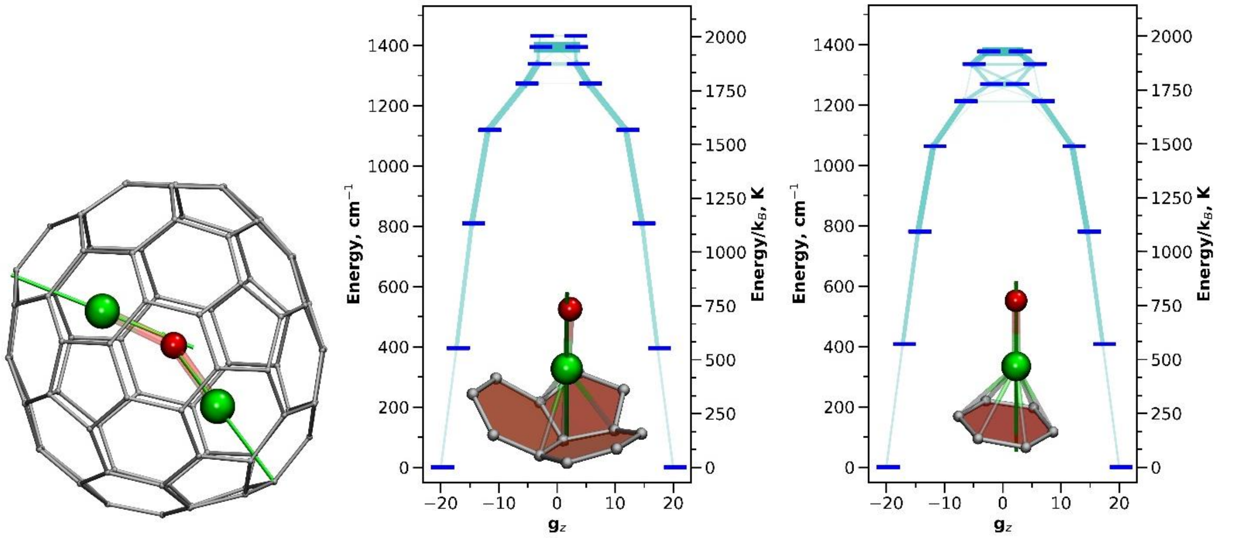

Figure S61. Left: DFT-optimized molecular structure of $Dy_2O@C_{82}$-C_{3v}, **Conf. 1**, showing quantization axes for each Dy ion (green lines).Middle and right: *Ab initio* computed ligand-field states (thick blue dashes) and transition probabilities between them (light blue lines, the thicker the line – the higher the transition probability) for two Dy ions. Also shown are Dy-cage coordination sites and quantization axes for each Dy ions (dark green lines). Dy – green, O – red, C – gray, Dy–C distances less than 2.60 Å are shown as bonds.

Table S10. *Ab initio* computed ligand-field splitting energies for Dy ions in $Dy_2O@C_{82}$-C_{3v}, **Conf. 2**

KD	**Dy-1** (cm^{-1})	**Dy-2** (cm^{-1})
1	0.0	0.0
2	396.2	495.0
3	799.2	823.5
4	1117.7	1052.8
5	1273.9	1188.6
6	1332.0	1292.8
7	1387.1	1376.3
8	1441.2	1429.5
d(Dy–N), Å	2.0164	2.0439
	**KD-1**	**KD-1**
g_x	0.000070604	0.000094600
g_y	0.000071798	0.000136028
g_z	19.871781953	19.894908421

Geometrical angle Dy–O–Dy: 139.1°

Angle between axes of KD-1 states: 134.8° (45.2°)

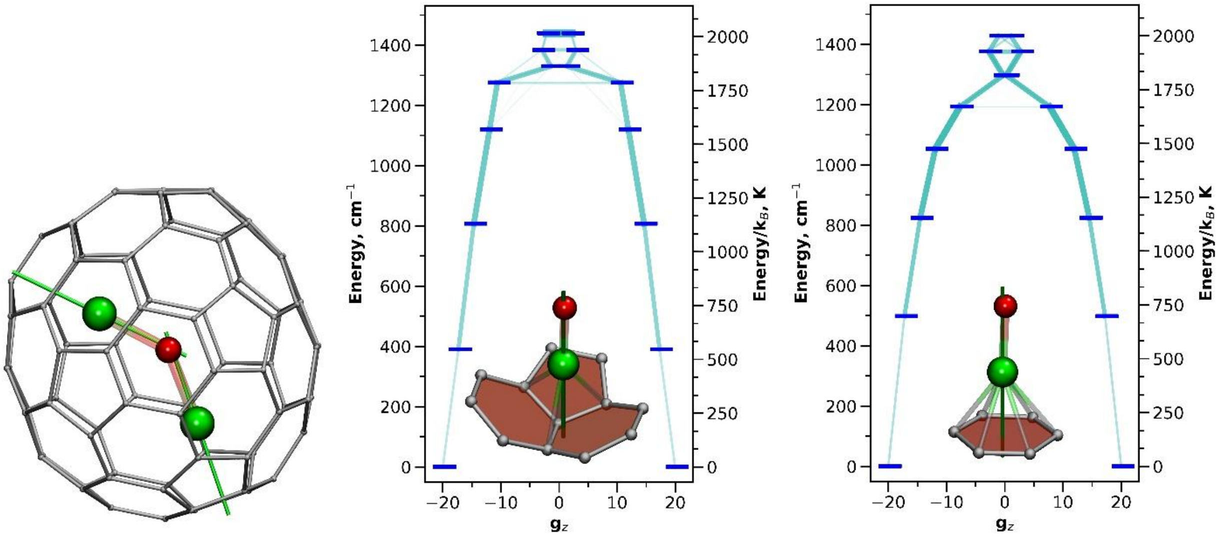

Figure S62. Left: DFT-optimized molecular structure of $Dy_2O@C_{82}$- C_{3v}**, Conf. 2**, showing quantization axes for each Dy ion (green lines).Middle and right: *Ab initio* computed ligand-field states (thick blue dashes) and transition probabilities between them (light blue lines, the thicker the line – the higher the transition probability) for two Dy ions. Also shown are Dy-cage coordination sites and quantization axes for each Dy ions (dark green lines). Dy – green, O – red, C – gray, Dy–C distances less than 2.60 Å are shown as bonds.

Table S11. *Ab initio* computed ligand-field splitting energies for Dy ions in $Dy_2O@C_{82}$-C_{3v}, **Conf. 3**

KD	**Dy-1** (cm^{-1})	**Dy-2** (cm^{-1})
1	0.0	0.0
2	409.7	457.5
3	818.3	805.3
4	1135.1	1063.4
5	1295.4	1197.6
6	1373.3	1283.6
7	1418.7	1369.4
8	1448.6	1414.6
d(Dy–N), Å	2.0244	2.0335
	**KD-1**	**KD-1**
g_x	0.000058820	0.000042060
g_y	0.000063959	0.000056442
g_z	19.878519991	19.887782621

Geometrical angle Dy–O–Dy: 133.3°
Angle between axes of KD-1 states: 131.2° (48.8°)

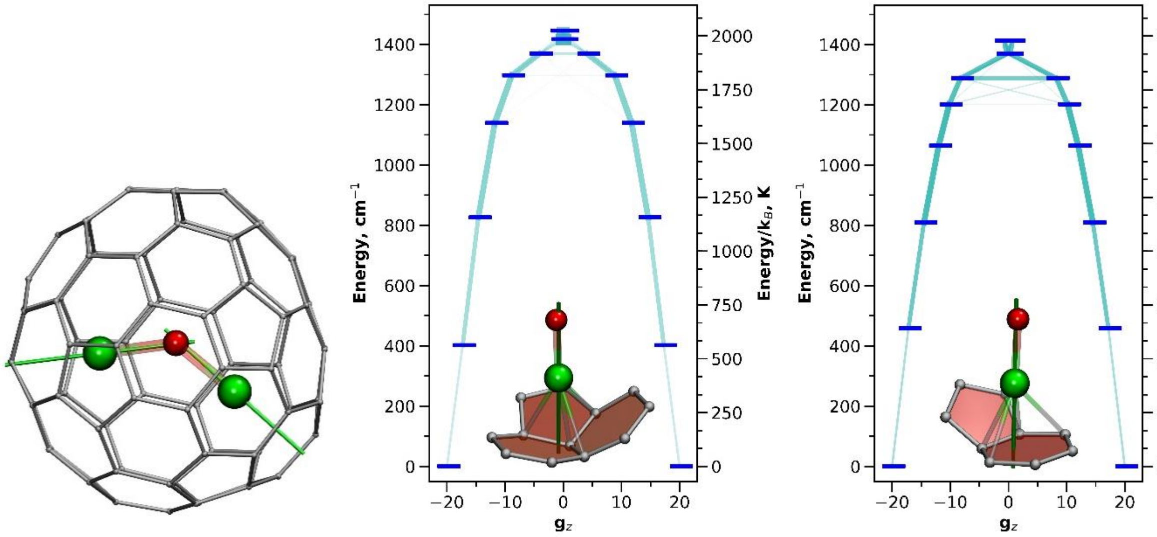

Figure S63. Left: DFT-optimized molecular structure of $Dy_2O@C_{82}$- C_{3v}, **Conf. 3**, showing quantization axes for each Dy ion (green lines).Middle and right: *Ab initio* computed ligand-field states (thick blue dashes) and transition probabilities between them (light blue lines, the thicker the line – the higher the transition probability) for two Dy ions. Also shown are Dy-cage coordination sites and quantization axes for each Dy ions (dark green lines). Dy – green, O – red, C – gray, Dy–C distances less than 2.60 Å are shown as bonds.

Table S12. *Ab initio* computed ligand-field splitting energies for Dy ions in $Dy_2O@C_{82}$-C_{3v}, **Conf. 4**

KD	**Dy-1** (cm^{-1})	**Dy-2** (cm^{-1})
1	0.0	0.0
2	507.8	465.4
3	880.7	800.4
4	1138.2	1048.9
5	1299.1	1200.1
6	1382.6	1291.6
7	1427.8	1366.8
8	1469.7	1411.8
d(Dy–N), Å	2.0087	2.0656
	**KD-1**	**KD-1**
g_x	0.000031200	0.000031539
g_y	0.000046972	0.000051207
g_z	19.894141062	19.896558139

Geometrical angle Dy–O–Dy: 172.7°
Angle between axes of KD-1 states: 169.2° (10.8°)

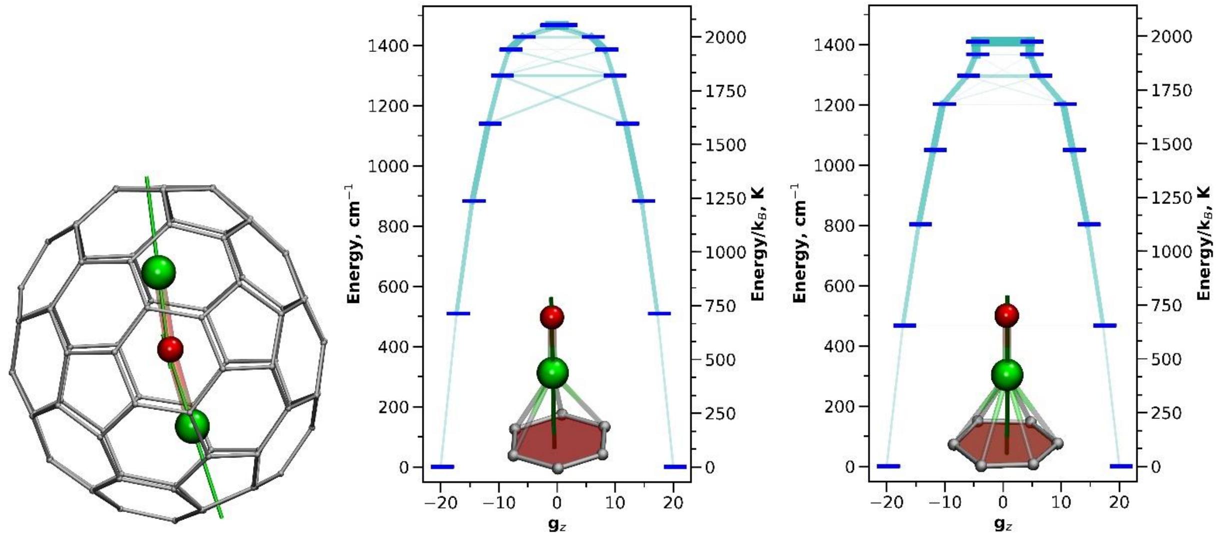

Figure S64. Left: DFT-optimized molecular structure of $Dy_2O@C_{82}$-C_{3v}, **Conf. 4**, showing quantization axes for each Dy ion (green lines).Middle and right: *Ab initio* computed ligand-field states (thick blue dashes) and transition probabilities between them (light blue lines, the thicker the line – the higher the transition probability) for two Dy ions. Also shown are Dy-cage coordination sites and quantization axes for each Dy ions (dark green lines). Dy – green, O – red, C – gray, Dy–C distances less than 2.60 Å are shown as bonds.

Table S13. *Ab initio* computed ligand-field splitting energies for Dy ions in $Dy_2O@C_{82}$-C_{3v}, **Conf. 5**

KD	**Dy-1** (cm^{-1})
1	0.0
2	387.1
3	758.3
4	1050.9
5	1222.5
6	1286.9
7	1315.7
8	1383.8
d(Dy–N), Å	2.0296
	**KD-1**
g_x	0.000317913
g_y	0.000365663
g_z	19.878468105

Geometrical angle Dy–O–Dy: 132.7°

Angle between axes of KD-1 states: 127.3° (52.7°)

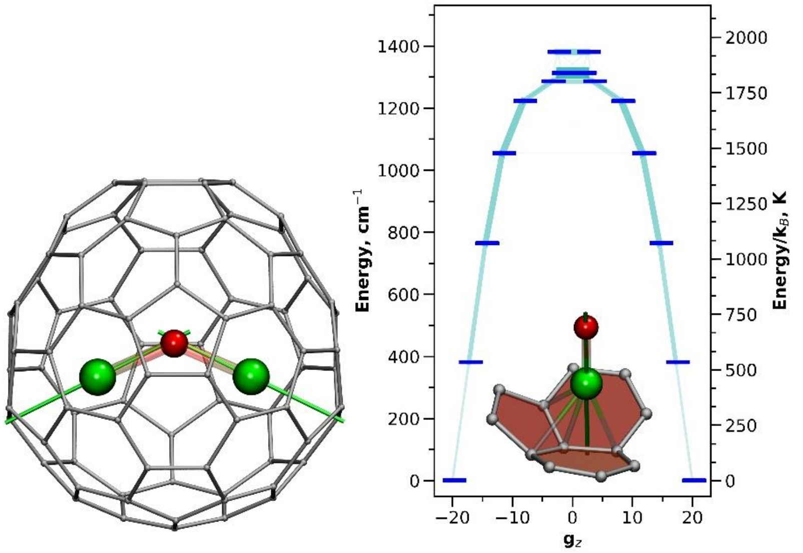

Figure S65. Left: DFT-optimized molecular structure of $Dy_2O@C_{82}$- C_{3v}, **Conf. 5**, showing quantization axes for each Dy ion (green lines). Right: *Ab initio* computed ligand-field states (thick blue dashes) and transition probabilities between them (light blue lines, the thicker the line – the higher the transition probability) for Dy ions (two Dy ions are equivalent in this conformer). Also shown is Dy-cage coordination site and quantization axes for a Dy ion (dark green lines). Dy – green, O – red, C – gray, Dy–C distances less than 2.60 Å are shown as bonds.

Table S14. *Ab initio* computed ligand-field splitting energies for Dy ions in $Dy_2O@C_{82}$-C_{2v}, **Conf. 1**

KD	**Dy-1** (cm^{-1})	**Dy-2** (cm^{-1})
1	0.0	0.0
2	440.5	434.6
3	803.1	827.7
4	1075.7	1130.9
5	1208.9	1311.2
6	1284.2	1390.7
7	1362.7	1418.2
8	1451.0	1469.8
d(Dy–N), Å	2.0387	2.0256
	**KD-1**	**KD-1**
g_x	0.000089682	0.000033306
g_y	0.000117453	0.000043130
g_z	19.877587515	19.881712225

Geometrical angle Dy–O–Dy: 138.9°

Angle between axes of KD-1 states: 136.2° (43.8°)

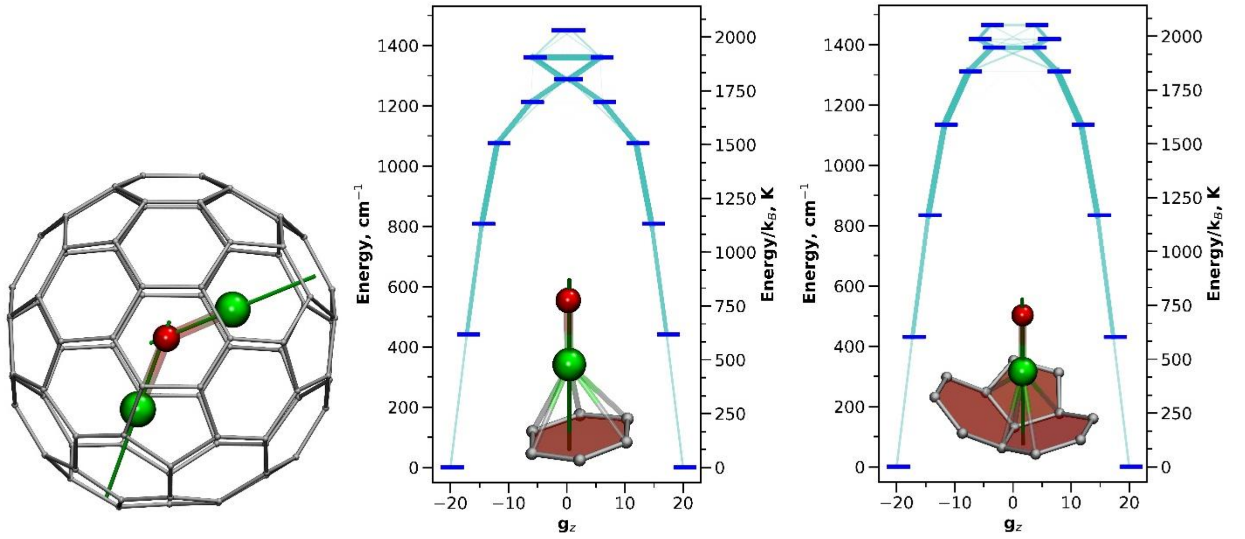

Figure S66. Left: DFT-optimized molecular structure of $Dy_2O@C_{82}$-C_{2v}, **Conf. 1**, showing quantization axes for each Dy ion (green lines).Middle and right: *Ab initio* computed ligand-field states (thick blue dashes) and transition probabilities between them (light blue lines, the thicker the line – the higher the transition probability) for two Dy ions. Also shown are Dy-cage coordination sites and quantization axes for each Dy ions (dark green lines). Dy – green, O – red, C – gray, Dy–C distances less than 2.60 Å are shown as bonds.

Table S15. *Ab initio* computed ligand-field splitting energies for Dy ions in $Dy_2O@C_{82}$-C_{2v}, **Conf. 2**

KD	**Dy-1** (cm^{-1})	**Dy-2** (cm^{-1})
1	0.0	0.0
2	386.6	403.4
3	796.1	808.2
4	1113.6	1125.7
5	1288.9	1297.3
**6**	1335.8	1373.6
7	1364.0	1406.7
8	1423.0	1457.5
d(Dy–N), Å	2.0327	2.0252
	**KD-1**	**KD-1**
g_x	0.000093768	0.000053503
g_y	0.000113918	0.000061368
g_z	19.862240855	19.872316938

Geometrical angle Dy–O–Dy: 145.9°

Angle between axes of KD-1 states: 139.9° (40.1°)

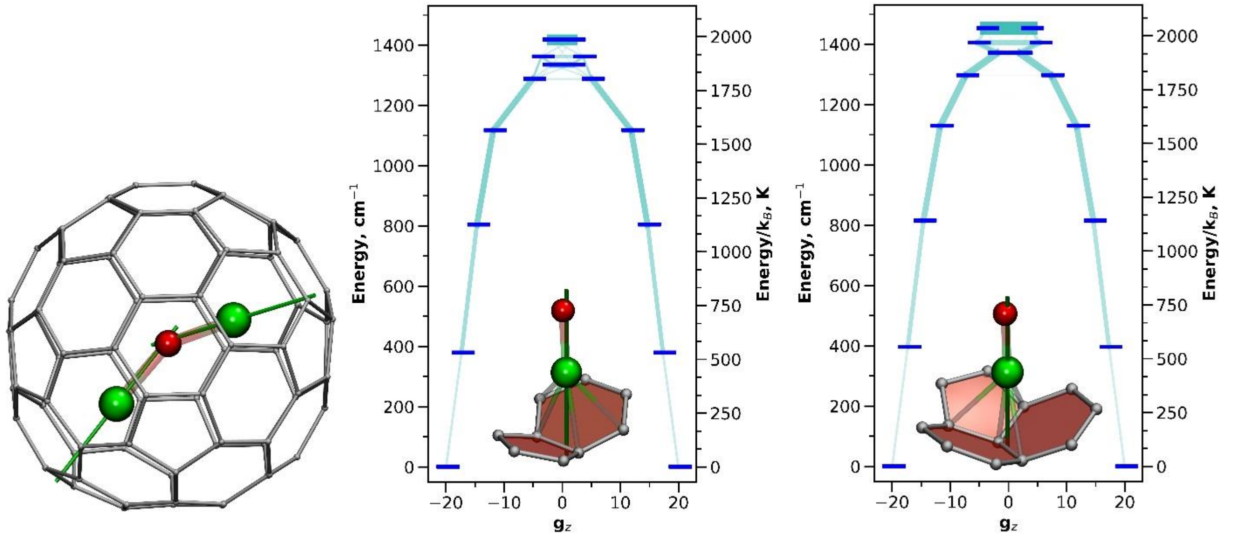

Figure S67. Left: DFT-optimized molecular structure of $Dy_2O@C_{82}$-C_{2v}, **Conf. 2**, showing quantization axes for each Dy ion (green lines).Middle and right: *Ab initio* computed ligand-field states (thick blue dashes) and transition probabilities between them (light blue lines, the thicker the line – the higher the transition probability) for two Dy ions. Also shown are Dy-cage coordination sites and quantization axes for each Dy ions (dark green lines). Dy – green, O – red, C – gray, Dy–C distances less than 2.60 Å are shown as bonds.

Table S16. *Ab initio* computed ligand-field splitting energies for Dy ions in $Dy_2O@C_{82}$-C_{2v}, **Conf. 3**

KD	**Dy-1** (cm^{-1})	**Dy-2** (cm^{-1})
1	0.0	0.0
2	464.0	445.6
3	816.9	834.1
4	1085.8	1122.6
5	1240.4	1266.5
**6**	1315.5	1322.8
7	1395.5	1398.6
8	1446.4	1525.4
d(Dy–N), Å	2.0311	2.0260
	**KD-1**	**KD-1**
g_x	0.000047498	0.000008240
g_y	0.000059732	0.000011873
g_z	19.889600533	19.865234636

Geometrical angle Dy–O–Dy: 138.3°
Angle between axes of KD-1 states: 136.6° (43.4°)

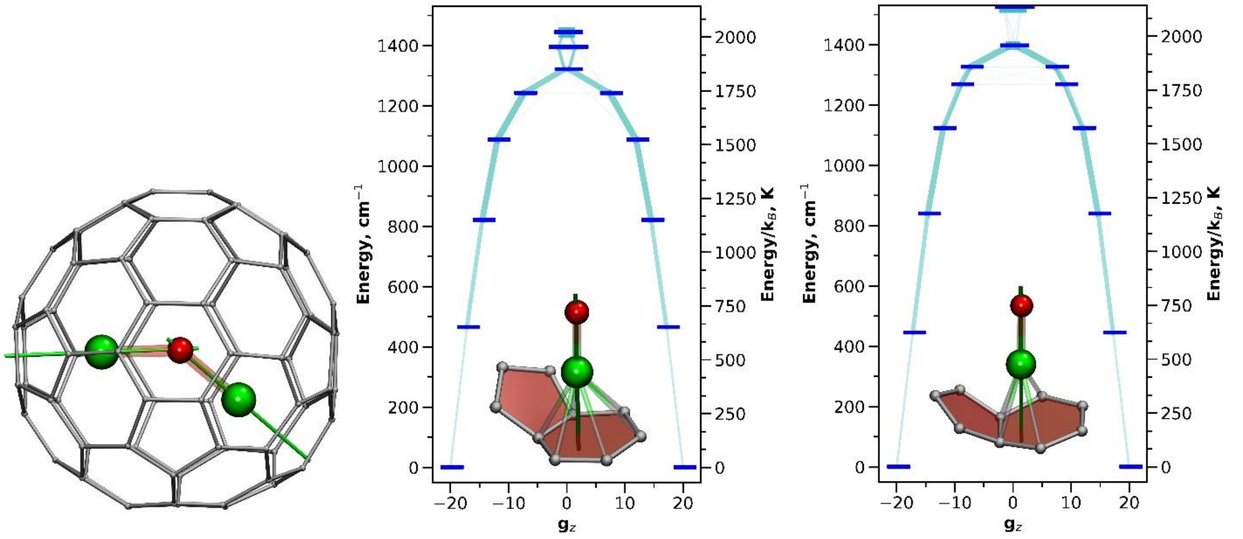

Figure S68. Left: DFT-optimized molecular structure of $Dy_2O@C_{82}$-C_{2v}, **Conf. 3,** showing quantization axes for each Dy ion (green lines).Middle and right: *Ab initio* computed ligand-field states (thick blue dashes) and transition probabilities between them (light blue lines, the thicker the line – the higher the transition probability) for two Dy ions. Also shown are Dy-cage coordination sites and quantization axes for each Dy ions (dark green lines). Dy – green, O – red, C – gray, Dy–C distances less than 2.60 Å are shown as bonds.

Table S17. *Ab initio* computed ligand-field splitting energies for Dy ions in $Dy_2O@C_{82}$-C_{2v}, **Conf. 4**

KD	**Dy-1** (cm^{-1})
1	0.0
2	419.8
3	823.4
4	1125.3
5	1307.7
6	1348.5
7	1405.6
8	1451.9
d(Dy–N), Å	2.0316
	**KD-1**
g_x	0.000047979
g_y	0.000052424
g_z	19.869562390

Geometrical angle Dy–O–Dy: 137.5°
Angle between axes of KD-1 states: 136.1° (43.9°)

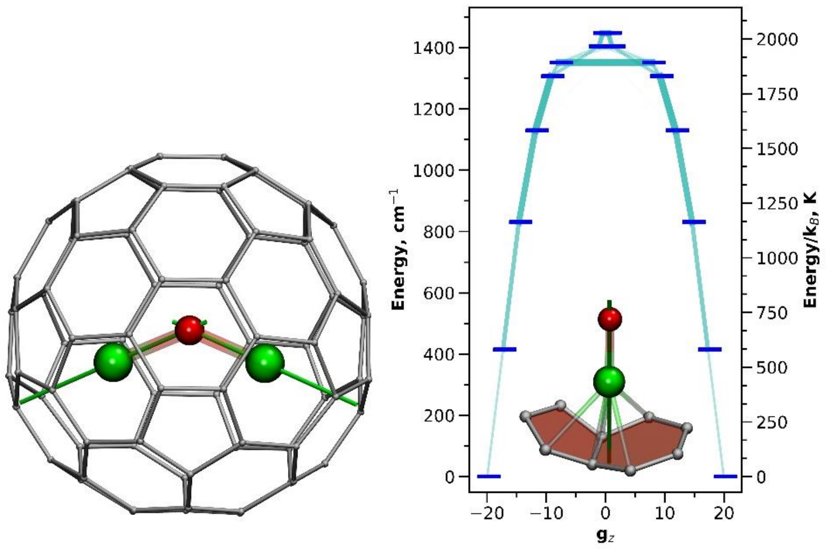

Figure S69. Left: DFT-optimized molecular structure of $Dy_2O@C_{82}$- C_{3v}, **Conf. 4**, showing quantization axes for each Dy ion (green lines). Right: *Ab initio* computed ligand-field states (thick blue dashes) and transition probabilities between them (light blue lines, the thicker the line – the higher the transition probability) for Dy ions (two Dy ions are equivalent in this conformer). Also shown is Dy-cage coordination site and quantization axes for a Dy ion (dark green lines). Dy – green, O – red, C – gray, Dy–C distances less than 2.60 Å are shown as bonds.

Table S18. *Ab initio* computed ligand-field splitting energies for Dy ions in $Dy_2O@C_{82}$-C_{2v}, **Conf. 5**

KD	**Dy-1** (cm^{-1})	**Dy-2** (cm^{-1})
1	0.0	0.0
2	402.1	407.8
3	813.6	770.5
4	1135.1	1058.9
5	1299.9	1204.9
6	1349.7	1256.8
7	1411.6	1334.6
8	1451.5	1382.3
d(Dy–N), Å	2.0044	2.0402
	**KD-1**	**KD-1**
g_x	0.000167726	0.000138674
g_y	0.000181909	0.000167775
g_z	19.847753420	19.903738694

Geometrical angle Dy–O–Dy: 154.8°

Angle between axes of KD-1 states: 155.2° (24.8°)

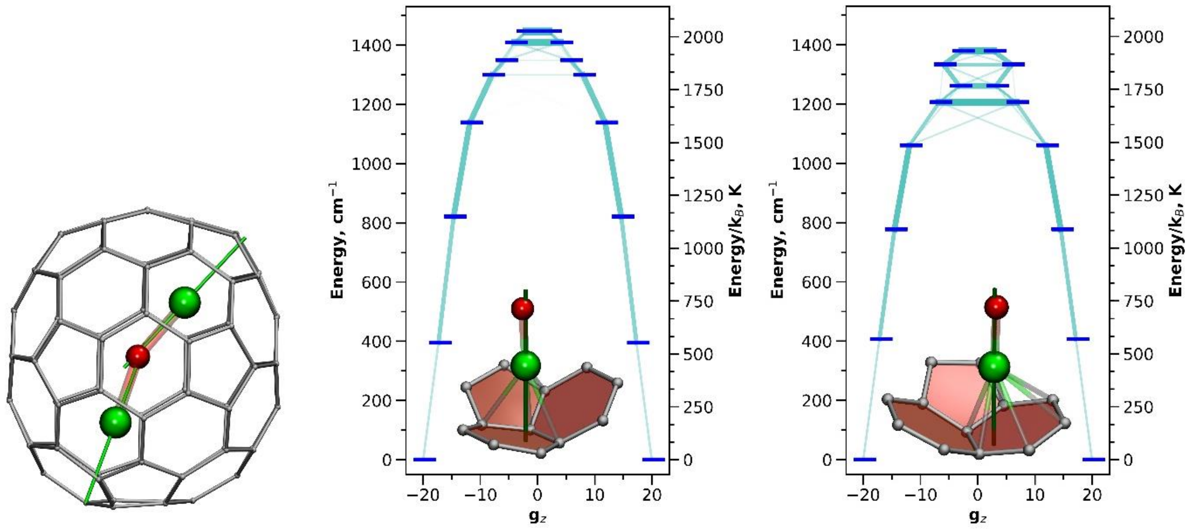

Figure S70 Left: DFT-optimized molecular structure of $Dy_2O@C_{82}$-C_{2v}, **Conf. 5,** showing quantization axes for each Dy ion (green lines).Middle and right: *Ab initio* computed ligand-field states (thick blue dashes) and transition probabilities between them (light blue lines, the thicker the line – the higher the transition probability) for two Dy ions. Also shown are Dy-cage coordination sites and quantization axes for each Dy ions (dark green lines). Dy – green, O – red, C – gray, Dy–C distances less than 2.60 Å are shown as bonds.

Table S19. *Ab initio* computed ligand-field splitting energies for Dy ions in $Dy_2O@C_{82}$-C_{2v}, **Conf. 6**

KD	**Dy-1** (cm^{-1})
1	0.0
2	419.2
3	787.9
4	1076.8
5	1243.1
**6**	1314.2
7	1368.4
8	1424.3
d(Dy–N), Å	2.0294
	**KD-1**
g_x	0.000068191
g_y	0.000076004
g_z	19.887159848

Geometrical angle Dy–O–Dy: 150.1°

Angle between axes of KD-1 states: 153.1° (26.9°)

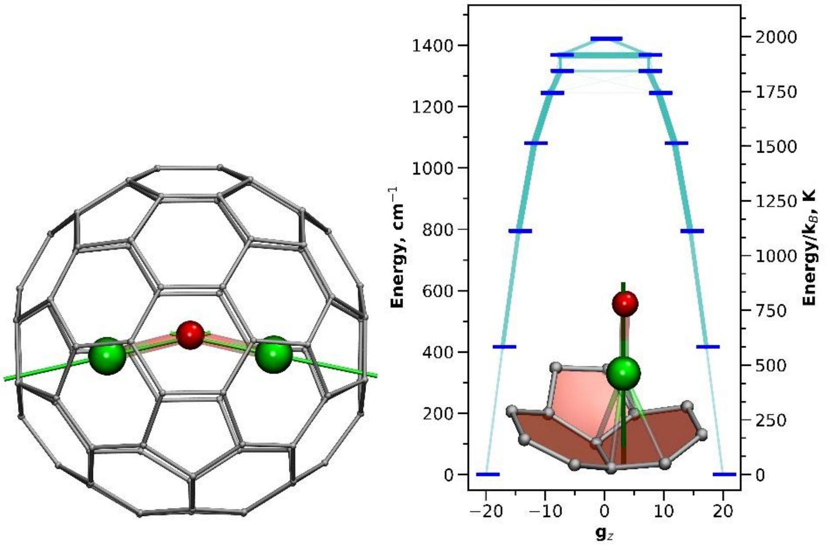

Figure S71. Left: DFT-optimized molecular structure of $Dy_2O@C_{82}$- C_{3v}, **Conf. 6**, showing quantization axes for each Dy ion (green lines). Right: *Ab initio* computed ligand-field states (thick blue dashes) and transition probabilities between them (light blue lines, the thicker the line – the higher the transition probability) for Dy ions (two Dy ions are equivalent in this conformer). Also shown is Dy-cage coordination site and quantization axes for a Dy ion (dark green lines). Dy – green, O – red, C – gray, Dy–C distances less than 2.60 Å are shown as bonds.

Table S20. *Ab initio* computed ligand-field splitting energies for Dy ions in $Dy_2O@C_{82}$-C_{2v}, **Conf. 7**

KD	**Dy-1** (cm^{-1})	**Dy-2** (cm^{-1})
1	0.0	0.0
2	434.2	409.5
3	788.9	819.2
4	1064.9	1142.5
5	1216.4	1304.7
6	1277.7	1348.7
7	1344.5	1409.7
8	1398.8	1450.7
d(Dy–N), Å	2.0466	2.0098
	**KD-1**	**KD-1**
g_x	0.000041903	0.000223388
g_y	0.000057787	0.000236571
g_z	19.888255494	19.877273704

Geometrical angle Dy–O–Dy: 148.6°

Angle between axes of KD-1 states: 143.7° (36.3°)

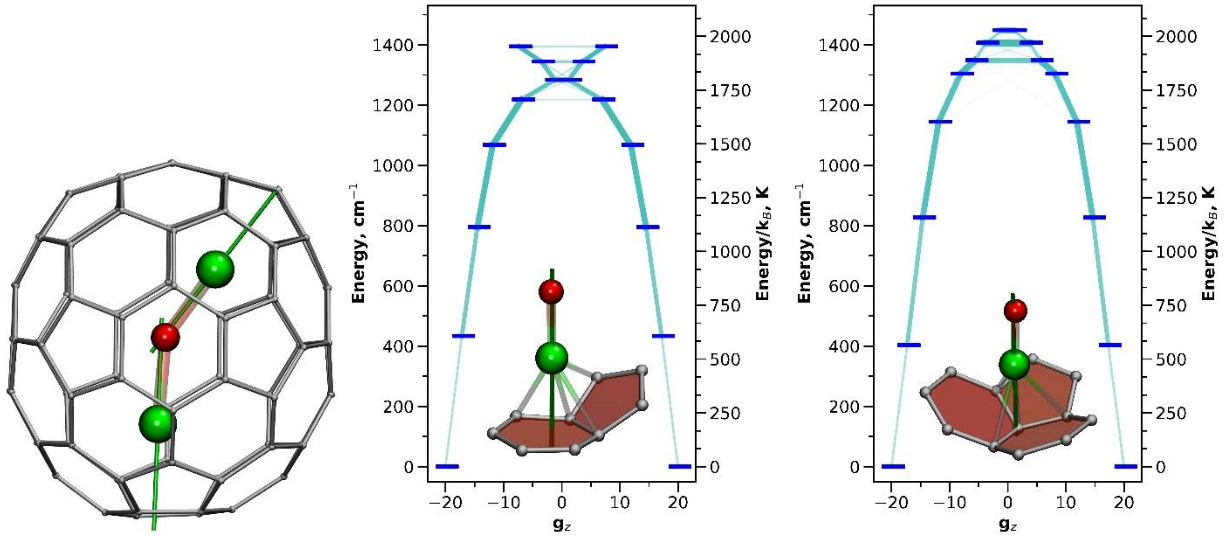

Figure S72. Left: DFT-optimized molecular structure of $Dy_2O@C_{82}$-C_{2v}, **Conf. 7,** showing quantization axes for each Dy ion (green lines).Middle and right: *Ab initio* computed ligand-field states (thick blue dashes) and transition probabilities between them (light blue lines, the thicker the line – the higher the transition probability) for two Dy ions. Also shown are Dy-cage coordination sites and quantization axes for each Dy ions (dark green lines). Dy – green, O – red, C – gray, Dy–C distances less than 2.60 Å are shown as bonds.

Table S21. *Ab initio* computed ligand-field splitting energies for Dy ions in $Dy_2O@C_{82}$-C_{2v}, **Conf. 8**

KD	**Dy-1** (cm^{-1})
1	0.0
2	539.5
3	945.0
4	1235.8
5	1392.6
6	1466.4
7	1544.9
8	1645.5
d(Dy–N), Å	2.0244
	**KD-1**
g_x	0.000015361
g_y	0.000018687
g_z	19.898441787

Geometrical angle Dy–O–Dy: 125.7°
Angle between axes of KD-1 states: 111.9° (68.1°)

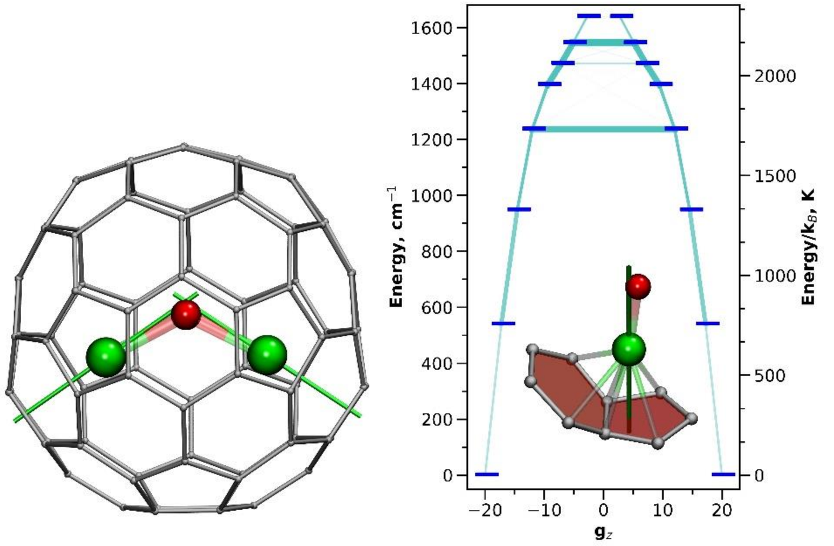

Figure S73. Left: DFT-optimized molecular structure of $Dy_2O@C_{82}$-C_{3v}, **Conf. 8**, showing quantization axes for each Dy ion (green lines). Right: *Ab initio* computed ligand-field states (thick blue dashes) and transition probabilities between them (light blue lines, the thicker the line – the higher the transition probability) for Dy ions (two Dy ions are equivalent in this conformer). Also shown is Dy-cage coordination site and quantization axes for a Dy ion (dark green lines). Dy – green, O – red, C – gray, Dy–C distances less than 2.60 Å are shown as bonds.

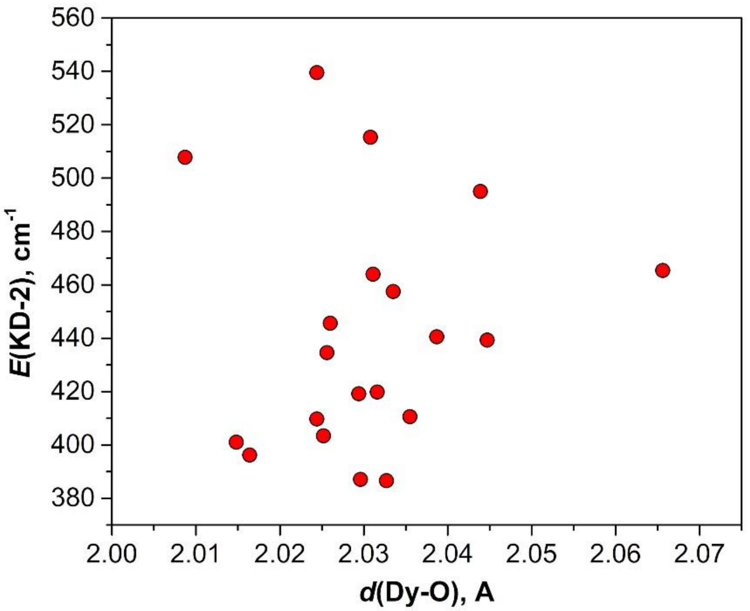

Figure S74 The energy of the first excited Kramers doublet (KD-2) in DFT-optimized $Dy_2O@C_{82}$ conformers plotted versus the Dy–O bond lengths.

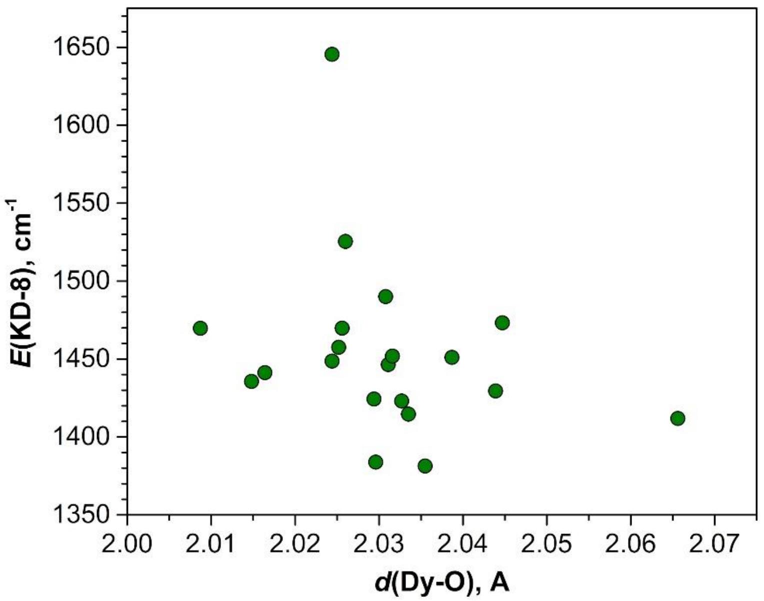

Figure S75. The energy of the whole ligand-field splitting (KD-8) in DFT-optimized $Dy_2O@C_{82}$ conformers plotted versus the Dy–O bond lengths.

Relaxation times and magnetization decay curves of $Dy_2O@C_{82}$ isomers

Table S22. Relaxation times of $Dy_2O@C_{82}$-C_s measured in zero magnetic field

T, K	*τ*, s	st. dev. *τ*, s	β
1.80	839.2	6.3	0.51
1.90	673.3	1.0	0.62
2.00	467.9	0.7	0.64
2.10	365.3	0.7	0.63
2.20	273.9	0.8	0.61
2.35	202.4	1.8	0.70
2.50	158.2	0.6	0.70
2.70	118.4	0.3	0.69
2.80	100.5	1.2	0.67
3.10	70.8	1.1	0.65
3.30	56.4	1.0	0.63
4.00	32.8	1.3	0.74

Table S23. Relaxation times of $Dy_2O@C_{82}$-C_s measured at 1.8 K in different magnetic fields

H, T	*τ*, s	st. dev. *τ*, s	β
0.00	839.2	6.3	0.51
0.10	794.6	1.9	0.63
0.20	756.6	1.2	0.62
0.30	673.2	2.9	0.61
0.40	569.9	2.4	0.58
0.45	447.8	18.1	0.50
0.50	380.8	2.8	0.47
0.55	200.5	15.5	0.45
0.60	135.4	1.4	0.47
0.70	81.0	0.6	0.59
0.80	80.5	0.6	0.72
0.90	78.5	0.9	0.74
1.00	85.9	0.5	0.67
1.10	119.1	0.9	0.62
1.20	221.9	1.4	0.57
1.40	270.7	1.1	0.57
1.60	229.4	1.0	0.59
1.80	196.6	1.1	0.62
2.00	158.1	0.9	0.63

Table S24. Relaxation times of $Dy_2O@C_{82}$-C_s measured at 2.5 K in different magnetic fields

H, **T**	***τ*, s**	**st. dev. *τ*, s**	**β**
0.00	158.2	0.6	0.70
0.10	149.8	1.1	0.72
0.20	138.2	1.4	0.71
0.30	128.3	0.6	0.70
0.40	109.4	1.8	0.64
0.50	97.5	1.7	0.67
0.60	75.4	0.8	0.61
0.70	71.7	0.6	0.64
0.80	73.0	0.7	0.63
0.90	76.4	0.7	0.67
1.00	78.5	0.9	0.62
1.10	98.7	1.0	0.57
1.20	142.6	1.9	0.66
1.40	168.9	1.8	0.69
1.60	148.9	2.2	0.71
1.80	124.2	2.0	0.67
2.00	110.9	1.5	0.71

Table S25. Relaxation times of $Dy_2O@C_{82}$-C_s measured at different temperatures in the field of 0.8 T.

***T*, K**	***τ*, s**	**st. dev. *τ*, s**	**β**
1.80	77.3	1.2	0.68
1.90	75.5	1.9	0.69
2.00	72.5	1.2	0.69
2.10	68.3	0.8	0.67
2.20	63.7	1.4	0.68
2.24	63.4	1.0	0.60
2.50	60.6	1.8	0.63
2.65	62.7	0.9	0.66
2.80	54.9	1.6	0.65
3.00	53.8	1.1	0.69

Table S26. Relaxation times of $Dy_2O@C_{82}$-C_{3v} measured in zero magnetic field

T, K	*τ*, s	st. dev. *τ*, s	β
1.80	12083.5	7.2	0.55
1.90	9532.3	10.9	0.53
2.00	7423.0	8.4	0.51
2.10	6160.8	11.3	0.51
2.20	5004.4	10.9	0.49
2.28	4325.0	9.4	0.49
2.35	3845.8	8.8	0.48
2.42	3444.1	8.5	0.49
2.50	3052.6	8.2	0.49
2.65	2597.1	15.2	0.52
2.80	2099.9	7.5	0.48
3.00	1473.6	3.6	0.50
3.00	1527.8	7.9	0.50
3.10	1350.5	3.0	0.49
3.30	1032.6	2.2	0.53
3.50	800.3	1.6	0.52
3.70	620.3	1.9	0.56
3.70	624.8	1.5	0.54
3.80	563.5	1.7	0.55
4.00	451.8	1.2	0.56
4.25	326.9	1.5	0.59
4.50	269.8	0.7	0.60
5.00	189.6	1.2	0.63
5.35	149.1	1.0	0.65
5.80	107.5	0.8	0.67
6.00	88.5	1.1	0.64
6.20	72.3	1.6	0.62

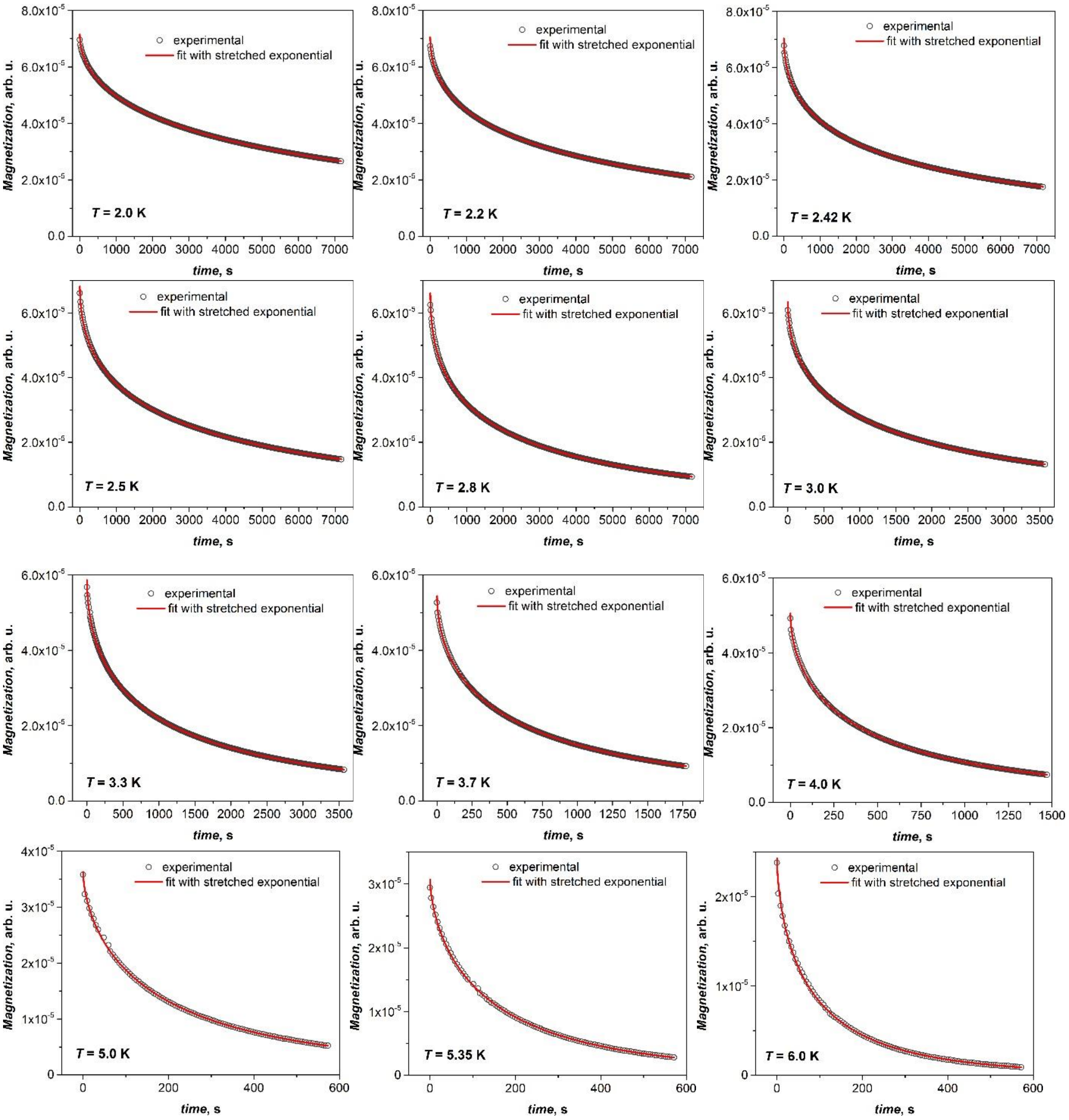

Figure S76. Representative example of determination of relaxation times: Magnetization decay curves of $Dy_2O@C_{82}$-C_{3v} measured in zero magnetic field at selected temperatures and their stretch exponential fits.

Table S27. Relaxation times of $Dy_2O@C_{82}$-C_{3v} measured at 2.5 K in different magnetic fields

H, T	*τ*, s	st. dev. *τ*, s	β
0	3052.6	8.2	0.49
0.025	2935.7	20.8	0.48
0.05	2734.1	17.9	0.49
0.075	2563.8	13.9	0.49
0.1	2344.8	14.0	0.50
0.2	1705.0	8.9	0.53
0.3	1289.1	5.2	0.53
0.4	1019.5	4.8	0.55
0.5	871.0	10.0	0.53
0.6	744.5	5.0	0.51
0.7	582.2	3.4	0.53
0.8	476.2	2.9	0.55
0.9	414.5	2.2	0.56
1.0	377.8	1.9	0.56
1.1	342.3	1.8	0.56
1.2	362.0	2.2	0.54
1.3	420.7	2.9	0.57
1.4	467.3	2.5	0.61
1.5	476.6	3.8	0.60
1.6	470.7	4.2	0.55
1.8	430.0	3.9	0.54
2.0	406.5	15.0	0.51
2.2	381.1	5.6	0.58
2.4	358.6	5.2	0.54
2.6	343.0	6.9	0.57
2.8	312.6	7.8	0.55
3.0	292.5	7.6	0.58

Table S28. Relaxation times of $Dy_2O@C_{82}$-C_{3v} measured at different temperatures in the field of 1.1 T.

T, K	*τ*, s	st. dev. *τ*, s	β
1.8	372.9	4.7	0.57
1.9	356.8	3.2	0.60
2.0	368.0	4.0	0.60
2.1	370.5	4.4	0.60
2.2	346.4	2.3	0.61
2.24	339.4	3.0	0.60
2.5	314.8	2.7	0.62
2.65	319.9	2.4	0.59
2.8	292.5	1.9	0.59
3.0	279.6	2.5	0.61
3.3	251.9	1.9	0.61
3.5	222.0	1.7	0.61
4.0	156.5	1.1	0.63
4.5	114.6	0.7	0.65
5.0	80.3	0.6	0.65

Table S29. Relaxation times of $Dy_2O@C_{82}$-C_{2v} measured in zero magnetic field

T, K	*τ*, s	st. dev. *τ*, s	β
1.80	18443.2	12.4	0.54
1.90	11018.9	11.0	0.56
2.00	7781.9	17.7	0.69
2.10	4104.6	2.4	0.60
2.20	3221.2	3.7	0.63
2.35	1626.5	1.5	0.63
2.50	1094.5	0.8	0.71
2.65	678.0	1.7	0.62
2.80	496.2	1.1	0.69
3.00	303.3	0.8	0.70
3.30	176.3	0.9	0.69
3.50	130.3	0.7	0.68
4.00	77.8	0.6	0.68

Table S30. Relaxation times of $Dy_2O@C_{82}$-C_{2v} measured at 2.5 K in different magnetic fields

H, T	*τ*, s	st. dev. *τ*, s	β
0.0	1094.5	0.8	0.71
0.1	980.4	1.0	0.64
0.2	931.1	1.1	0.63
0.3	860.5	1.5	0.61
0.4	786.3	1.2	0.60
0.5	697.4	1.5	0.57
0.6	651.1	4.3	0.62
0.7	547.8	2.9	0.60
0.8	398.9	3.0	0.53
0.9	242.2	1.7	0.48
1.0	157.6	1.2	0.48
1.2	155.9	1.7	0.55
1.4	165.5	1.8	0.56
1.6	204.4	2.4	0.57
1.8	268.2	2.0	0.58
2.0	323.9	4.0	0.56
2.2	330.7	4.3	0.56
2.4	380.2	5.1	0.55
2.6	330.9	3.5	0.57
2.8	338.7	5.2	0.56
2.8	340.2	6.9	0.51

Table S31. Relaxation times of $Dy_2O@C_{82}$-C_{2v} measured at different temperatures in the field of 1.2 T.

T, K	*τ*, s	st. dev. *τ*, s	β
1.80	136.5	2.0	0.63
1.90	136.5	1.4	0.60
2.00	136.6	1.2	0.60
2.10	142.2	1.7	0.58
2.20	140.7	1.6	0.55
2.24	135.6	1.2	0.60
2.50	148.9	1.2	0.58
2.65	143.7	1.0	0.60
2.80	143.1	2.0	0.60
3.00	138.2	1.1	0.63
3.30	111.9	1.1	0.60
3.50	104.7	1.1	0.60
4.00	97.0	1.1	0.61

DFT-optimized Cartesian coordinates of selected $Dy_2O@C_{82}$ conformers

$Dy_2O@C_{82}$-C_s, conf. 1

Dy	1.581696000	1.206244000	0.370844000
Dy	-1.590640000	0.007056000	-1.278543000
O	0.000000000	0.000000000	0.000000000
C	0.537265000	3.049679000	2.553032000
C	-0.840480000	2.718232000	2.750265000
C	-1.759203000	3.295825000	1.830062000
C	-1.310642000	4.014938000	0.657274000
C	0.071859000	4.242086000	0.421825000
C	0.998507000	3.810935000	1.425633000
C	2.376897000	3.450072000	1.149607000
C	2.809492000	3.349106000	-0.234944000
C	1.861492000	3.747091000	-1.238222000
C	0.538200000	4.242757000	-0.922862000
C	-0.342029000	3.915015000	-2.003099000
C	0.421217000	3.215544000	-3.002134000
C	1.770933000	3.112346000	-2.536282000
C	2.557793000	1.964044000	-2.833671000
C	2.009701000	0.973035000	-3.691955000
C	0.659854000	1.083128000	-4.173219000
C	-0.163341000	2.165155000	-3.776021000
C	-1.557037000	1.884205000	-3.576151000
C	-2.139599000	0.567250000	-3.749410000
C	-1.253413000	-0.555135000	-3.995720000
C	0.109717000	-0.254543000	-4.287983000
C	1.158443000	-1.167107000	-3.930329000
C	0.861980000	-2.388399000	-3.337988000
C	-0.504751000	-2.736486000	-3.053339000
C	-1.568311000	-1.837657000	-3.346937000
C	-2.731659000	-1.901161000	-2.477589000
C	-2.620840000	-2.632035000	-1.224477000
C	-1.497497000	-3.445019000	-0.871443000
C	-0.494351000	-3.568593000	-1.859855000
C	0.876791000	-3.734981000	-1.474635000
C	1.208277000	-3.937761000	-0.153785000
C	0.177236000	-3.974776000	0.860144000
C	-1.159413000	-3.608517000	0.547095000
C	-1.935779000	-2.897219000	1.564323000
C	-1.309491000	-2.613007000	2.807634000
C	0.069620000	-2.925602000	3.060943000
C	0.805877000	-3.599824000	2.096336000
C	2.170159000	-3.199424000	1.809479000
C	2.743126000	-2.146432000	2.515168000

C	1.974896000	-1.442929000	3.522502000
C	0.661417000	-1.817695000	3.786331000
C	-0.353371000	-0.817245000	3.991283000
C	-0.072692000	0.575803000	3.860565000
C	1.321380000	0.950907000	3.625791000
C	2.315555000	-0.054193000	3.459890000
C	3.367330000	0.099853000	2.463874000
C	3.626835000	1.335566000	1.798357000
C	2.768026000	2.452819000	2.147283000
C	1.601237000	2.203230000	2.982387000
C	-1.156895000	1.458378000	3.410415000
C	-2.424095000	0.878435000	3.113124000
C	-3.284528000	1.411689000	2.094168000
C	-2.962416000	2.609108000	1.467620000
C	-3.214496000	2.791740000	0.066190000
C	-3.747304000	1.731816000	-0.724542000
C	-4.067635000	0.455531000	-0.068353000
C	-3.852444000	0.330985000	1.332141000
C	-3.397194000	-0.908133000	1.942458000
C	-3.043874000	-2.027427000	1.154599000
C	-3.374774000	-1.939118000	-0.234366000
C	-3.966270000	-0.763341000	-0.852658000
C	-3.635988000	-0.779945000	-2.269859000
C	-3.328416000	0.478976000	-2.916114000
C	-3.372939000	1.698662000	-2.125939000
C	-2.314170000	2.578072000	-2.581897000
C	-1.708791000	3.580242000	-1.751487000
C	-2.199426000	3.702155000	-0.428434000
C	2.392884000	-3.333402000	0.393373000
C	3.136948000	-2.365293000	-0.344824000
C	3.766518000	-1.290219000	0.420830000
C	3.560969000	-1.194764000	1.826324000
C	4.072814000	1.242599000	0.421876000
C	4.058301000	-0.052557000	-0.241351000
C	3.718928000	0.135934000	-1.613095000
C	3.478921000	1.535862000	-1.825549000
C	3.682772000	2.239652000	-0.573575000
C	3.088931000	-0.881228000	-2.396526000
C	2.786296000	-2.154598000	-1.755377000
C	1.714660000	-2.914024000	-2.308225000
C	2.306304000	-0.429298000	-3.495908000
C	-2.599442000	-0.549456000	3.078661000
C	-1.586213000	-1.377494000	3.506736000

$Dy_2O@C_{82}$-C_{3v}, conf. 1

Dy	-0.176191590	-0.122389810	2.003299050
Dy	1.138267740	-0.500623200	-1.611561230
O	0.000000000	0.000000000	0.000000000
C	0.197235010	-2.482434070	-3.380728520
C	0.390578130	-3.520259260	-2.382806600
C	1.625443300	-3.625426130	-1.685737020
C	2.671625830	-2.719213580	-2.089480640
C	2.546570120	-1.754665640	-3.175253400
C	1.241322190	-1.587486640	-3.810455390
C	0.813680280	-0.225688340	-4.090723120
C	-0.577876130	0.163775930	-3.935540200
C	-1.608026780	-0.763390560	-3.574794150
C	-1.202741020	-2.106817220	-3.320451120
C	-1.842240640	-2.888021720	-2.307848130
C	-0.860504400	-3.762570570	-1.725008300
C	-0.907643320	-4.102084380	-0.336595840
C	0.334325230	-4.297039150	0.336862370
C	1.589739400	-4.058478230	-0.328119240
C	2.518171730	-3.549665440	0.648992560
C	3.489347980	-2.573993310	0.293809690
C	3.555968190	-2.189402990	-1.088370340
C	3.943662940	-0.871965410	-1.494370290
C	3.344341280	-0.582829720	-2.779654860
C	2.876594760	0.773490550	-3.010939420
C	1.613386670	0.926978010	-3.687411950
C	0.702078470	1.987814690	-3.299112580
C	-0.636252080	1.522530280	-3.477730300
C	-1.719256130	2.025810380	-2.696350500
C	-2.805087290	1.132100710	-2.495183550
C	-2.731639560	-0.253742250	-2.878662060
C	-3.372970610	-1.037424860	-1.845588330
C	-2.898224590	-2.333102260	-1.514057100
C	-2.979153710	-2.719126060	-0.144496730
C	-2.009761110	-3.613715280	0.434795430
C	-1.846870680	-3.251584220	1.807011620
C	-0.589700350	-3.379129440	2.456039030
C	0.491254220	-3.945292160	1.720851490
C	1.841335380	-3.483736500	1.914955120
C	2.130578520	-2.440947260	2.840011490
C	3.168048920	-1.522863310	2.520699510
C	3.853315680	-1.594618870	1.265671950
C	4.192954880	-0.253003150	0.870275450
C	4.236289320	0.130010880	-0.500690070

C	3.905110480	1.469870700	-0.814441320
C	3.197204760	1.777240970	-2.044286930
C	2.324616260	2.898165840	-1.770122840
C	1.013142250	2.970794960	-2.314230390
C	-0.075728620	3.553433180	-1.523535020
C	-1.449639450	3.078732360	-1.717458210
C	-2.368442260	3.212526980	-0.635139720
C	-3.373161950	2.205486540	-0.379087010
C	-3.577561470	1.189058560	-1.286667170
C	-3.859112180	-0.141186530	-0.832852000
C	-3.784384810	-0.463304350	0.555931730
C	-3.401381620	-1.793454360	0.867917730
C	-2.700711770	-2.108342430	2.081677860
C	-2.383922750	-1.123245660	3.055601840
C	-1.204195560	-1.370253280	3.889063890
C	-0.289457210	-2.415749020	3.480014550
C	1.061768940	-1.947625200	3.663583670
C	1.016340260	-0.599425390	4.179313430
C	1.967483380	0.376183010	3.654822070
C	3.057803220	-0.128291640	2.897889080
C	3.697073930	0.650250450	1.874278150
C	3.262172240	1.960056890	1.553334290
C	3.478141920	2.386699280	0.205067970
C	2.573465670	3.306214270	-0.418890690
C	1.573931810	3.906724140	0.321572150
C	0.268895050	4.118803520	-0.261574370
C	-0.664515320	4.165940530	0.829397520
C	-1.961531330	3.717505470	0.645066120
C	-2.608549760	2.921492840	1.675679280
C	-3.423151750	1.930062060	1.037174400
C	-3.504352810	0.591114290	1.529885450
C	-2.793975420	0.264241150	2.780878520
C	-2.048325690	1.308869490	3.438785580
C	-1.934035400	2.620532380	2.851388760
C	-0.575380080	3.084801870	3.040030570
C	0.047846450	3.835437540	2.052038740
C	1.424213420	3.598213910	1.723042080
C	2.168465340	2.542792480	2.331962790
C	1.513480720	1.743588400	3.378765180
C	0.155016750	2.060504220	3.742280950
C	-0.804743700	1.046939930	4.156664420
C	-0.387913360	-0.278615780	4.415017890

$Dy_2O@C_{82}$-C_{3v}, conf. 2

Dy	-1.849062610	0.628525320	-0.501620180
Dy	1.300854590	-1.504717440	-0.470087880
O	0.000000000	0.000000000	0.000000000
C	-0.128295050	-2.508791750	-3.504694170
C	0.100536170	-3.530845640	-2.507648920
C	1.364916690	-3.699773830	-1.849048860
C	2.454450510	-2.826656000	-2.239784860
C	2.155481490	-1.740251510	-3.153219240
C	0.885453800	-1.558795720	-3.806345550
C	0.483785910	-0.227828030	-4.136448270
C	-0.892646970	0.159874190	-4.064790380
C	-1.892944540	-0.770487180	-3.682479720
C	-1.509439640	-2.124329580	-3.447974350
C	-2.146251460	-2.905315740	-2.421149580
C	-1.159445230	-3.771622020	-1.842294850
C	-1.203334680	-4.115782950	-0.461611630
C	0.054238230	-4.302719920	0.197426480
C	1.324033100	-4.122799530	-0.473770070
C	2.287375560	-3.632113770	0.510104740
C	3.338366230	-2.697965450	0.150840400
C	3.440554400	-2.333943000	-1.262288000
C	3.689276120	-0.938605370	-1.605944040
C	2.937245900	-0.599895850	-2.777597260
C	2.511564840	0.743387200	-3.062863900
C	1.278513870	0.909680680	-3.750253050
C	0.393597530	1.994500150	-3.428293660
C	-0.954080500	1.538126840	-3.614225030
C	-2.028903800	2.041886690	-2.835710320
C	-3.161059210	1.148897120	-2.619603730
C	-3.007721960	-0.248199350	-2.947698100
C	-3.650754480	-1.030245800	-1.925896950
C	-3.184858520	-2.350869950	-1.615378350
C	-3.272983260	-2.751954180	-0.257791430
C	-2.303197700	-3.633223660	0.315067210
C	-2.127050790	-3.253564920	1.693074630
C	-0.865991730	-3.392611270	2.338068000
C	0.217884710	-3.942592330	1.575156890
C	1.566580470	-3.503896940	1.764988470
C	1.856194250	-2.475186260	2.723986720
C	2.883599090	-1.552482560	2.380771530
C	3.574820020	-1.643811170	1.109009160
C	3.921550610	-0.285897920	0.731881540
C	3.966597500	0.096354970	-0.636728800

C	3.625569530	1.436218900	-0.949717520
C	2.904047890	1.762256600	-2.162815310
C	2.039747740	2.877020550	-1.892913170
C	0.722380670	2.957811120	-2.438884370
C	-0.356751790	3.539407530	-1.639844160
C	-1.739937410	3.075543370	-1.833275330
C	-2.678641780	3.207803030	-0.741290680
C	-3.749651250	2.246430060	-0.494857800
C	-4.011719940	1.224944950	-1.438480620
C	-4.222784580	-0.132586130	-0.948884410
C	-4.102925320	-0.481178610	0.463710250
C	-3.690889190	-1.806973640	0.763420250
C	-2.980585870	-2.131203850	1.967260190
C	-2.624846700	-1.131091060	2.909709700
C	-1.443840780	-1.379949430	3.673180150
C	-0.553075800	-2.462053850	3.359671760
C	0.807175060	-2.004808750	3.552810430
C	0.759155140	-0.629285880	3.973283580
C	1.691194670	0.336947370	3.497981160
C	2.795928510	-0.170317500	2.757843010
C	3.435194400	0.611364770	1.741584970
C	2.989459070	1.924499280	1.423513830
C	3.195582570	2.347731980	0.079647200
C	2.292258920	3.274607860	-0.537753870
C	1.301580040	3.881711100	0.203698430
C	-0.002809990	4.097989150	-0.372489750
C	-0.932007560	4.135005410	0.719034970
C	-2.239311530	3.698493790	0.542338000
C	-2.884944800	2.912729990	1.572262890
C	-3.730718960	1.933492890	0.931643830
C	-3.792203730	0.578538720	1.435149580
C	-3.052471970	0.246432070	2.665715000
C	-2.305308190	1.284327550	3.306576460
C	-2.193013070	2.594170650	2.734482300
C	-0.831384960	3.064605770	2.928448040
C	-0.214986610	3.818716670	1.943104930
C	1.158184910	3.570052300	1.609510110
C	1.900071120	2.511844490	2.209981370
C	1.247113910	1.713108150	3.253614210
C	-0.093387300	2.040153900	3.610070130
C	-1.048632880	1.010025680	3.958347650
C	-0.626517240	-0.290973530	4.125398440

$Dy_2O@C_{82}$-C_{3v}, conf. 3

Dy	-0.047038200	-1.206670060	1.624783360
Dy	0.764852680	-0.212491460	-1.872202440
O	0.000000000	0.000000000	0.000000000
C	0.119890030	-2.563621530	-3.482593720
C	0.321490930	-3.596576520	-2.486468540
C	1.552925330	-3.707387170	-1.783624860
C	2.593335590	-2.805851380	-2.168266500
C	2.407105240	-1.802791920	-3.197365700
C	1.152336660	-1.651048360	-3.895132110
C	0.754298340	-0.296064160	-4.286435950
C	-0.638747820	0.079960970	-4.073712040
C	-1.673875170	-0.849339890	-3.684960380
C	-1.278936310	-2.193749930	-3.426563460
C	-1.918377540	-2.976761290	-2.412294090
C	-0.932861840	-3.849383370	-1.833421970
C	-0.979358880	-4.186327030	-0.446230390
C	0.259021420	-4.376356880	0.236585100
C	1.513605990	-4.146997410	-0.423657630
C	2.442122370	-3.632191590	0.546294760
C	3.429329970	-2.665643120	0.185657460
C	3.520195510	-2.297482840	-1.194679290
C	3.889345780	-0.969778040	-1.587094530
C	3.216885500	-0.652867490	-2.826242890
C	2.800716740	0.693175950	-3.128202270
C	1.568172240	0.867375710	-3.884931930
C	0.649288370	1.908745210	-3.435033180
C	-0.692275740	1.431500840	-3.578308890
C	-1.781615790	1.942372340	-2.794911980
C	-2.867821920	1.051746040	-2.594897940
C	-2.791922960	-0.338024690	-2.979076030
C	-3.433372530	-1.120006120	-1.947455410
C	-2.967118330	-2.417718000	-1.616337500
C	-3.044229610	-2.801591940	-0.243168950
C	-2.067547000	-3.684768170	0.333288090
C	-1.902318260	-3.328672230	1.718923940
C	-0.672499520	-3.528295200	2.436006020
C	0.409226090	-4.024606920	1.629132130
C	1.766508770	-3.559250310	1.811336980
C	2.062465930	-2.546322690	2.777553460
C	3.083621370	-1.610590350	2.419870460
C	3.773020570	-1.684465260	1.160170330
C	4.130915140	-0.346912050	0.770250660
C	4.173169840	0.030570790	-0.603974510

C	3.827900290	1.368412770	-0.922781180
C	3.124906750	1.685311580	-2.150278100
C	2.258795440	2.815055360	-1.872962950
C	0.952726840	2.889868050	-2.423459240
C	-0.136572410	3.468285460	-1.629952410
C	-1.512349110	2.997457920	-1.821050010
C	-2.429192870	3.131180610	-0.736534440
C	-3.429532540	2.123920780	-0.477964710
C	-3.641127280	1.110178210	-1.388580630
C	-3.916211660	-0.219495390	-0.931845920
C	-3.843463920	-0.538930050	0.454852870
C	-3.467077740	-1.874135800	0.766513940
C	-2.763331330	-2.185660280	1.975174960
C	-2.426071880	-1.199398700	2.939993810
C	-1.267354540	-1.486188280	3.776376780
C	-0.371782600	-2.629135320	3.539986130
C	1.010710310	-2.119574540	3.672736000
C	0.946501260	-0.708679590	4.008639360
C	1.885793060	0.281137780	3.515889940
C	2.993969100	-0.215408170	2.789454800
C	3.641024100	0.559390960	1.772527880
C	3.194795210	1.867282260	1.448631100
C	3.404646490	2.287755520	0.099004140
C	2.510377000	3.217791760	-0.521295730
C	1.509326950	3.814361270	0.220323680
C	0.208912880	4.030040410	-0.364105340
C	-0.724653410	4.080106860	0.727679490
C	-2.021343490	3.636976320	0.544464550
C	-2.670331310	2.844859400	1.577032180
C	-3.483086410	1.849696700	0.938826520
C	-3.560838180	0.512572510	1.430562410
C	-2.845873360	0.188065160	2.674704990
C	-2.112727250	1.230963740	3.320345500
C	-1.999596310	2.545194180	2.751312470
C	-0.635084610	3.007463220	2.941145320
C	-0.013259510	3.752969850	1.952754050
C	1.361775480	3.507266500	1.622811930
C	2.103151150	2.451172630	2.229858990
C	1.443761320	1.654738550	3.271307640
C	0.097922280	1.978473690	3.625719660
C	-0.856015840	0.953904110	3.971339850
C	-0.439549990	-0.366193340	4.154156480

$Dy_2O@C_{82}$-C_{3v}, conf. 4

Dy	-0.923321040	1.612609340	0.762828860
Dy	1.163827610	-1.575884810	-0.654743270
O	0.000000000	0.000000000	0.000000000
C	-0.019458280	-2.483714600	-3.472856480
C	0.220322490	-3.513424810	-2.478385910
C	1.496264820	-3.718063590	-1.846040350
C	2.569089200	-2.812519580	-2.230796450
C	2.266869450	-1.716360670	-3.140873030
C	0.990084300	-1.529172000	-3.782287370
C	0.583705730	-0.200189040	-4.110579810
C	-0.799561420	0.181402740	-4.027866040
C	-1.804681860	-0.758762110	-3.659926010
C	-1.401916930	-2.112634940	-3.417303700
C	-2.032571020	-2.897837570	-2.395145790
C	-1.044856750	-3.757843360	-1.814310420
C	-1.091413070	-4.107796590	-0.433246280
C	0.169745040	-4.294365540	0.221877910
C	1.439640130	-4.113778400	-0.456263470
C	2.372273320	-3.579095630	0.531536950
C	3.380773850	-2.616710430	0.177930070
C	3.494097470	-2.272296910	-1.223718850
C	3.757788900	-0.889508720	-1.565345110
C	3.027894070	-0.563990580	-2.749062250
C	2.601092650	0.779690770	-3.032605900
C	1.373799930	0.938265540	-3.728732910
C	0.478839980	2.020530320	-3.403935190
C	-0.860834750	1.552820300	-3.587694870
C	-1.929848060	2.029907780	-2.787329470
C	-3.010849460	1.118772800	-2.569901340
C	-2.930075610	-0.260186500	-2.952475710
C	-3.570827760	-1.055395060	-1.921045550
C	-3.088018660	-2.350266560	-1.594446400
C	-3.164221090	-2.748574980	-0.228855560
C	-2.191568710	-3.636359480	0.344464960
C	-2.017734120	-3.264867400	1.721793650
C	-0.756172880	-3.388581210	2.366257360
C	0.330478830	-3.929628970	1.599928910
C	1.671526890	-3.474483150	1.791576660
C	1.966000870	-2.449287540	2.751116440
C	2.991555730	-1.521543610	2.416903360
C	3.674519590	-1.596499690	1.149521430
C	4.022398840	-0.248391960	0.772667470
C	4.051835490	0.135143060	-0.594821070

C	3.711270710	1.473897590	-0.910768980
C	2.991584800	1.796503500	-2.127190740
C	2.120058170	2.905063550	-1.855418280
C	0.803928270	2.976018850	-2.408514000
C	-0.283258780	3.542900090	-1.603441210
C	-1.651715110	3.071489120	-1.794602000
C	-2.576138530	3.212836130	-0.687733700
C	-3.557458530	2.185157790	-0.435922760
C	-3.761878360	1.164496290	-1.349199680
C	-4.048911650	-0.166795630	-0.902982620
C	-3.966980050	-0.495804440	0.487122320
C	-3.588819710	-1.826277770	0.795439940
C	-2.882667450	-2.145399420	1.998420540
C	-2.524917010	-1.145975440	2.937244650
C	-1.340986400	-1.382253510	3.702962340
C	-0.444347230	-2.458628270	3.390583090
C	0.913941900	-1.989386360	3.583088740
C	0.857775830	-0.617296860	4.004406230
C	1.791204280	0.356698610	3.537128380
C	2.902726210	-0.137900460	2.802364830
C	3.537543650	0.647677890	1.788970260
C	3.080849980	1.954046140	1.466053990
C	3.277150320	2.380629630	0.117976730
C	2.363710280	3.298488040	-0.499130390
C	1.367499960	3.900232630	0.247131320
C	0.059021450	4.114921420	-0.325378220
C	-0.878972980	4.204116950	0.770560060
C	-2.212131720	3.778324320	0.593991790
C	-2.864555220	2.972378160	1.640865060
C	-3.616370340	1.924983410	0.983041880
C	-3.678870190	0.559508570	1.462321200
C	-2.954536970	0.232966930	2.687898930
C	-2.221024770	1.291586520	3.336120480
C	-2.148395470	2.634427480	2.808623110
C	-0.769865550	3.091873020	2.986475850
C	-0.150158470	3.859220140	1.990916960
C	1.224651650	3.590661230	1.647102840
C	1.984812270	2.529169960	2.252276360
C	1.339624490	1.728907280	3.289429530
C	-0.018099720	2.051363090	3.643218190
C	-0.960190660	1.016384690	3.985637010
C	-0.531720350	-0.287246210	4.149650880

$Dy_2O@C_{82}$-C_{3v}, conf. 5

Dy	-0.655578410	-1.692096920	0.909011530
Dy	0.995932610	0.332100260	-1.736978990
O	0.000000000	0.000000000	0.000000000
C	0.119928940	-2.616152490	-3.571076880
C	0.334920390	-3.638264730	-2.577257680
C	1.570243080	-3.739556920	-1.883197660
C	2.617487520	-2.843326130	-2.263014050
C	2.403158870	-1.824154820	-3.252335690
C	1.137737120	-1.665872900	-3.890117920
C	0.753297430	-0.313834530	-4.246966330
C	-0.630727460	0.053391270	-4.088098890
C	-1.657860830	-0.888898640	-3.741774220
C	-1.268002930	-2.239533350	-3.515328340
C	-1.905298580	-3.020649340	-2.495227570
C	-0.918011500	-3.888688450	-1.909199810
C	-0.963853280	-4.242249200	-0.525977310
C	0.281569740	-4.425213730	0.143390180
C	1.529873490	-4.177122480	-0.522760470
C	2.456063190	-3.664569430	0.450637250
C	3.450449440	-2.696628510	0.090538660
C	3.545223520	-2.329338520	-1.292001250
C	3.908553510	-1.001194090	-1.684515780
C	3.216216750	-0.668266870	-2.917146890
C	2.852734050	0.682189010	-3.280422820
C	1.587232260	0.860759440	-4.002727530
C	0.655333680	1.936203660	-3.596626160
C	-0.689066880	1.421949680	-3.664377630
C	-1.782898550	1.914289560	-2.872988440
C	-2.855957020	1.006835670	-2.659907170
C	-2.779833030	-0.382743040	-3.042688110
C	-3.420437680	-1.167837220	-2.017439490
C	-2.938259250	-2.458383330	-1.692463280
C	-2.991427270	-2.840696340	-0.309221860
C	-2.076656140	-3.782991640	0.282824250
C	-1.986109370	-3.519749890	1.717134760
C	-0.677587960	-3.645617740	2.370210160
C	0.425656650	-4.089167590	1.549032920
C	1.784854500	-3.604295020	1.713848720
C	2.091236740	-2.572949550	2.652526690
C	3.122926470	-1.647691450	2.325268530
C	3.806095010	-1.719232620	1.061468830
C	4.157930430	-0.380299780	0.670324910
C	4.188919390	-0.003117240	-0.702492900

C	3.835572420	1.336609290	-1.029355520
C	3.161966490	1.672283570	-2.279642630
C	2.264439540	2.783945570	-1.980390620
C	0.952749740	2.881719220	-2.544010830
C	-0.139533750	3.440397140	-1.721485030
C	-1.518853070	2.969994370	-1.901101850
C	-2.429861110	3.093597910	-0.809297000
C	-3.421840550	2.078291490	-0.545767600
C	-3.629155120	1.062138180	-1.453352630
C	-3.897134460	-0.269448350	-0.993197190
C	-3.821658710	-0.584777120	0.390693180
C	-3.421233350	-1.927576330	0.709464670
C	-2.814180530	-2.316972710	1.956743180
C	-2.427084030	-1.260902950	2.865122340
C	-1.229792460	-1.498823520	3.611765410
C	-0.337668690	-2.617093270	3.320987810
C	1.022477340	-2.110397170	3.471119360
C	0.970844310	-0.737033790	3.889582760
C	1.911645120	0.238666040	3.422445560
C	3.020075450	-0.259565830	2.695876850
C	3.657601190	0.521389600	1.676082640
C	3.205175000	1.823715640	1.352831230
C	3.403797580	2.244531480	-0.003026930
C	2.505380040	3.170768720	-0.616856310
C	1.511214780	3.775412750	0.129280050
C	0.210920030	3.992187520	-0.449472020
C	-0.718380600	4.038153500	0.646578500
C	-2.016303400	3.594932830	0.470622800
C	-2.663294050	2.802595590	1.505542440
C	-3.480098590	1.807047470	0.870944490
C	-3.558859800	0.469830690	1.364117080
C	-2.838631190	0.132537930	2.598286980
C	-2.089343180	1.173168750	3.231854370
C	-1.981000730	2.490887610	2.667198050
C	-0.616247400	2.958588070	2.854434000
C	-0.000655900	3.713192320	1.869330680
C	1.371684910	3.466104760	1.533403410
C	2.115897190	2.409089170	2.136000600
C	1.464434020	1.610536880	3.178321300
C	0.121565070	1.933264300	3.534038250
C	-0.831585960	0.903677870	3.878725230
C	-0.409065090	-0.401442010	4.047090270

$Dy_2O@C_{82}$-C_{2v}, conf. 1

Dy	0.742379210	0.834487450	-1.682037740
Dy	-1.004868830	-1.492131240	0.771548120
O	-0.038172160	0.143925130	0.070103470
C	1.227908000	3.958091160	-0.889435470
C	0.013321240	3.888011140	-1.625383580
C	0.034202290	3.133525470	-2.859000930
C	-1.200194050	3.949462890	-0.884403340
C	-1.191176220	4.028791780	0.550430130
C	0.020263430	3.985451150	1.294586280
C	1.231251580	4.036266490	0.539680450
C	-2.367090870	3.187929560	-1.291280540
C	-2.327283700	2.367379990	-2.443546660
C	-1.123172320	2.374586450	-3.231807190
C	-3.092606980	1.170462570	-2.426133510
C	-2.382035350	2.684462110	2.229376530
C	-2.365665750	3.336563290	1.012133690
C	-3.061044400	2.762806420	-0.111074720
C	-3.668596550	1.473401480	-0.016271260
C	-3.743109540	0.727203520	-1.224177140
C	1.231385630	2.677365320	3.037914600
C	0.024777030	3.287062920	2.585460540
C	-1.177448370	2.675180540	3.046614700
C	-2.677189980	0.009860600	-3.171662740
C	-1.454238070	0.004753180	-3.912498750
C	-0.688070850	1.225197010	-3.971852730
C	-0.721522140	-1.210828910	-3.978969090
C	-3.096512820	-1.148487120	-2.440502120
C	-2.337636930	-3.125557440	-1.258939840
C	-2.326219550	-2.337284860	-2.455898530
C	-1.157352680	-2.379663680	-3.271806590
C	-3.070713490	1.436514070	2.378785190
C	-3.643286980	0.751644060	1.253803960
C	-3.649876450	-0.709131640	1.257397160
C	-3.688309620	-1.442046690	-0.012491130
C	-3.751092050	-0.704550830	-1.224097390
C	-3.082695370	-2.761863790	-0.076136840
C	0.727620640	-1.191504330	-3.977607740
C	1.488866820	0.024041760	-4.004941810
C	0.760351060	1.278305730	-4.103917890
C	2.707247710	0.025852630	-3.228062710
C	1.168098000	-2.371667970	-3.269820740
C	2.390865260	-3.160312280	-1.303734650
C	2.347639550	-2.348208860	-2.471773250

C	3.094038810	-1.132511010	-2.454499490
C	-1.189860030	-3.881142530	-0.851338750
C	0.023375590	-3.833252470	-1.617375470
C	0.011193700	-3.103456010	-2.844320990
C	1.240324690	-3.911958130	-0.889389330
C	3.143370900	1.206135330	-2.497219770
C	2.412876630	2.450610080	-2.556181800
C	1.221416330	2.488694020	-3.403317050
C	2.387554480	3.189554760	-1.328311720
C	3.751092050	0.740006200	-1.260291390
C	3.699649810	1.480412970	-0.037148950
C	3.088655880	2.761693800	-0.134950190
C	2.429271260	2.685603530	2.209501440
C	2.408518570	3.339566160	0.991047060
C	3.112951860	-1.374936720	2.364482780
C	3.676235030	-0.702284760	1.233179690
C	3.685692380	0.757316910	1.229296520
C	3.115680320	1.437848530	2.354061540
C	3.741315120	-0.682956980	-1.251959090
C	3.087867510	-2.718423770	-0.117083100
C	3.685609230	-1.429595350	-0.033361580
C	1.239201660	-1.381202540	3.797228830
C	2.427263970	-0.651651920	3.401327560
C	2.426160320	0.724159740	3.389718090
C	-1.173707830	-1.392616100	3.785788780
C	0.037135790	-0.698530860	4.103917890
C	2.406080750	-3.285044630	1.010322180
C	-1.208165620	-2.656108300	3.087511730
C	0.024821350	-3.246273740	2.615244000
C	1.235922180	-2.613182100	3.053003420
C	2.424744370	-2.624497520	2.230345450
C	-2.456157690	-2.722167300	2.313321300
C	-2.456942460	-3.418911060	1.065766400
C	-1.228291130	-4.036266490	0.589422110
C	0.023266680	-3.957981010	1.325627610
C	1.226906330	-3.973469710	0.560786990
C	-2.360953240	0.717329390	3.391567560
C	-2.354235220	-0.665005050	3.397404510
C	-3.076579740	-1.414745150	2.393469470
C	1.234031160	1.459770080	3.780552950
C	0.032019560	0.771150550	4.102516950
C	-1.171979040	1.455382220	3.786703840

$Dy_2O@C_{82}$-C_{2v}, conf. 2

Dy	0.498654710	1.413583510	-1.430743790
Dy	-0.979464550	-1.558727790	0.577585620
O	0.087005210	0.078847990	0.046007940
C	1.218755220	3.987667830	-0.958955920
C	0.001254500	3.957848140	-1.719195890
C	0.014929140	3.256104250	-2.995105080
C	-1.207703150	3.970056560	-0.946448880
C	-1.205548770	4.047043640	0.493509960
C	0.003851560	3.998066010	1.242455870
C	1.210599720	4.038913490	0.483121800
C	-2.380500500	3.213790070	-1.350557910
C	-2.341446710	2.403178880	-2.511836480
C	-1.140341590	2.442035060	-3.314782460
C	-3.096569230	1.197160680	-2.491287440
C	-2.397005880	2.699290500	2.168880420
C	-2.382433870	3.360185990	0.952528520
C	-3.076876100	2.789245110	-0.169907610
C	-3.681135990	1.496557960	-0.079472200
C	-3.747058290	0.751103280	-1.286767190
C	1.215449050	2.688316510	2.984677070
C	0.008177830	3.300286100	2.533571520
C	-1.194173890	2.687447830	2.990600310
C	-2.683988850	0.034172930	-3.233827930
C	-1.455366850	0.030003590	-3.961223080
C	-0.698755950	1.253830350	-3.987793520
C	-0.723475760	-1.184771420	-4.040548440
C	-3.106734580	-1.123839790	-2.506427810
C	-2.351874280	-3.109819840	-1.325266570
C	-2.332777540	-2.313339690	-2.520440680
C	-1.163855580	-2.353570640	-3.330688190
C	-3.084320520	1.453416150	2.314115150
C	-3.657642200	0.772006910	1.188323420
C	-3.658790560	-0.687107150	1.185595820
C	-3.699975650	-1.423635120	-0.079585040
C	-3.758241150	-0.680780980	-1.289479720
C	-3.105654120	-2.751701760	-0.145865090
C	0.720524750	-1.180777510	-4.042054610
C	1.462858990	0.033269870	-3.986473990
C	0.740123060	1.285236840	-4.049266220
C	2.680354540	0.032643160	-3.241935990
C	1.163511090	-2.350485780	-3.328706740
C	2.378744430	-3.136992900	-1.359461360
C	2.343763480	-2.325349070	-2.528705140

C	3.108295330	-1.122310700	-2.508919130
C	-1.206858150	-3.874518080	-0.916021110
C	0.011415650	-3.814790490	-1.679976040
C	0.003548340	-3.080586550	-2.900829260
C	1.228887780	-3.891818570	-0.949005680
C	3.087170160	1.212622570	-2.511789130
C	2.400464660	2.472484900	-2.608367230
C	1.212173010	2.532785510	-3.461207660
C	2.394258080	3.234933670	-1.389006010
C	3.746909320	0.752794270	-1.300975880
C	3.686666150	1.493539540	-0.087224000
C	3.075001300	2.781413770	-0.188299180
C	2.412625380	2.698471050	2.159942660
C	2.388836710	3.348791690	0.935611980
C	3.101583720	-1.360282690	2.310663860
C	3.667452950	-0.686738480	1.180842590
C	3.673377640	0.772695260	1.181041150
C	3.099686650	1.452254230	2.303789850
C	3.758241150	-0.669778270	-1.305049280
C	3.075770820	-2.698366780	-0.173304950
C	3.686092360	-1.413152100	-0.087574160
C	1.219299610	-1.369012140	3.735495780
C	2.410978260	-0.638532670	3.343969350
C	2.409482850	0.736441120	3.339718230
C	-1.190451380	-1.378375940	3.726661630
C	0.018463420	-0.686268230	4.047555850
C	2.389942940	-3.264041450	0.952210250
C	-1.215392410	-2.627358620	3.010561600
C	0.009934540	-3.228864040	2.547304560
C	1.219932910	-2.598043940	2.992498820
C	2.412000050	-2.608345950	2.172781240
C	-2.455302240	-2.687787460	2.223918020
C	-2.479048980	-3.413001220	0.996291990
C	-1.252068170	-4.047043640	0.525254360
C	0.004009850	-3.952230580	1.263427580
C	1.208991540	-3.953381300	0.499936590
C	-2.377090690	0.731428060	3.331804530
C	-2.369956900	-0.647651900	3.333563930
C	-3.076892200	-1.388092120	2.313731770
C	1.216511800	1.470320580	3.729029130
C	0.013772430	0.783544660	4.049266220
C	-1.190464610	1.469027840	3.733613610

$Dy_2O@C_{82}$-C_{2v}, conf. 3

Dy	-0.429397840	1.630957450	-1.265483910
Dy	0.375620180	-1.888132360	-0.106301960
O	0.111895200	0.118836080	-0.022191710
C	1.200336900	4.014831240	-0.943962860
C	-0.009129060	4.047612540	-1.725632500
C	-0.017593360	3.316092830	-2.987352240
C	-1.232409710	4.066300210	-0.957463260
C	-1.210205130	4.076932050	0.490944850
C	0.000332900	4.027874110	1.252534600
C	1.205771610	4.080236880	0.501397600
C	-2.405900990	3.292168980	-1.378514430
C	-2.397042680	2.503673010	-2.580626760
C	-1.207018880	2.560193640	-3.418006350
C	-3.092549340	1.249923070	-2.501677440
C	-2.401690810	2.717738670	2.168192280
C	-2.379619700	3.376134560	0.946323760
C	-3.067497980	2.814062980	-0.178483090
C	-3.680220190	1.522992660	-0.080067510
C	-3.750089490	0.787002530	-1.295582370
C	1.204444700	2.712007130	2.993161120
C	-0.000671710	3.324615590	2.541111600
C	-1.204023940	2.709658750	2.995372770
C	-2.689023830	0.076186210	-3.242429860
C	-1.469121320	0.079044380	-3.976698840
C	-0.739627580	1.322144430	-4.009654320
C	-0.723229040	-1.134345960	-4.047693620
C	-3.116737820	-1.083793570	-2.516194720
C	-2.349710980	-3.087457740	-1.364450240
C	-2.342008760	-2.278017640	-2.538405980
C	-1.160822260	-2.300189480	-3.333535420
C	-3.089277120	1.475064560	2.311058180
C	-3.664908580	0.798891870	1.184999730
C	-3.657388730	-0.658800820	1.180059770
C	-3.673724030	-1.377878910	-0.090172240
C	-3.760341870	-0.637054650	-1.304237950
C	-3.048239980	-2.662040000	-0.179344810
C	0.720033850	-1.134281070	-4.052508450
C	1.453473840	0.083831400	-3.962720830
C	0.697077290	1.304556410	-3.976278010
C	2.679053200	0.084212210	-3.239546510
C	1.164984540	-2.300265480	-3.350142250
C	2.353671960	-3.112940280	-1.369877120
C	2.338947430	-2.276315810	-2.542817380

C	3.105399610	-1.079861860	-2.512859110
C	-1.205947820	-3.876900210	-0.965976890
C	0.014983560	-3.857417630	-1.724807330
C	0.004063050	-3.037248760	-2.903945050
C	1.257438870	-3.991881340	-0.982726850
C	3.098390670	1.246217680	-2.501855690
C	2.340723730	2.450544400	-2.515058610
C	1.137634930	2.496040280	-3.311327630
C	2.372862980	3.254532720	-1.348140370
C	3.756045170	0.797465110	-1.299983640
C	3.684884950	1.535435440	-0.085855870
C	3.075293210	2.827385480	-0.170618980
C	2.403150150	2.726425710	2.167752050
C	2.386701140	3.393754900	0.953744910
C	3.085812060	-1.337368120	2.295566690
C	3.663288510	-0.648650710	1.175058880
C	3.668095310	0.808084970	1.179168790
C	3.092228430	1.481801790	2.306446190
C	3.760341870	-0.630726110	-1.304314350
C	3.028689070	-2.654724000	-0.181890620
C	3.669378800	-1.363610050	-0.094314680
C	1.208628870	-1.350804220	3.732283800
C	2.393576710	-0.617019040	3.334272330
C	2.396214470	0.757679220	3.333119230
C	-1.197107330	-1.350987130	3.727891480
C	0.003770930	-0.665099330	4.047405880
C	2.381917460	-3.272897250	0.940377190
C	-1.199151280	-2.572836980	2.977669940
C	0.010225180	-3.200038210	2.523058810
C	1.214303890	-2.574956700	2.988772090
C	2.408216120	-2.588051220	2.166297270
C	-2.399023200	-2.584476300	2.166234550
C	-2.371414830	-3.241819890	0.941855170
C	-1.198991050	-3.956776490	0.492064990
C	0.017105410	-3.977317840	1.257891800
C	1.258304530	-4.080236880	0.495729110
C	-2.393167140	0.754655640	3.341779360
C	-2.387841530	-0.619186630	3.339288010
C	-3.086179770	-1.339925370	2.305496730
C	1.204596280	1.492197780	3.733098080
C	0.003297280	0.805550300	4.052508450
C	-1.201102230	1.491368040	3.735273070

Dy$_2$O@C$_{82}$-*C*$_{2v}$, conf. 4

Dy	0.807777400	1.712226670	-0.652802260
Dy	-0.807328500	-1.712455550	-0.652844220
O	0.000057610	-0.000043150	0.084074670
C	1.276375320	4.074855960	-0.991553230
C	0.019333400	3.930886610	-1.711060970
C	0.004154180	3.130386600	-2.904299070
C	-1.200344210	3.925636610	-0.953366640
C	-1.209128070	3.999747160	0.493375550
C	0.001086390	3.951576030	1.232667930
C	1.226654990	4.033138310	0.465069950
C	-2.361013470	3.165281500	-1.362846220
C	-2.343152910	2.360122410	-2.532794040
C	-1.157519190	2.390019900	-3.329955110
C	-3.106928420	1.158201700	-2.511381820
C	-2.402081890	2.649967130	2.160050610
C	-2.390202760	3.317139800	0.945380280
C	-3.067783290	2.743534270	-0.180930630
C	-3.683307460	1.457420980	-0.092376740
C	-3.760927130	0.716388160	-1.307747550
C	1.210914480	2.640574270	2.982719820
C	0.004424990	3.248302830	2.523111030
C	-1.202041220	2.638003020	2.980420410
C	-2.674656560	-0.002511500	-3.239467180
C	-1.457515090	0.005461970	-3.985405490
C	-0.715769470	1.225798780	-4.042564080
C	-0.724754070	-1.217685410	-4.036723510
C	-3.094485830	-1.169296850	-2.508497880
C	-2.467370770	-3.303915870	-1.417574330
C	-2.387805390	-2.415255930	-2.563896850
C	-1.176478850	-2.393799540	-3.335887370
C	-3.088188970	1.405459770	2.298749330
C	-3.660192930	0.729667070	1.171176490
C	-3.659682740	-0.731015920	1.171372530
C	-3.681985300	-1.457088170	-0.098006020
C	-3.758099360	-0.709151390	-1.303423880
C	-3.099281650	-2.782537490	-0.204799820
C	0.714346650	-1.224623890	-4.043494100
C	1.456109690	-0.004317680	-3.986187540
C	0.723319620	1.218804920	-4.036611520
C	2.673687610	0.003369190	-3.240920870
C	1.156441840	-2.389059710	-3.331592650
C	2.360824480	-3.164998270	-1.365237720
C	2.342418300	-2.359489700	-2.534878920

C	3.106309900	-1.157592710	-2.513489330
C	-1.276251360	-4.074855960	-0.992514800
C	-0.019576240	-3.930635780	-1.712611290
C	-0.005021160	-3.129646470	-2.905573990
C	1.200419060	-3.925595900	-0.955482900
C	3.093857560	1.169922640	-2.509801690
C	2.387124100	2.415940230	-2.564374610
C	1.175391660	2.394719550	-3.335623340
C	2.467291660	3.304171400	-1.417685090
C	3.758134640	0.709397730	-1.305235450
C	3.682726830	1.456864990	-0.099427210
C	3.099905870	2.782325650	-0.205414410
C	2.406816370	2.652119740	2.157586290
C	2.386058380	3.312759090	0.924272160
C	3.089842390	-1.406516880	2.296573620
C	3.661431050	-0.730397430	1.169032080
C	3.661065420	0.730304250	1.169686740
C	3.090358160	1.409061410	2.297819600
C	3.760927130	-0.716165530	-1.310056630
C	3.068210770	-2.743649350	-0.183514580
C	3.683877290	-1.457640510	-0.094884220
C	1.202415910	-1.421013580	3.725325660
C	2.391740570	-0.685170190	3.327674970
C	2.396609500	0.687877100	3.335158400
C	-1.204927910	-1.423353000	3.727337670
C	0.000772080	-0.736137730	4.043298780
C	2.391219000	-3.317705750	0.942943840
C	-1.208881940	-2.642063870	2.982559270
C	-0.002649000	-3.249552400	2.521960970
C	1.204021550	-2.639292230	2.978779160
C	2.403676050	-2.650934740	2.157798840
C	-2.405132390	-2.653305950	2.157952560
C	-2.384942680	-3.313457910	0.924325440
C	-1.225802190	-4.033781840	0.464090750
C	0.000136190	-3.952395470	1.231173980
C	1.209920850	-4.000210630	0.491251710
C	-2.389505980	0.683723410	3.329148430
C	-2.394247860	-0.689381680	3.336225080
C	-3.088425080	-1.410217430	2.298934520
C	1.207423940	1.421610710	3.726997250
C	0.001807790	0.734420490	4.043494100
C	-1.200033890	1.419463430	3.726489980

$Dy_2O@C_{82}$-C_{2v}, conf. 5

Dy	-0.102853000	-1.269793000	-1.547418000
Dy	0.552840000	0.584072000	1.875032000
O	0.000000000	0.000000000	0.000000000
C	-3.846195000	-0.166513000	1.445669000
C	-3.183782000	-0.164203000	2.716819000
C	-2.487519000	-1.328722000	3.191020000
C	-2.408028000	-2.506336000	2.384025000
C	-3.145667000	-2.543444000	1.166482000
C	-3.868835000	-1.379403000	0.702755000
C	-3.853356000	-1.375168000	-0.737338000
C	-3.131494000	-2.537212000	-1.146738000
C	-2.644453000	-3.236219000	0.017378000
C	-1.355149000	-3.850786000	-0.002760000
C	-0.559222000	-3.857422000	-1.247172000
C	-1.179824000	-3.326186000	-2.473096000
C	-2.400518000	-2.572136000	-2.339305000
C	-2.334773000	-1.378493000	-3.151568000
C	-2.980863000	-0.163221000	-2.724296000
C	-3.764066000	-0.161318000	-1.484907000
C	-1.093387000	-1.412051000	-3.888506000
C	-0.386043000	-0.169012000	-4.127033000
C	1.080946000	-0.160195000	-4.022851000
C	1.733133000	-1.385823000	-3.660980000
C	0.996003000	-2.612512000	-3.381857000
C	-0.403509000	-2.661252000	-3.523136000
C	0.891396000	-3.825044000	-1.145126000
C	1.644221000	-3.264859000	-2.252249000
C	2.872572000	-2.561807000	-2.009782000
C	2.904068000	-1.371438000	-2.819053000
C	3.495962000	-0.164225000	-2.331070000
C	4.124762000	-0.163991000	-1.004070000
C	4.125611000	-1.375892000	-0.255266000
C	3.461525000	-2.543240000	-0.748853000
C	2.828179000	-3.233764000	0.342099000
C	3.187404000	-2.538366000	1.547999000
C	3.966536000	-1.378727000	1.179677000
C	3.855410000	-0.161476000	1.904912000
C	3.017182000	-0.140059000	3.078551000
C	2.251892000	-1.294379000	3.434613000
C	2.297040000	-2.498741000	2.651500000
C	1.108991000	-3.269121000	2.571382000
C	-0.091653000	-2.847077000	3.235604000
C	-0.143019000	-1.623652000	3.976768000

C	1.068563000	-0.856660000	4.121200000
C	-1.360041000	-0.888706000	3.959764000
C	-1.205346000	-3.263014000	2.428084000
C	-0.688204000	-3.922838000	1.252193000
C	0.735934000	-3.925577000	1.340157000
C	1.546780000	-3.839975000	0.178665000
C	-2.483021000	0.994509000	3.177492000
C	-2.412486000	2.168981000	2.373398000
C	-3.148450000	2.209618000	1.161000000
C	-3.866980000	1.050026000	0.699952000
C	-3.860591000	1.047468000	-0.742641000
C	-3.145569000	2.219382000	-1.157499000
C	-2.639114000	2.902941000	0.001585000
C	-1.349489000	3.494110000	-0.013492000
C	-0.549338000	3.481849000	-1.234417000
C	-1.156391000	2.916300000	-2.401378000
C	-2.416212000	2.237077000	-2.337215000
C	-2.353516000	1.042485000	-3.149067000
C	-1.082550000	1.034492000	-3.826953000
C	1.748719000	1.041452000	-3.657553000
C	0.996997000	2.228593000	-3.308967000
C	-0.376601000	2.225848000	-3.391450000
C	0.907285000	3.485603000	-1.145794000
C	1.651152000	2.917136000	-2.227989000
C	2.892004000	2.236501000	-2.012976000
C	2.924498000	1.041626000	-2.828361000
C	4.139406000	1.050349000	-0.256261000
C	3.476656000	2.221853000	-0.752707000
C	2.834375000	2.905061000	0.334340000
C	3.188602000	2.206269000	1.555033000
C	3.973273000	1.051967000	1.173237000
C	2.334189000	1.039478000	3.567859000
C	2.352478000	2.215992000	2.716928000
C	1.129935000	2.966737000	2.609872000
C	-0.087550000	2.555221000	3.299870000
C	-0.136577000	1.342331000	4.104556000
C	1.109080000	0.597402000	4.254638000
C	-1.340303000	0.564945000	3.961516000
C	-1.193615000	2.914922000	2.431840000
C	-0.673425000	3.543596000	1.248963000
C	0.744318000	3.557974000	1.340330000
C	1.558237000	3.503381000	0.163598000

$Dy_2O@C_{82}$-C_{2v}, conf. 6

Dy	-0.212225830	1.958839610	-0.355880900
Dy	-0.218625210	-1.962506010	-0.353613030
O	-0.003891990	-0.001879300	0.124379500
C	1.224752840	3.992502080	-0.976382680
C	-0.001433990	3.992077290	-1.747531140
C	0.002980070	3.131034350	-2.901989480
C	-1.245042450	4.077229900	-0.989967920
C	-1.226402540	4.100388610	0.484102200
C	0.001263030	4.005146520	1.246942010
C	1.214912830	4.032618670	0.485334390
C	-2.347862280	3.198581490	-1.374179110
C	-2.336973540	2.362278490	-2.544308980
C	-1.160153130	2.383473520	-3.344999700
C	-3.103370420	1.163359440	-2.515897410
C	-2.395521030	2.657128500	2.166438520
C	-2.356052470	3.317856290	0.935858580
C	-3.015357410	2.725931030	-0.186721710
C	-3.659746900	1.443640080	-0.096107220
C	-3.760237620	0.715785910	-1.310712890
C	1.210538980	2.640773190	2.983805480
C	0.002535170	3.260007300	2.527424690
C	-1.201323030	2.646361300	2.994783260
C	-2.679694910	-0.000428080	-3.246856760
C	-1.461425960	-0.002555960	-3.988849000
C	-0.719190730	1.218438500	-4.050424250
C	-0.722812190	-1.225737990	-4.048974800
C	-3.106743800	-1.162090200	-2.514292210
C	-2.357018210	-3.198270450	-1.369988600
C	-2.343783340	-2.363217530	-2.541046470
C	-1.167073380	-2.388531210	-3.341811190
C	-3.078819860	1.413922310	2.299092390
C	-3.653984990	0.731046560	1.174165060
C	-3.655967910	-0.724013230	1.175124740
C	-3.663769460	-1.438007240	-0.094242850
C	-3.762218240	-0.711184210	-1.309726290
C	-3.023077960	-2.722265420	-0.183154670
C	0.719373160	-1.228607810	-4.043189220
C	1.462767070	-0.006694340	-3.982649050
C	0.723026690	1.217331680	-4.044627010
C	2.684221990	-0.008029440	-3.246996740
C	1.157515460	-2.391962170	-3.334033070
C	2.345967670	-3.177657690	-1.362079790
C	2.339009630	-2.364386610	-2.535776920

C	3.108479250	-1.169804120	-2.514172670
C	-1.256297300	-4.079226700	-0.984696130
C	-0.012611440	-3.998247950	-1.742365020
C	-0.005989880	-3.138662130	-2.897943430
C	1.213489090	-4.001101410	-0.971220580
C	3.111661040	1.153523840	-2.515629100
C	2.345860690	2.350450460	-2.538749260
C	1.164527460	2.380515480	-3.337096420
C	2.354847960	3.165458490	-1.366164000
C	3.762218240	0.705649660	-1.308324340
C	3.676365560	1.441326420	-0.096475930
C	3.050190180	2.726985560	-0.184161860
C	2.406753160	2.650816930	2.164259720
C	2.378730860	3.304088270	0.938725270
C	3.086159860	-1.411472100	2.301635160
C	3.657774870	-0.734854840	1.175075560
C	3.659658400	0.722449910	1.174119050
C	3.090180810	1.402160310	2.299980790
C	3.760311620	-0.721947970	-1.307504780
C	3.042751230	-2.739567730	-0.180737940
C	3.672557650	-1.455541860	-0.094597850
C	1.202880390	-1.422589080	3.731099340
C	2.395137400	-0.690070430	3.336846660
C	2.397070450	0.683938480	3.336041340
C	-1.202974310	-1.422251070	3.739340410
C	0.002845240	-0.735581670	4.050424250
C	2.369704170	-3.313265490	0.942806140
C	-1.208417410	-2.643729520	2.998237280
C	-0.006152260	-3.260988050	2.531650700
C	1.203467970	-2.644303090	2.987116180
C	2.399585500	-2.658523040	2.167498750
C	-2.402677320	-2.652185110	2.169834790
C	-2.365212200	-3.314506910	0.940123700
C	-1.237580730	-4.100388610	0.489395050
C	-0.009616440	-4.007587620	1.252125850
C	1.203803420	-4.039122180	0.490458470
C	-2.385973900	0.690055980	3.335948600
C	-2.387881630	-0.683955480	3.336942490
C	-3.082783680	-1.407131170	2.301014750
C	1.206746970	1.419938950	3.729279570
C	0.004881620	0.736429830	4.049432690
C	-1.199084240	1.425742590	3.737248540

$Dy_2O@C_{82}$-C_{2v}, conf. 7

Dy	0.287568250	-0.340429760	-1.983391880
Dy	-1.007931720	0.167003630	1.665242930
O	0.137126980	-0.186784320	0.051829090
C	1.229298380	3.928809940	-0.885200310
C	0.010100800	3.856239520	-1.620866250
C	0.009042200	3.106065460	-2.844881210
C	-1.201566130	3.927456660	-0.880833230
C	-1.193554830	4.000619400	0.559681420
C	0.023532240	3.952847350	1.305930160
C	1.230306370	4.005737160	0.554977880
C	-2.367200860	3.180834770	-1.296797330
C	-2.334263050	2.366843490	-2.463924960
C	-1.153585220	2.384250310	-3.271018330
C	-3.089406370	1.164236380	-2.437002100
C	-2.370225080	2.637767320	2.242248960
C	-2.352427600	3.305107930	1.015121270
C	-3.053098120	2.745165890	-0.114448530
C	-3.662577070	1.460245450	-0.019042020
C	-3.746905360	0.718570040	-1.232204510
C	1.237362360	2.646424510	3.047158860
C	0.028643550	3.245109920	2.589585350
C	-1.181001960	2.623089730	3.058686670
C	-2.668766890	0.003069180	-3.176978600
C	-1.465640580	-0.000144620	-3.966818420
C	-0.717562220	1.220537330	-4.003120880
C	-0.718668080	-1.235491000	-4.085526620
C	-3.101873200	-1.155094500	-2.440026630
C	-2.367795290	-3.167076500	-1.300135930
C	-2.339819510	-2.350618410	-2.461774900
C	-1.146758950	-2.353599280	-3.271760480
C	-3.106468240	1.420138660	2.441884660
C	-3.649469160	0.734330660	1.264645060
C	-3.636215260	-0.715330920	1.255546750
C	-3.662442260	-1.448069240	-0.023410990
C	-3.753905820	-0.706857970	-1.233334040
C	-3.055895040	-2.733632520	-0.114585870
C	0.755548020	-1.257055490	-4.154705790
C	1.508110040	-0.003874190	-4.068400260
C	0.740391030	1.220766610	-4.033764610
C	2.672829220	0.001032520	-3.208716140
C	1.176301430	-2.384391690	-3.321383970
C	2.383754280	-3.177254130	-1.310540950
C	2.352756090	-2.368341000	-2.478601270

C	3.099258790	-1.158825880	-2.449270750
C	-1.204758080	-3.918971490	-0.883618690
C	0.008380090	-3.844370750	-1.615434090
C	0.012164080	-3.067978760	-2.829516490
C	1.226768780	-3.927998490	-0.886476390
C	3.095053410	1.161457940	-2.450844260
C	2.348145350	2.372642400	-2.470325910
C	1.167741930	2.386547190	-3.280598190
C	2.388336310	3.184104300	-1.306074650
C	3.750275120	0.713325600	-1.251688910
C	3.685251720	1.458487460	-0.032631230
C	3.086455110	2.744672710	-0.121234140
C	2.427478410	2.661679630	2.230415240
C	2.404226990	3.316458780	1.007042120
C	3.107171330	-1.407519980	2.365033880
C	3.668949420	-0.729942110	1.237351310
C	3.668582230	0.730378810	1.236957710
C	3.106949820	1.406101950	2.366522770
C	3.753905820	-0.710025050	-1.248818220
C	3.082534430	-2.740362510	-0.123366450
C	3.684784910	-1.455941160	-0.032175110
C	1.234241940	-1.417519260	3.800365340
C	2.422416960	-0.689851960	3.404822590
C	2.420414390	0.687184610	3.404365730
C	-1.197420710	-1.410412600	3.831295030
C	0.034220350	-0.731547120	4.140523960
C	2.400692800	-3.313495990	1.003453560
C	-1.178563160	-2.620267920	3.043644500
C	0.025569790	-3.243946720	2.586925380
C	1.233561900	-2.644107970	3.045429360
C	2.424654870	-2.660707010	2.227555480
C	-2.369657030	-2.635041450	2.233972200
C	-2.359929110	-3.301252390	1.011658030
C	-1.198450210	-3.997006150	0.556414650
C	0.018663060	-3.949452470	1.300468460
C	1.227146880	-4.005737160	0.551565830
C	-2.459227830	0.717707670	3.549968680
C	-2.429044530	-0.690331690	3.509922900
C	-3.073868670	-1.396894340	2.410585020
C	1.230532550	1.416142120	3.799587680
C	0.028232270	0.740913200	4.154705790
C	-1.215898230	1.433311640	3.878934360

$Dy_2O@C_{82}$-C_{2v}, conf. 8

Dy	1.801059410	0.000159320	-0.623001470
Dy	-1.801372680	0.000783910	-0.624828860
O	-0.000611740	-0.000253750	0.300106300
C	1.220721410	3.926309190	-0.947122230
C	0.001553170	3.855389560	-1.684007400
C	0.001609470	3.107803420	-2.895657740
C	-1.218017850	3.927205150	-0.947879750
C	-1.213472170	3.992558700	0.486866830
C	0.000695490	3.945103610	1.234599220
C	1.215317050	3.991744040	0.487602350
C	-2.374365130	3.171773760	-1.364042870
C	-2.349951120	2.362889410	-2.529163490
C	-1.164518510	2.383606440	-3.325543750
C	-3.161945900	1.165899560	-2.507138090
C	-2.405026100	2.646376820	2.157355870
C	-2.387328660	3.300992820	0.939363520
C	-3.083292190	2.726239180	-0.185130790
C	-3.794127240	1.475762930	-0.105294590
C	-3.961308300	0.736204030	-1.357288480
C	1.206532320	2.637783120	2.981463610
C	0.000007380	3.243726540	2.521702570
C	-1.207252110	2.638638570	2.980700390
C	-2.689899520	0.001747970	-3.207746570
C	-1.455786060	0.001513120	-3.968525020
C	-0.719832560	1.215671150	-4.037724190
C	-0.720686110	-1.213086160	-4.038344350
C	-3.162854670	-1.162393440	-2.507705420
C	-2.376773230	-3.169237180	-1.365563830
C	-2.351657860	-2.359872360	-2.530316260
C	-1.166263620	-2.381012660	-3.326694240
C	-3.102579820	1.407628140	2.295992620
C	-3.715274670	0.739145920	1.172536910
C	-3.715826640	-0.736891280	1.172192540
C	-3.795290520	-1.472859100	-0.105979160
C	-3.961884210	-0.732609640	-1.357633020
C	-3.085379490	-2.723782400	-0.186433190
C	0.721416700	-1.213579800	-4.037906810
C	1.457302670	0.000517750	-3.967660810
C	0.722282430	1.215174620	-4.037359460
C	2.690887570	-0.000050410	-3.206024810
C	1.165742450	-2.381811180	-3.325970500
C	2.374391510	-3.170832090	-1.364038370
C	2.350633100	-2.361451190	-2.528783970

C	3.162481730	-1.164462710	-2.505623900
C	-1.220943000	-3.925713550	-0.949735210
C	-0.001356460	-3.854394780	-1.685858380
C	-0.000662590	-3.106212770	-2.897109280
C	1.217781730	-3.926512320	-0.948997810
C	3.163479980	1.163746610	-2.505215290
C	2.352341630	2.361227480	-2.527755470
C	1.167434290	2.382756310	-3.324877250
C	2.376756240	3.170017830	-1.362608380
C	3.961884210	0.733541140	-1.354879030
C	3.794736340	1.473254600	-0.102928560
C	3.084632390	2.724106650	-0.183165180
C	2.404804580	2.644602140	2.158872330
C	2.388376660	3.299304310	0.940892080
C	3.100335270	-1.408491440	2.297193600
C	3.713868880	-0.739461940	1.174444950
C	3.714450420	0.736608170	1.174798010
C	3.101338710	1.405354120	2.297912760
C	3.961201080	-0.735287720	-1.355158370
C	3.082640450	-2.725933550	-0.184468220
C	3.793426640	-1.475501590	-0.103631450
C	1.202278080	-1.418126450	3.717401960
C	2.393493710	-0.686766320	3.325317770
C	2.393922610	0.683552740	3.325666830
C	-1.206348190	-1.417160480	3.716610400
C	-0.001873900	-0.734076800	4.037975890
C	2.385985090	-3.301234540	0.939307180
C	-1.209109850	-2.638855540	2.979405590
C	-0.002326150	-3.244670550	2.520193190
C	1.204618360	-2.639812570	2.980194160
C	2.402923580	-2.647195820	2.157633490
C	-2.406905560	-2.645369870	2.156111890
C	-2.389725980	-3.299450150	0.937819220
C	-1.216403500	-3.991773070	0.485011700
C	-0.002213790	-3.945517310	1.232782790
C	1.212375500	-3.992558700	0.485736430
C	-2.396457380	0.685436600	3.324243590
C	-2.396929010	-0.685002920	3.323912570
C	-3.103586970	-1.406240860	2.295366540
C	1.203295980	1.415737550	3.718095980
C	-0.001382170	0.732498920	4.038344350
C	-1.205387540	1.416591500	3.717347210

References

1. Gagne, O., Bond-length distributions for ions bonded to oxygen: results for the lanthanides and actinides and discussion of the f-block contraction. *Acta Cryst. B* **2018**, *74*, 49-62.
2. Blagg, R. J.; Muryn, C. A.; McInnes, E. J. L.; Tuna, F.; Winpenny, R. E. P., Single Pyramid Magnets: Dy_5 Pyramids with Slow Magnetic Relaxation to 40 K. *Angew. Chem. Int. Ed.* **2011**, *50*, 6530-6533.
3. Sanden, T.; Gamer, M. T.; Fagin, A. A.; Chudakova, V. A.; Konchenko, S. N.; Fedushkin, I. L.; Roesky, P. W., Synthesis of Unsupported Ln–Ga Bonds by Salt Metathesis and Ga–Ga Bond Reduction. *Organometallics* **2012**, *31*, 4331-4339.
4. Steele, L. A. M.; Boyle, T. J.; Kemp, R. A.; Moore, C., The selective insertion of carbon dioxide into a lanthanide(III) 2,6-di-t-butyl-phenoxide bond. *Polyhedron* **2012**, *42*, 258-264.
5. Blagg, R. J.; Ungur, L.; Tuna, F.; Speak, J.; Comar, P.; Collison, D.; Wernsdorfer, W.; McInnes, E. J. L.; Chibotaru, L. F.; Winpenny, R. E. P., Magnetic relaxation pathways in lanthanide single-molecule magnets. *Nat. Chem.* **2013**, *5*, 673-678.
6. Wooles, A. J.; Cooper, O. J.; McMaster, J.; Lewis, W.; Blake, A. J.; Liddle, S. T., Synthesis and Characterization of Dysprosium and Lanthanum Bis(iminophosphorano)methanide and -methanediide Complexes. *Organometallics* **2010**, *29*, 2315-2321.
7. Cui, D.; Nishiura, M.; Tardif, O.; Hou, Z., Rare-Earth-Metal Mixed Hydride/Aryloxide Complexes Bearing Mono(cyclopentadienyl) Ligands. Synthesis, CO2 Fixation, and Catalysis on Copolymerization of CO2 with Cyclohexene Oxide. *Organometallics* **2008**, *27*, 2428-2435.
8. Le Bris, J.; Hubert Pfalzgraf, L. G.; Rolland, M.; Garcia, Y., Synthesis, molecular structure and magnetic behaviour of Dy8(μ3,η2-OR)6(μ,η2-OR)4(η1-OR)8(μ,η1-OR)6 R=C2H4OPri. *Inorg. Chem. Commun.* **2006**, *9*, 695-698.
9. Boyle, T. J.; Bunge, S. D.; Clem, P. G.; Richardson, J.; Dawley, J. T.; Ottley, L. A. M.; Rodriguez, M. A.; Tuttle, B. A.; Avilucea, G. R.; Tissot, R. G., Synthesis and Characterization of a Family of Structurally Characterized Dysprosium Alkoxides for Improved Fatigue-Resistance Characteristics of PDyZT Thin Films. *Inorg. Chem.* **2005**, *44*, 1588-1600.
10. Yu, K.-X.; Ding, Y.-S.; Han, T.; Leng, J.-D.; Zheng, Y.-Z., Magnetic relaxations in four-coordinate Dy(III) complexes: effects of anionic surroundings and short Dy-O bonds. *Inorg. Chem. Front.* **2016**, *3*, 1028-1034.
11. Chai, J.; Jancik, V.; Singh, S.; Zhu, H.; He, C.; Roesky, H. W.; Schmidt, H.-G.; Noltemeyer, M.; Hosmane, N. S., Synthesis of a New Class of Compounds Containing a Ln−O−Al Arrangement and Their Reactions and Catalytic Properties. *J. Am. Chem. Soc.* **2005**, *127*, 7521-7528.
12. Herrmann, W. A.; Anwander, R.; Scherer, W., Lanthanoiden-Komplexe, V[1]. Strukturchemie ein- und zweikerniger Seltenerdalkoxide. *Chem. Ber.* **1993**, *126*, 1533-1539.
13. Fieser, M. E.; Palumbo, C. T.; La Pierre, H. S.; Halter, D. P.; Voora, V. K.; Ziller, J. W.; Furche, F.; Meyer, K.; Evans, W. J., Comparisons of lanthanide/actinide +2 ions in a tris(aryloxide)arene coordination environment. *Chem. Sci.* **2017**, *8*, 7424-7433.
14. Xiong, J.; Ding, H.-Y.; Meng, Y.-S.; Gao, C.; Zhang, X.-J.; Meng, Z.-S.; Zhang, Y.-Q.; Shi, W.; Wang, B.-W.; Gao, S., Hydroxide-bridged five-coordinate Dy^{III} single-molecule magnet exhibiting the record thermal relaxation barrier of magnetization among lanthanide-only dimers. *Chem. Sci.* **2017**, *8*, 1288-1294.
15. Mercado, B. Q.; Stuart, M. A.; Mackey, M. A.; Pickens, J. E.; Confait, B. S.; Stevenson, S.; Easterling, M. L.; Valencia, R.; Rodriguez-Fortea, A.; Poblet, J. M.; Olmstead, M. M.; Balch, A. L., $Sc_2(\mu_2\text{-}O)$ Trapped in a Fullerene Cage: The Isolation and Structural Characterization of $Sc_2(\mu_2\text{-}O)@C_s(6)\text{-}C_{82}$ and the Relevance of the Thermal and Entropic Effects in Fullerene Isomer Selection. *J. Am. Chem. Soc.* **2010**, *132*, 12098-12105.

16. Tang, Q.; Abella, L.; Hao, Y.; Li, X.; Wan, Y.; Rodríguez-Fortea, A.; Poblet, J. M.; Feng, L.; Chen, N., $Sc_2O@C_{3v}(8)-C_{82}$: A Missing Isomer of $Sc_2O@C_{82}$. *Inorg. Chem.* **2016**, *55*, 1926–1933.
17. Mercado, B. Q.; Chen, N.; Rodriguez-Fortea, A.; Mackey, M. A.; Stevenson, S.; Echegoyen, L.; Poblet, J. M.; Olmstead, M. M.; Balch, A. L., The Shape of the $Sc_2(\mu_2\text{-}S)$ Unit Trapped in C_{82}: Crystallographic, Computational, and Electrochemical Studies of the Isomers, $Sc_2(\mu_2\text{-}S)@C_s(6)-C_{82}$ and $Sc_2(\mu_2\text{-}S)@C_{3v}(8)-C_{82}$. *J. Am. Chem. Soc.* **2011**, *133*, 6752-6760.
18. Chen, C.-H.; Krylov, D. S.; Avdoshenko, S. M.; Liu, F.; Spree, L.; Yadav, R.; Alvertis, A.; Hozoi, L.; Nenkov, K.; Kostanyan, A.; Greber, T.; Wolter, A. U. B.; Popov, A. A., Selective arc-discharge synthesis of Dy_2S-clusterfullerenes and their isomer-dependent single molecule magnetism. *Chem. Sci.* **2017**, *8*, 6451-6465.
19. Kurihara, H.; Lu, X.; Iiduka, Y.; Nikawa, H.; Hachiya, M.; Mizorogi, N.; Slanina, Z.; Tsuchiya, T.; Nagase, S.; Akasaka, T., X-ray Structures of $Sc_2C_2@C_{2n}$ (n = 40-42): In-Depth Understanding of the Core-Shell Interplay in Carbide Cluster Metallofullerenes. *Inorg. Chem.* **2012**, *51*, 746-750.
20. Liu, F.; Wei, T.; Wang, S.; Guan, J.; Lu, X.; Yang, S., A Bent Tb_2C_2 Cluster Encaged in a $C_s(6)-C_{82}$ Cage: Synthesis, Isolation and X-ray Crystallographic Study. *Fullerenes, Nanotubes and Carbon Nanostructures* **2014**, *22*, 215-226.
21. Sado, Y.; Aoyagi, S.; Izumi, N.; Kitaura, R.; Kowalczyk, T.; Wang, J.; Irle, S.; Nishibori, E.; Sugimoto, K.; Shinohara, H., Structure of Tm_2 and Tm_2C_2 encapsulated in low-symmetry $C_{82}(C_s(6))$ fullerene cage by single crystal X-ray diffraction. *Chem. Phys. Lett.* **2014**, *600*, 38-42.
22. Wei, T.; Wang, S.; Liu, F.; Tan, Y.; Zhu, X.; Xie, S.; Yang, S., Capturing the Long-Sought Small-Bandgap Endohedral Fullerene $Sc_3N@C_{82}$ with Low Kinetic Stability. *J. Am. Chem. Soc.* **2015**, *137*, 3119–3123.
23. Shen, W.; Bao, L. B.; Hu, S.; Gao, X.; Xie, Y.; Gao, X.; Huang, W.; Lu, X., Isolation and Crystallographic Characterization of $Lu_3N@C_{2n}$ (2n = 80-88): Cage Selection by Cluster Size. *Chem.-Eur. J.* **2018**, *24*, 16692-16698.
24. Yang, S.; Chen, C.; Liu, F.; Xie, Y.; Li, F.; Jiao, M.; Suzuki, M.; Wei, T.; Wang, S.; Chen, Z.; Lu, X.; Akasaka, T., An Improbable Monometallic Cluster Entrapped in a Popular Fullerene Cage: $YCN@C_s(6)-C_{82}$. *Sci. Rep.* **2013**, *3*, 1487.
25. Liu, F.; Gao, C.-L.; Deng, Q.; Zhu, X.; Kostanyan, A.; Westerström, R.; Wang, S.; Tan, Y.-Z.; Tao, J.; Xie, S.-Y.; Popov, A. A.; Greber, T.; Yang, S., Triangular Monometallic Cyanide Cluster Entrapped in Carbon Cage with Geometry-Dependent Molecular Magnetism. *J. Am. Chem. Soc.* **2016**, *138*, 14764–14771.
26. Pan, C.; Shen, W.; Yang, L.; Bao, L.; Wei, Z.; Jin, P.; Fang, H.; Xie, Y.-P.; Akasaka, T.; Lu, X., Crystallographic Characterization of Y_2C_{2n} ($2n$ = 82, 88-94): Direct Y-Y Bonding and Cage-Dependent Cluster Evolution. *Chem. Sci.* **2019**.
27. Olmstead, M. M.; de Bettencourt-Dias, A.; Stevenson, S.; Dorn, H. C.; Balch, A. L., Crystallographic characterization of the structure of the endohedral fullerene {$Er_2@C_{82}$ Isomer I} with C_s cage symmetry and multiple sites for erbium along a band of ten contiguous hexagons. *J. Am. Chem. Soc.* **2002**, *124*, 4172-4173.
28. Olmstead, M. M.; Lee, H. M.; Stevenson, S.; Dorn, H. C.; Balch, A. L., Crystallographic characterization of Isomer 2 of $Er_2@C_{82}$ and comparison with Isomer 1 of $Er_2@C_{82}$. *Chem. Commun.* **2002**, 2688-2689.
29. Shen, W.; Bao, L.; Wu, Y.; Pan, C.; Zhao, S.; Fang, H.; Xie, Y.; Jin, P.; Peng, P.; Li, F.-F.; Lu, X., $Lu_2@C_{2n}$ ($2n$ = 82, 84, 86): Crystallographic Evidence of Direct Lu–Lu Bonding between Two Divalent Lutetium Ions Inside Fullerene Cages. *J. Am. Chem. Soc.* **2017**, *139*, 9979-9984.
30. Suzuki, M.; Yamada, M.; Maeda, Y.; Sato, S.; Takano, Y.; Uhlík, F.; Slanina, Z.; Lian, Y.; Lu, X.; Nagase, S.; Olmstead, M. M.; Balch, A. L.; Akasaka, T., The Unanticipated Dimerization of $Ce@C_{2v}(9)-C_{82}$

upon Co-crystallization with Ni(octaethylporphyrin) and Comparison with Monomeric M@C_{2v}(9)-C_{82} (M = La, Sc, and Y). *Chem.-Eur. J.* **2016**, DOI: 10.1002/chem.201602595.
31. Bao, L.; Pan, C.; Slanina, Z.; Uhlik, F.; Akasaka, T.; Lu, X., Isolation and Crystallographic Characterization of the Labile Isomer of Y@C_{82} Cocrystallized with Ni(OEP): Unprecedented Dimerization of Pristine Metallofullerenes. *Angew. Chem. Int. Ed.* **2016**, *55*, 9234-9238.
32. Sato, S.; Nikawa, H.; Seki, S.; Wang, L.; Luo, G.; Lu, J.; Haranaka, M.; Tsuchiya, T.; Nagase, S.; Akasaka, T., A Co-Crystal Composed of the Paramagnetic Endohedral Metallofullerene La@C_{82} and a Nickel Porphyrin with High Electron Mobility. *Angew. Chem. Int. Ed.* **2012**, *51*, 1589-1591.
33. Yang, H.; Jin, H.; Wang, X.; Liu, Z.; Yu, M.; Zhao, F.; Mercado, B. Q.; Olmstead, M. M.; Balch, A. L., X-ray Crystallographic Characterization of New Soluble Endohedral Fullerenes Utilizing the Popular C_{82} Bucky Cage. Isolation and Structural Characterization of Sm@C_{3v}(7)-C_{82}, Sm@C_s(6)-C_{82}, and Sm@C_2(5)-C_{82}. *J. Am. Chem. Soc.* **2012**, *134*, 14127-14136
34. Hu, Z.; Hao, Y.; Slanina, Z.; Gu, Z.; Shi, Z.; Uhlík, F.; Zhao, Y.; Feng, L., Popular C_{82} Fullerene Cage Encapsulating a Divalent Metal Ion Sm^{2+}: Structure and Electrochemistry. *Inorg. Chem.* **2015**, *54*, 2103-2108.
35. Suzuki, M.; Lu, X.; Sato, S.; Nikawa, H.; Mizorogi, N.; Slanina, Z.; Tsuchiya, T.; Nagase, S.; Akasaka, T., Where Does the Metal Cation Stay in Gd@C_{2v}(9)-C_{82}? A Single-Crystal X-ray Diffraction Study. *Inorg. Chem.* **2012**, *51*, 5270–5273.
36. Hu, S.; Liu, T.; Shen, W.; Slanina, Z.; Akasaka, T.; Xie, Y.; Uhlik, F.; Huang, W.; Lu, X., Isolation and Structural Characterization of Er@C_{2v}(9)-C_{82} and Er@C_s(6)-C_{82}: Regioselective Dimerization of a Pristine Endohedral Metallofullerene Induced by Cage Symmetry. *Inorg. Chem.* **2019**, *58*, 2177-2182.
37. Sado, Y.; Aoyagi, S.; Kitaura, R.; Miyata, Y.; Nishibori, E.; Sawa, H.; Sugimoto, K.; Shinohara, H., Structure of Tm@C_{82}(I) Metallofullerene by Single-Crystal X-ray Diffraction Using the 1:2 Co-Crystal with Octaethylporphyrin Nickel (Ni(OEP)). *J. Phys. Chem. C* **2013**, *117*, 6437-6442.
38. Suzuki, M.; Slanina, Z.; Mizorogi, N.; Lu, X.; Nagase, S.; Olmstead, M. M.; Balch, A. L.; Akasaka, T., Single-Crystal X-ray Diffraction Study of Three Yb@C_{82} Isomers Cocrystallized with Ni^{II}(octaethylporphyrin). *J. Am. Chem. Soc.* **2012**, *134*, 18772–18778.
39. Wang, Y.; Morales-Martínez, R.; Zhang, X.; Yang, W.; Wang, Y.; Rodríguez-Fortea, A.; Poblet, J. M.; Feng, L.; Wang, S.; Chen, N., Unique Four-Electron Metal-to-Cage Charge Transfer of Th to a C_{82} Fullerene Cage: Complete Structural Characterization of Th@C_{3v}(8)-C_{82}. *J. Am. Chem. Soc.* **2017**, *139*, 5110-5116.
40. Cai, W.; Morales-Martínez, R.; Zhang, X.; Najera, D.; Romero, E. L.; Metta-Magaña, A.; Rodríguez-Fortea, A.; Fortier, S.; Chen, N.; Poblet, J. M.; Echegoyen, L., Single crystal structures and theoretical calculations of uranium endohedral metallofullerenes (U@C_{2n}, $2n$ = 74, 82) show cage isomer dependent oxidation states for U. *Chem. Sci.* **2017**, *8*, 5282-5290.
